# Assessments Related to the Physical, Affective and Cognitive Domains of Physical Literacy Amongst Children Aged 7–11.9 Years: A Systematic Review

**DOI:** 10.1186/s40798-021-00324-8

**Published:** 2021-05-27

**Authors:** Cara Shearer, Hannah R. Goss, Lynne M. Boddy, Zoe R. Knowles, Elizabeth J. Durden-Myers, Lawrence Foweather

**Affiliations:** 1 Physical Activity Exchange, Research Institute for Sport and Exercise Sciences, Liverpool John Moores University, 5 Primrose Hill, Liverpool, UK; 2 School of Health & Human Performance, Dublin City University, Dublin, Ireland; 3 Faculty of Education, The University of Gloucestershire, Francis Close Hall, Swindon Road, Cheltenham, UK

**Keywords:** Physical literacy, Assessment, Physical education, Children, Systematic review

## Abstract

**Background:**

Over the past decade, there has been increased interest amongst researchers, practitioners and policymakers in physical literacy for children and young people and the assessment of the concept within physical education (PE). This systematic review aimed to identify tools to assess physical literacy and its physical, cognitive and affective domains within children aged 7–11.9 years, and to examine the measurement properties, feasibility and elements of physical literacy assessed within each tool.

**Methods:**

Six databases (EBSCO host platform, MEDLINE, PsycINFO, Scopus, Education Research Complete, SPORTDiscus) were searched up to 10th September 2020. Studies were included if they sampled children aged between 7 and 11.9 years, employed field-based assessments of physical literacy and/or related affective, physical or cognitive domains, reported measurement properties (quantitative) or theoretical development (qualitative), and were published in English in peer-reviewed journals. The methodological quality and measurement properties of studies and assessment tools were appraised using the COnsensus-based Standards for the selection of health Measurement INstruments risk of bias checklist. The feasibility of each assessment was considered using a utility matrix and elements of physical literacy element were recorded using a descriptive checklist.

**Results:**

The search strategy resulted in a total of 11467 initial results. After full text screening, 11 studies (3 assessments) related to explicit physical literacy assessments. Forty-four studies (32 assessments) were relevant to the affective domain, 31 studies (15 assessments) were relevant to the physical domain and 2 studies (2 assessments) were included within the cognitive domain. Methodological quality and reporting of measurement properties within the included studies were mixed. The Canadian Assessment of Physical Literacy-2 and the Passport For Life had evidence of acceptable measurement properties from studies of very good methodological quality and assessed a wide range of physical literacy elements. Feasibility results indicated that many tools would be suitable for a primary PE setting, though some require a level of expertise to administer and score that would require training.

**Conclusions:**

This review has identified a number of existing assessments that could be useful in a physical literacy assessment approach within PE and provides further information to empower researchers and practitioners to make informed decisions when selecting the most appropriate assessment for their needs, purpose and context. The review indicates that researchers and tool developers should aim to improve the methodological quality and reporting of measurement properties of assessments to better inform the field.

**Trial registration:**

PROSPERO: CRD42017062217

**Supplementary Information:**

The online version contains supplementary material available at 10.1186/s40798-021-00324-8.

## Key Points

- This systematic review identified 52 existing assessment tools related to the physical, affective and cognitive domains of physical literacy for use in children aged 7–11.9 years old.
- Only three explicit (self-titled) physical literacy assessments were found. While these more comprehensive assessments show promise, more studies are needed to demonstrate their methodological rigour and feasibility for use in primary school settings.
- This review identified a number of valid, reliable and feasible measures of elements of the physical and affective domains that could be useful in a pragmatic physical literacy assessment approach within physical education. More assessment development work is needed with regards to measuring the cognitive domain of physical literacy.
- Findings indicate that researchers and tool developers should aim to improve the methodological quality and reporting of measurement properties of assessments.

## Background

The concept of physical literacy has attracted significant attention from researchers, policymakers and practitioners within education, sport and public health sectors and features prominently within current national and international sport and physical activity policies and strategic plans [1–16]. While physical literacy is a term that has been around since the late 19^th^ Century [17], current interest stems from the work of Whitehead [18–20], who first introduced the concept as a way forward to address low levels of physical activity around the world and as a reaction to a perceived focus on high performance and elitism within physical education (PE), to the detriment of the health and well-being of less-abled students. Whitehead most recently described physical literacy as “the motivation, confidence, physical competence, knowledge and understanding to value and take responsibility for engagement in physical activities for life” ([21], p8), though her original conceptualisation of physical literacy [18, 19], grounded in the philosophical traditions of phenomenology, existentialism and monism, has evolved into an increasingly fluid concept subject to varying levels of abstraction and alignment in deployment by researchers and practitioners [3]. Indeed, physical literacy is a contested term [1, 22], with various contextually sensitive definitions and interpretations of the concept proposed internationally [1–3, 6–8, 17, 23–26]. Nevertheless, taken together, these diverse definitions seem to reflect a holistic view of physical literacy that emphasises affective, physical and cognitive attributes and predispositions necessary to participate in physical activity across the life course [3, 4, 25]. Furthermore, most researchers and practitioners advocating for physical literacy agree that such an approach is inclusive and encourages more diverse forms of engagement in physical activity, and so would be more likely to lead to life-long safe, committed engagement in physical activity, and better health, well-being and quality of life for all [6, 7, 17, 27, 28].

The majority of existing physical literacy research has focussed on children and youth populations within school settings [1]. Across the majority of Western countries, school attendance within the 7–11-year-old age range is compulsory, thus making primary schools an optimal setting for physical activity promotion. While physical literacy is recognised as a lifelong concept, the heightened attention on childhood reflects the fact that this is seen as a critical stage for the development of important physical literacy attributes necessary for lifelong physical activity, health and well-being [29]. Schools are considered to be nurturing environments where children have opportunities to be active, learn about physical activity and develop positive physical activity behaviours [30–32]. As a result, physical literacy has been identified as a guiding framework and overarching goal of quality PE and a major focus of PE curriculum internationally [33–36]. In England, the National Curriculum for PE aims to ensure that all pupils develop competence to excel in a broad range of physical activities, are physically active for sustained periods of time, engage in competitive sports/activities and lead healthy, active lives [37]. These ambitions align with the concept of physical literacy. As such, a cross-government action plan positioned physical literacy as a core element of early learning and stated that physical literacy should be a fundamental part of every child’s school experience [38].

Throughout compulsory education, assessment - both formative and summative - is a critical aspect of pedagogical practice and accountability systems [39–41]. For the purposes of this review and in accordance with Edwards et al. [42], we define *assessment* as it is widely understood and used within educational contexts: as an umbrella term for measurement, charting, monitoring, tracking, evaluating, characterising, observing, indicating, and so on. Appropriate assessment of childhood physical literacy in PE on both an individual and population level could improve standards and expectations, and raise the profile of both PE and physical literacy [43, 44]. Primary teachers report that assessment in PE provides a structure and focus to planning, teaching and learning, which positively impacts on both the teacher and child [45]. Thus, the classroom teacher, utilising the close relationship formed between teacher and pupil, should be empowered to implement an assessment of physical literacy, fulfilling roles such as charting progress, providing feedback, and highlighting key areas for how a child may develop their physical literacy over time [46–50]. Teachers themselves have, however, cited barriers to implementing assessment in PE such as the lack of priority given to PE within the curriculum; limited time, space and expertise [51, 52]; difficulty in assessment differentiation and limited availability of comparator samples [45]; and varied beliefs, understandings and engagement regarding assessment [39, 40], alongside limited knowledge of physical literacy [53]. Thus, considering the feasibility of a physical literacy assessment tool is of vital importance when determining appropriate use within educational contexts [54].

Effective assessment of physical literacy in PE will enable funders, policymakers, researchers and educators to understand what teaching, learning and curriculum strategies are most effective in helping support physical literacy [27, 44]. Despite this assertion, divergent approaches to understanding the concept of physical literacy have led to tensions in the research literature surrounding whether physical literacy can and should be assessed, with implications for how assessment has been operationalised in practice [5, 17, 18, 42, 47, 55, 56]. Edwards et al. [42] suggested that idealist approaches to the concept of physical literacy, and therefore assessment, view physical literacy as holistic with inseparable dimensions and as a complex and dynamic process unique to each individual. Assessment can therefore only be captured through subjective, qualitative, interpretivist methods and is centred on an assessment-for-learning approach to monitor progress relative to the individual student’s physical literacy journey [17, 42, 48]. At the other end of the debate are pragmatic approaches that view physical literacy as a concept that can and should be assessed for the purposes of evidence-based practice and accountability, with positivist, reductionist measurement methods typically utilised [42]. Barnett et al. [54] suggested that these approaches do not need to be mutually exclusive: while acknowledging the holistic nature of physical literacy, they suggested that existing measures of physical literacy elements should not be dismissed if they do not capture the entirety of the concept; rather PE teachers should be encouraged to recognise this limitation and evaluate the completeness of their assessment approaches. Similarly, Essiet et al. [57] proposed that a comprehensive quantitative assessment of physical literacy for teachers can be possible through an aggregate measure of all the elements and domains identified within the corresponding definition. Thus, identifying assessments of physical literacy and/or its affective (motivation and confidence), physical, and cognitive (knowledge and understanding) domains, inclusive of idealist and pragmatic approaches to the concept, can inform physical literacy assessment efforts within primary (elementary) PE.

Barnett and colleagues [54] produced a decision-making guide for researchers and teachers for the assessment of physical literacy within the context of school PE and within the parameters of the Australian definition of physical literacy [16]. This guidance outlined key considerations to inform what assessment approach to choose, including factors such as the physical literacy elements of importance (what is being measured and what is being missed), the purpose of conducting the assessment, the assessment context and the target age range. Barnett et al. [54] recognised that there was not an “ideal” approach to measurement and therefore the guidance was aimed at empowering teachers and researchers to make informed decisions on how to assess physical literacy based on their intentions, needs and resources. It was beyond the remit of the study to review all potential assessments that could align with physical literacy domains and consider whether existing assessments/measures were reliable, valid, and trustworthy. Edwards et al. [42] conducted a systematic review of the literature and identified 52 assessments of physical literacy and related constructs evaluating these in relation to age group, environment, and philosophy. While several qualitative and quantitative tools were identified for the assessment of affective, cognitive and physical domains as well as the related construct of physical activity for use with children under 12 years old, few assessments captured the entire range of domains [42]. Within their review, Edwards and colleagues used the global search term “physical literacy” to identify assessments. There is scope to expand this review through the use of wider search terms related to the elements within affective (e.g. motivation and confidence), cognitive (e.g. knowledge and understanding) and physical (e.g. motor skills) domains of physical literacy, which could identify other relevant assessment options for consideration in assessment discourses. Furthermore, since this review was published, a number of explicit assessments of physical literacy have been developed, such as the Passport for Life [58] and Physical Literacy Assessment for Youth [59], that warrant further consideration. It was outside of the scope of the Edwards et al. [42] review to consider the measurement properties (i.e. validity, reliability, trustworthiness) and feasibility of each assessment. We believe that providing researchers and teachers with information in a single point of reference on the theoretical development, measurement properties and feasibility of assessments of physical literacy and its elements within PE contexts will further empower them to make informed decisions on selecting an appropriate assessment. Such information could assist with the development of a bank of assessment resources and guide potential physical literacy assessment development in the field.

The aim of this study, therefore, is to systematically review the scientific literature for tools to assess physical literacy and its physical, cognitive and affective domains within children aged 7–11.9 years. We selected this age group as it represents the lower and upper ages for children within Key Stage 2 of the National Curriculum in England [37] with the aim of informing PE assessments within this block of education (i.e. school years 3 to 6). This paper will explore and critically discuss each assessment tool to appraise its (a) measurement properties, (b) physical literacy elements assessed and (c) feasibility for use within a primary school setting.

## Methods

This study followed the Preferred Reporting Items for Systematic Reviews and Meta-Analyses (PRISMA) [60]. The protocol information for this review was registered with PROSPERO, reference: CRD42017062217.

### Inclusion Criteria

The full PICOS statement can be found in Additional file 1. Studies were included if they:

1. Sampled typically developing children with a reported mean age or age range between 7–11.9 years (including overweight and obese children and children from deprived areas).
2. Reported on a field-based assessment tool (i.e. not measured through laboratory methods) within PE or related contexts (such as physical activity, sport, active play, exercise or recreation) with an outcome relating to physical literacy (see PICOS statement for the list of outcomes (Additional file 1). Other contexts were considered in order to capture assessments that could be suitable for use in school settings and PE.
3. Cross-sectional, longitudinal or experimental study design.
4. Reported a measurement method (qualitative or quantitative) relevant to physical literacy and/or an element of physical literacy.
5. Reported information on measurement properties (quantitative assessments) or theoretical development (qualitative assessments).
6. Published in English and in a peer-reviewed journal.

### Exclusion Criteria

Studies identified through the literature search were excluded if:

1. Included special populations (i.e. children with developmental coordination disorder, diagnosed with learning difficulty).
2. Lab-based assessment.
3. Book chapters, case studies, student dissertations, conference abstracts, review articles, meta-analyses, editorials, protocol papers and systematic reviews.
4. Full text articles were not available.

### Information Sources

Relevant studies were identified by means of electronic searches on EBSCOhost and through scanning reference lists of included articles. The EBSCOhost platform supplied access to MEDLINE, PsycINFO, Scopus, Education Research Complete and SPORTDiscus databases. Each of the databases was searched independently. Publication date restrictions were not applied in any search with the final search conducted on 10th September 2020.

### Search Strategy and Study Selection

Search strategies used in the databases included combinations of key search terms which were divided into four sections: tool (Assessment OR Measurement OR Test OR Tool OR Instrument OR Battery OR Method OR Psychometric OR Observation OR Indicator OR Evaluate OR Valid Or Reliable) AND context (“Physical Activity” OR “Physical Literacy” OR Play OR Sport OR “Physical Education” OR Exercise OR Recreation) AND population (Child OR Youth OR Adolescent OR Paediatric OR Schoolchild OR Boy OR Girl OR Preschool OR Juvenile OR Teenager) AND physical literacy elements (Motivation OR Enjoyment OR Confidence OR Self* Or “Perceived Competence” OR Affective OR Social OR Emotion* OR Attitude* OR Belief* OR Physical* OR Fitness OR Motor OR Movement* OR Skills* OR Technique* OR Mastery OR Ability* OR Coordination OR Performance OR “Perceptual Motor” OR Knowledge OR Understanding OR Value OR Cognition* OR Health OR Wellbeing*). Boolean searches were carried out using “AND” to combine concepts (tool, context, population, element) and narrow the search to only capture articles in which all relevant concepts appear (see Additional file 2 for an example search strand). Following the initial search, all records were exported to Covidence (Covidence systematic review software, Veritas Health Innovation) for screening (Covidence data/reports are available from the contact author upon reasonable request). Duplicates were removed using Endnote and the two lead authors (CS and HG) screened all titles and abstracts. Only articles published or accepted for publication in peer-reviewed journals were considered. A third author (LF) checked decisions on what to include based on the inclusion/exclusion criteria (i.e. age range, typically developing population, field-based assessment, study design, physical literacy element, measurement properties and peer-reviewed status) and any disagreements were resolved by discussion and collaboration with all authors. Full-text articles were further evaluated separately for relevance by the two lead authors (CS and HG) and labelled “yes”, “no”, or “maybe”. The two reviewers conferred and, following discussion on any inconsistencies, agreement was reached on all articles. A third reviewer (LF) checked all of the studies that met the inclusion criteria and 10% of studies that were excluded to ensure accuracy in the study selection process. All decisions were made in closed meetings with no recorded minutes and are attributable to the authors. Where a manual was available for an assessment that met the inclusion criteria, these were accessed if the manual was freely available online or, alternatively, through contacting the study authors where possible.

### Data Collection Processes

Due to the large number of studies included after full text screening, the studies were divided into explicit physical literacy assessments and related physical, affective, and cognitive domains in accordance with definitions and conceptualisations of physical literacy [1, 2, 6, 16, 20, 26]. This categorisation of assessments of elements into domains was undertaken in order to position assessments into familiar categories known to potential assessment users (e.g. coaches, researchers and teachers in physical literacy and physical education) and for ease of interpretation. The lead authors (CS physical and physical literacy; HG affective and cognitive) independently extracted individual study data relating to study information (authors, publication date, country and study design), sample description, purpose of study, the physical literacy element being assessed (as described by the study authors themselves), measurement technique (i.e. interviews, questionnaires, practical trial), outcome variables, measurement properties/theoretical development and utility information (reliability, validity, responsiveness and feasibility). Data extraction was checked for accuracy for the first three studies across each domain by a third reviewer (LF) and any inconsistencies were resolved following discussion with the lead authors.

#### Quality Appraisal

The COnsensus-based Standards for the selection of health Measurement INstruments (COSMIN) checklist was used to evaluate the methodological rigour of assessments [61, 62]. The COSMIN checklist has been developed by a team of international multidisciplinary researchers and is of a modular design, which enabled flexibility to suit the needs of the current systematic review. Using the COSMIN risk of bias checklist [61] each measurement property (content validity, construct validity, internal consistency, cross-cultural validity, test retest reliability, intra-rater reliability, inter-rater reliability, criterion validity) was appraised for methodological quality and subsequently given a rating of “very good”, “adequate”, “doubtful”, or “inadequate” or, if not reported, “NR”. This 4-point rating scale and worst score counts method were used throughout. Where the reporting of measurement properties received a rating of “very good”, the validity and reliability of the tool can be appraised using established thresholds [63] (see Additional file 3). The lead authors (CS physical and physical literacy assessments; HG affective and cognitive assessments) independently appraised measurement properties; a third reviewer checked 10% of measurement quality ratings and threshold scoring for accuracy and any uncertainties were discussed and agreed upon in face-to-face meetings with all three reviewers (CS, HG, LF). The COSMIN guidelines were updated during the review process and new guidance regarding the importance of each measurement property was detailed [62]. According to the updated guidelines, if neither the original study, an associated paper or the tool manual adequately describes the measurement development process and/or aspects of content validity, then the tool should not be appraised by researchers further in relation to wider measurement properties. We elected to follow the previous guidelines and made a conscious decision to appraise all the available measurement properties within all the eligible studies in order to be inclusive and present a detailed overview of what assessments are available. As qualitative assessments were also eligible for inclusion, the National Institute for Healthcare and Excellence (NICE) Quality Appraisal Checklist for qualitative studies [64] was identified as a tool to appraise the methodological rigour of these assessments.

The feasibility of each assessment tool, including factors such as cost-efficiency (time, space, equipment, training and qualifications required) and acceptability (participant understanding, completed assessments), was appraised using a utility matrix developed from previous research [65, 66] (see Table 1). Each dimension of feasibility was independently scored on a 1* (low feasibility) to 4* (high feasibility) scale using information reported within included studies and manuals. An overall feasibility utility matrix score was also calculated by summing the scores from each of the seven feasibility items to allow comparisons between assessments (maximum feasibility score = 28).

**Table 1:** Description of rating of feasibility of assessments

		****(High feasibility)	***	**	* (Low feasibility)
Cost Efficiency	How long does an assessment take to complete	*< 15 min*	*< 30 min*	*30–60 min*	*> 60 min*
	How much space is needed to administer an assessment?	*Less than 6 m, a corner of a room*	*6–10 m, a standard room*	*10-20 m (primary school sports hall)*	*20 m+ (secondary school sports hall requirement)*
	What equipment is required to administer an assessment?	*Equipment likely to be present in a typical school*	*Some extra equipment or resource required would be additional to what is typically present (primary school)*	*Most of the equipment required would be additional to what is typically present (primary school)*	*All equipment required would be additional to what is typically present (primary school)*
	What qualification is required to administer an assessment?	*Able to be administered by any school staff*	*Able to be administered by a qualified teacher*	*Able to be administered by PE/Sport specialist*	*Requires researcher with specific higher qualifications*
	What training is required to administer an assessment?	*Little or no additional training required*	*Some additional training required (less than half a day)*	*Further additional training required (half a day to one and a half days)*	*Significant training required (more than one and a half days)*
Acceptability	Is there evidence of participant understanding?	*Investigation of participant understanding (evidence from participants)*	*Estimated evidence of participant understanding (evidence from source other than participant)*	*Participant understanding not explicitly stated but can be assumed*	*No evidence of participant understanding*
	How many assessments are not completed?	*Low number of missing items (< 10%) and adequate response rate (> 40%)*	*High number of missing items (> 10%) and adequate response rate (> 40%)*	*Low number of missing items or poor (< 10%) and adequate response rate (< 40%)*	*High number of missing items (> 10%) and poor response rate (< 40%)*

A physical literacy element checklist was developed to highlight which aspects of physical literacy each assessment captured, as explicitly stated within the included studies and manuals. The checklist was developed by the research team through discussion in a closed meeting following an overview of international physical literacy literature [2] and utilised elements captured within various conceptualisations of physical literacy [1, 20, 26, 67–69]. The definitions adopted internationally were collated and cross-referenced, identifying distinctive characteristics of physical literacy referred to in research and policy. This process resulted in a checklist that included 10 affective, 20 physical and 11 cognitive physical literacy elements (Table 2).

**Table 2:** Physical literacy element checklist

Affective domain	Physical domain	Cognitive domain
Confidence Motivation Emotional regulation Enjoyment Persistence/resilience/commitment Adaptability Willingness to try new activities Autonomy Self-perception/self-esteem Perceived physical competence	Object-control Stability Locomotor Movement skills—land Movement skills—water Moving using equipment Cardiovascular endurance Muscular endurance Coordination Flexibility Agility Strength Reaction time Speed Power Rhythmic ability Aesthetic/expressive ability Sequencing Adapt movement strategies to the situation/environment Progression from simple-complex skills	Knowledge and understanding of benefits of physical activity Knowledge and understanding of importance of physical activity Knowledge and understanding of effects of physical activity on the body Knowledge and understanding of opportunities to be active Knowledge and understanding of sedentary behaviour Ability to identify and describe movement Creativity and imagination in application of movement Decision-making (ability to think, understand and make decisions, knowing how and when to perform) Ability to reflect and improve own performance, including setting optimal challenges Knowledge and understanding of tactics, rules, and strategy Knowledge and understanding of safety considerations and risk

Each of the included studies was independently scored for feasibility and checked for physical literacy elements by the two lead authors (CS and HG). As above, tools were divided into domains and scored separately by the lead authors (CS: physical and physical literacy; HG cognitive and affective). Each lead author (CS an HG) checked 10% of studies from the other lead author to ensure consistent methodological rigour of the feasibility and physical literacy element scoring. Any discrepancies were discussed and resolved in face-to-face meetings with the third reviewer (LF).

## Results

**Fig. 1:**
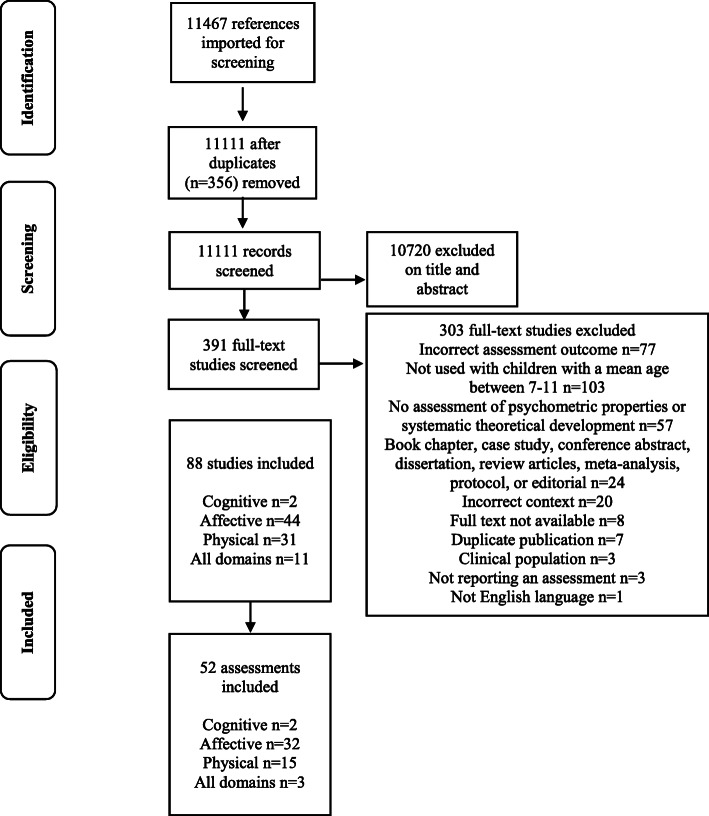
PRISMA flow diagram showing the process of study identification and selection

An overview of the search process is provided in Fig. 1. The search strategy resulted in a total of 11467 results (*NB*. this search strand was also used to identify assessments used in children aged 3–7.9 years, which will be reported elsewhere). After the screening of titles and abstracts, 391 articles were retrieved for full text reading. After full text screening was completed, in relation to the 7–11.9 years age range, a total of 88 eligible studies were included. Eleven studies [58, 70–79] and two corresponding manuals [59, 80] were found for explicit (self-titled) physical literacy assessments. We also found 44 studies related to the affective domain [81–124] with one corresponding manual [125], 31 studies [126–156] and six corresponding manuals [157–162] related to the physical domain, and two studies related to the cognitive domain [163, 164]. From these studies, a total of 52 distinct assessments were identified.

Three tools were explicitly labelled as physical literacy assessments (Canadian Assessment of Physical Literacy: CAPL-2 [70–77, 80]; Physical Literacy Assessment in Youth: PLAYfun [59, 79, 165]; Passport for Life: PFL [58]). Thirty-two tools assessed elements within the affective domain (Achievement Goal scale for Youth Sports: AGSYS [81]; ASK-KIDS [82–84]; Attitudes Towards Curriculum Physical Education: ATCPE [85]; Attitudes Towards Outdoor play scale: ATOP [86]; Adapted Behavioural Regulation in Exercise Questionnaire: BREQ [87]; Children’s Attraction to Physical Activity Questionnaire: CAPA [88–90]; Children’s Attitudes Towards Physical Activity: CATPA [91–93]; Commitment to Physical Activity Scale: CPAS [94]; Children and Youth Physical Self-Perception Profile: CY-PSPP [95, 96]; Motivational determinants of elementary school students’ participation in physical activity: DPAPI [97]; Enjoyment in Physical Education: EnjoyPE [98]; Food, Health and Choices Questionnaire: FHC-Q [99, 100]; Feelings About Physical Movement: FAPM [83]; Healthy Opportunities for Physical Activity and Nutrition Evaluation: HOP’N [101]; Lunchtime Enjoyment of Activity and Play Questionnaire: LEAP [102]; Momentary Assessment of Affect and Physical feeling states: MAAP [103]; Motivational Orientation in Sport Scale: MOSS [104, 105]; Negative Attitudes Towards Physical Activity Scale: NAS [106]; Physical Activity Beliefs and Motives: PABM [107]; Physical Activity Enjoyment Scale: PACES [108]; Physical activity and Healthy Food Efficacy: PAHFE [109]; Positive Attitudes Towards Physical Activity Scale: PAS [106]; Physical Activity Self-Efficacy Questionnaire: PASE [110]; Physical Activity Self-Efficacy Scale: PASES [111, 112]; Physical Activity Self-efficacy, Enjoyment, and Social Support Scale [113]; The Revised Perceived Locus of causality in physical Education: PLOC in PE [114]; Perceived Motivational Climate in Sport Questionnaire: PMCS [115]; Response to Challenge Scale: RCS [116–118]; Self-Efficacy Scale [119]; Self-Perception Profile for Children: SPPC [120–123, 125]; Trichotomous Achievement Goal Model: TAGM [124]; Task and Ego Orientation in Sport Questionnaire: TEOSQ [108, 115]). Fifteen tools assessed elements within the physical domain (ALPHA Fitness Battery: ALPHA [126, 157]; Athletic Skills Track: AST [127]; Bruininks–Oseretsky Test of Motor Proficiency 2^nd^ Edition Short Form: BOTMP-SF [128–130, 158]; EUROFIT [131, 159]; FITNESSGRAM [132–134, 160]; Golf Swing and Putt skill Assessment: GSPA [135]; Movement assessment battery for children-2: MABC-2 [136–139]; Motorische Basiskompetenzen in der 3: MOBAK-3 [140–143, 161]; Motorisk Utveckling som Grund för Inlärning: MUGI [166]; Obstacle Polygon: OP [145]; Physical Activity Research and Assessment tool for Garden Observation: PARAGON [146]; Star Excursion Balance Test: SEBT [147]; Stability skill test: SS [148]; Test of Gross Motor Development-3: TGMD-3 [149–155, 162]; Y Balance Test: YBT [156]). Two tools assessed elements within the cognitive domain (Beat Osteoporosis Now-Physical Activity Survey: BONES PAS [163]; Pupil Health Knowledge Assessment: PHKA [164]).

### Assessment Characteristics

Table 3 describes the characteristics of the 52 included assessment tools. The majority of assessments were developed in the USA (*n* = 28), Australia (*n* = 5) and Europe (*n* = 12). Notably, the three explicit (self-titled) physical literacy assessments—CAPL-2, PLAYfun and PFL—were all developed in Canada. PLAYfun is one component of a wider suite of physical literacy assessment in youth (PLAY) tools designed to assist with programme evaluation and research in sport, health and recreation, including PLAYbasic, PLAYfun, PLAYself, PLAYparent and PLAYcoach [59]. Studies were only found in relation to PLAYfun, which assesses eighteen motor skill tasks (including running, locomotor, upper body control, lower body control) by observation from trained assessors. The child’s confidence and comprehension towards the movement are also recorded. Confidence refers to whether the child had low, medium or high confidence when performing each task, while comprehension is assessed as to whether the child requires a prompt, mimics their peers, asks the assessor for a description or demonstration of the task. The assessor must have some education in movement or motion analysis and grades each child’s physical ability using a 100mm visual analogue scale, placing a mark in one of four categories: initial, emerging, competent and proficient. Scores of 100 on the scale represent “the best anyone can be at the skill, regardless of age” [59]. Scores across tasks are summed and then divided by 18 to generate the PLAYfun physical literacy score. The PFL is designed as a formative criterion-based assessment for PE practitioners and incorporates fitness and movement assessments (Plank, Lateral Bound, Four-Station Circuit, Run-Stop-Return, Throw and Catch with a Bounce, Advanced Kick) as well as questionnaires to assess active participation (22 self-report items relating to diversity, interests and intentions) and living skills (21 items relating to feelings, thinking and interacting skills). The fitness and movement assessments are scored by teachers using detailed rubrics that examine the technique and outcomes of the movements, with children placed into one of four categories: emerging, developing, acquired, accomplished. CAPL-2 was developed for monitoring and surveillance of physical literacy [67]. The CAPL-2 protocol integrates the measurement of physical competence (PACER test, Plank [70] and the Canadian Agility and Movement Skills Assessment: CAMSA [71]), which is worth 30 points, motivation and confidence (30 points), daily physical activity behaviour as assessed by self-report and daily pedometer step count (30 points) and knowledge and understanding (10 points). The knowledge and understanding component includes four questionnaire items and a missing word paragraph activity. Scores from domains are summed to create a CAPL-2 total score out of 100, which is used to classify the children into one of four interpretative categories (beginning, progressing, achieving or excelling) based on age and sex-specific cut points.

**Table 3:** Characteristics of physical literacy and related affective, physical and affective domain assessments

Assessment, *country of origin*, author(s) of primary study [citation], related studies [citation(s)]	Participant n, sex (% female) (age range; mean age)	Purpose/ use of assessment	Constructs assessed	Scale Scoring
***Explicit physical literacy assessments***				
**CAPL-2** *Canada* Longmuir et al. [70–77, 80]	*N* = 963 55% (8–10; 10.1 ± 1.17)	Assess physical literacy	CAMSA PACER (10m or 25m) Isometric plank hold Motivation and confidence are measured by a 12 items questionnaire that aggregate to four subscales (adequacy, predilection, intrinsic motivation competence) Knowledge and understanding are measured via 5 item s (PA guidelines, cardiorespiratory fitness definition, muscular endurance definition, PA comprehension, improve sport skill) questionnaire Daily behaviour is measured via self-report questionnaire and pedometer step counts	Scores from domains are summed to create a CAPL-2 total score out of 100, which is used to classify the children into one of four interpretative categories (beginning, progressing, achieving or excelling) based on age and sex specific cut points. CAMSA, PACER and Plank are combined within the physical domain, which is worth 30 points. For motivation and confidence 7.5 points are assigned to each three-item component (intrinsic motivation, PA competence, adequacy, predilection) of the assessment, wherein participants respond to bipolar statements “What’s most like me” selecting if it’s “really true for me” or “sort of true for me” (30 points in total). Daily physical activity behaviour as assessed by self-report and daily pedometer step count (30 points) and knowledge and understanding (10 points). The knowledge and understanding component include four questionnaire items and a missing word paragraph activity.
**PFL** *Canada* Lodewyk et al. [58]	Pilot *n* = 860 (9–10) 2013–2014 *n* = 1036 (8–11; NR) 46% (of the 176 that reported sex) approximately 40% completed all measures. 2014–2015 *n* = 1199 (8–12; NR) 44% (of the 327 who reported sex) Paper also reported data for ages 12–19 years old	PFL includes three assessments for each of the four components (active participation, fitness, movement and living skills). Aim of the study was to uncover initial validation evidence	Measures include student profile, living skill questionnaire (feelings—7 items, thinking—7 items, interacting—7 items) Active participation questionnaire (22 items across 3 scales of diversity, interests, intentions) Fitness kills assessed by the Plank Challenge, The Lateral Bound and the Four-Station Circuit Movement skills assessed by the Run-Stop-Return, Throw and Catch with a Bounce, Advanced Kick	Living skill and active participation questionnaires scored on a 4-point Likert scale Fitness and Movement skills assessed by teachers using a 4-point scale (1 = emerging, 2 = developing, 3 = acquired, 4 = accomplished) based on detailed descriptions of each in a rubric provided to teachers
**PLAYfun** *Canada* Cairney et al. [78, 79, 165]	*N* = 215 48% (7–14; NR)	Assess motor competence, comprehension and confidence	18 different movement tasks within five domains that assess different aspects of a child’s movement skills. The five domains are as follows: running locomotor object control—upper body object control—lower body balance, stability, and body control Confidence and comprehension is assessed by rater when child is completing motor competence assessment	Children are assessed using a VAS that is 100 mm in length and divided into four categories: Confidence is rated a low, medium or high Comprehension is rated as *Prompt*: If the child needed the assessor to give them an additional prompt (outside of the instructions) (e.g. “Go on. You can do it.”), or to incite them to perform the skill/task, place a tick in the “Prompt” column. *Mimic*: If the child waited for one of their peers to perform the skill first, place a tick in the “Mimic” column. *Describe*: If the child asked the assessor to describe the skill/task, place a tick in the “Describe” column. *Demo*: If the child asked the assessor to demonstrate the skill/task, place a tick in the “Demo” column.
***Affective domain***				
**AGSYS** *USA* Cumming et al. [81]	*N* = 1675 NR (9–12, NR)	Use the 2x2 achievement goal framework to assess goal approach orientations	12 items related to mastery/ego X approach/avoidance goal framework	5-point Likert scale from 1 (not at all true) to 5 (very true)
**ASK-KIDS** *Australia* Bornholt & Piccolo [82–84]	*N* = 76 43% (4–13.5, 8.1 ± 2.3)	Self-concept in relation to physical movement, natural talent, effort, difficulty, personal identity, and social identity	Dot-point rating scores 1 (low) to 5 (high)	Scores averaged from (1) low to (5) high
**ATCPE** *UK* Jones [85]	*N* = 223 NR (9–12, NR)	Assess attitudes towards curriculum PE	25 items (13 positive and 12 negative)	5-point Likert scale from strongly disagree (1) to strongly agree (5)
**ATOP** *USA* Beyer et al. [86]	*N* = 362 49% (9–13, 11)	Attitudes towards outdoor play	Three scales: Perceived benefit of playing outside 4 items; Extent to which students enjoy unstructured play 3 items; Barriers to outdoor play 5 items	“How much do you agree with each statement?” Responses on a 5-point Likert scale from strongly disagree to strongly agree
**BREQ** *UK* Sebire et al. [87]	*N* = 462 56.9% (7–11; 10.0 ± 0.6)	Assess self-determined motivation for PA and PA psychological need satisfaction in children	Self-determined motivation for PA: 12 items, 3 per motivation scale (intrinsic, extrinsic, external). PA psychological need satisfaction: autonomy (6 items), competence (6 items), relatedness (6 items)	5-point Likert scale from 1 (not true for me) to 5 (very true for me).
**CAPA** *USA* Brustad [88–90]	*N* = 81 53% (9–10; 10.4 ± 0.3)	Measurement of attraction to PA	Original scale has 25 items (5 subscales with 5 items each), shorter scale has 15 items (5 subscales with 3 items each). Subscales include liking of games and sports, liking of physical exertion and exercise, liking of vigorous PA, peer acceptance in sport and games, importance of exercise	Structured alternate. Adapted version used 1 to 4 Likert scale
**CATPA** *USA* Simon & Smoll [87, 91]	*N* = 992 51% (9–12; NR)	Assess attitudes towards PA	6 scales; social, health and fitness, pursuit of vertigo, aesthetic, catharsis, and aesthetic. Each had 8 items	5-point Likert scale and semantic differential technique with a 0-7 bipolar continuum, with 0 as a neutral reference point. Adjectives at each end of the continuum included good-bad, of no use-useful, not pleasant-pleasant, bitter-sweet, nice-awful, happy-sad, dirty-clean, steady-nervous
**CPAS** *USA* DeBate [94]	*N* = 932 100% (9–14, NR)	Physical activity commitment	12 items measuring attitudes and feelings towards PA	Likert scale 0 (strongly disagree) to 3 (strongly agree)
**CY-PSPP** *USA* Welk et al. [95, 96]	*N* = 152 53% (9–11; NR)	Assess physical self-perceptions in children	36 items, 6 items for each of the 6 domains (global self-esteem, physical self-worth, sport competence, body attractiveness, physical strength, physical condition)	4 point structured alternate format and standard 4-point Likert scale for comparison
**DPAPI** *USA* Chen [97]	*N *= 435 51% (11–12; 9.9 ± 1.1)	Assess psychological needs, motivational types, and motivational consequences for PA participation outside of school	Innate psychological needs (6 items), motivational types (12 items), motivational consequences (6 items)	Innate psychological needs, motivational types and motivational behavioural consequences were assessed on a 5-point Likert scale 5 (very like me) to 1 (not like me). Responses to motivational affective consequences included 4 semantic pairs anchored on a 5-point Likert scale with smiley faces
**EnjoyPE** *USA* Shewmake [98]	*N* = 148 47% (8–10; NR)	Assess student’s enjoyment in PE and exergaming	10 statements relating to enjoyment (7) and perceived exertion (3)	5-point Likert scale, strongly disagree (1) strongly agree (5)
**FHC-Q** *USA* Gray et al. [87]	*N* = 112 49.3% (9–12, NR)	Assess energy related behaviours including intake of fruits and vegetables, sugar-sweetened beverages, processed packaged snacks, and fast food; physical activity; recreational screen time; and associated psychosocial determinants	Questionnaire. Utilised Audience Response System through PowerPoint. 71 items in total. Self-determination (9 questions). Outcome expectations (15 questions). Self-efficacy (20) questions. Habit strength (6 questions). Goal intention (6 questions). Knowledge (6 questions). Social desirability (9 questions).	5-point Likert scale
**FAPM** *Australia* Bornholt & Piccolo [83]	*N* = 56 43% (4–11, 8.0 ± 2.1)	Feelings about physical movements	Diagram (stick figures running and catching) researcher reads accompanying paragraph and the child ticks as many words as needed in relation to five general feelings	Responses scaled from 1 (low) to 7 (high)
**HOP’N** *USA* Rosenkranz et al. [101]	*N* = 230 51% (9–10; 9.5 ± 0.7)	Assess psychosocial variables as part of a 3-year randomised controlled trial aiming to prevent obesity through an after-school programme	16 items: PA task self-efficacy (1 item), PA barriers self-efficacy (4 items), PA enjoyment (2 items), Perceived opportunity for PA (2 items), Perceived habitual PA (2 items), and perceived parental support (5 items)	3-point scale (e.g. not sure at all- somewhat sure- very sure). Perceived habitual PA scores were assessed using a 2-item screener, averaged and dichotomised a meeting PA guideline or not. Parental support was rated on a 6-point scale (never to daily)
**LEAP** *Australia* Hyndman et al. [102]	*N* = 197 43% (8–12, NR)	Enjoyment of lunchtime play	Children completed “expected” (before lunch) and “actual” (after lunch) enjoyment of lunch time play using survey cards with pictorial scale	5-point Likert pictorial scale from very unhappy (1) to very happy (5)
**MAAP** *USA* Dunton et al. [103]	*N* = 119 48% (9–13, NR)	Affective and feeling states relate to physical activity	Positive affect, negative affect, physical feeling states all assessed by 2 items each when prompted through a mobile phone	Response options included 0=not at all, 1=a little bit, 2=quite a bit, 3=extremely
**MOSS** *Australia* Weiss et al. [87]	*N* = 155 45% (8–12; 10.2 ± 1.4)	Assess children’s motivational orientation for engagement in PA	27 items, 5 subscales: Challenge (5 items relating to preference for challenging or easy skills), curiosity (4 items relating to desire to participate), mastery (5 items relating to problem solving and mastery attempts), judgement (6 items relating to self-assessment vs teacher assessment), criteria (7 items relating to preference for internal sense of success/failure vs external determined success/failure)	Structured alternative scoring 1 (low) to 4 (high). Children indicate if “Sort of true for me” or “really true for me”. Separate scores given for each subscale. High scores indicate more intrinsic motivation
**NAS** *USA* Nelson et al. [106]	*N* = 382 46% (10–12; 10.8 ± 0.7)	Measure negative attitudes towards PA	All items (8) followed the stem “If I were to be physically active on most days…”	5-point Likert scale from 1 (disagree a lot) to 5 (agree a lot)
**PABM** *USA* Dishman et al. [107]	*N* = 2092 53% (10–12, NR)	Assess motives for physical activity	Self-efficacy (8 items). Perceived barriers: 3 scales; obstacles (3 items), evaluation (3 items), outcomes (3 items). Motives for PA: 30 items, 5 scales for intrinsic; enjoyment (7 items), competence (7 items) and extrinsic; fitness (5 items), appearance (6 items), social (5 items). Parental support (5 items)	All used 4-point order response format apart from perceived parental support, 5 point ordered format. Participants entered all self-administered questionnaire responses into a survey software database on laptop computers
**PACES** *USA* Moore et al. [108]	*N* = 564 53% (8–9; 8.7 ± 0.5	Assess the enjoyment of PA	16 bipolar statements starting with the stem “When I am physically active…”	5-point Likert scale 1 (Disagree a lot) to 5 (Agree a lot)
**PAHFE** *USA* Perry et al. [109]	*N* = 131 54% (8–14; 9.9 ± NR)	Assess personal goal setting and decision-making efficacy for PA and food choices	18 items representing children may experiences when attempting to improve PA and eating behaviours	5-point Likert scale from 1 (not sure at all) to 5 (completely sure)
**PAS** *USA* Nelson et al. [106]	*N* = 382 46% (10–12; 10.8 ± 0.65)	Measure positive attitudes towards PA	All items (8) followed the stem “If I were to be physically active on most days…”	5-point Likert scale from 1 (disagree a lot) to 5 (agree a lot)
**PASE** USA Jago et al. [110]	*N* = 560 49% (NR, 11.3 ± 0.6)	Assess PA self-efficacy	23 physical activity and 24 sedentary behaviour (3 subscales relating to TV, computer/video game/telephone) items were loaded onto palm pilots. All items start with stem “How sure are you that you have (can)…”	Dichotomous options (sure and not sure)
**PASES** *USA* Saunders et al. [87]	*N* = 442 NR (10–11; NR)	Assess psychosocial determinants on children’s PA: social influences, self-efficacy, beliefs, and intention	Social influences (1 factor), self-efficacy (3 factors; support seeking, barriers, positive alternatives), beliefs (2 factors; social outcomes, PA outcomes)	2-point scale (yes or no)
**Physical Activity Self-efficacy, enjoyment, social support** *China* Liang et al. [113]	*N* = 457 50% (8–12, 10.3 ± 1.0)	Assess PA self-efficacy, enjoyment, social support	8 item scale used to measure PA self-efficacy. 7 item scale to assess PA enjoyment. 10 items to assess social support for exercise	Self-efficacy and enjoyment scales used Likert scale ranging from 1 (Disagree a lot) to 5 (Agree a lot). Social support scale used a 5-point scale 1 (none) to 5 (very often)
**PLOC in PE** *USA* Vlachopoulos et al. [114]	*N* = 817 50% (11–12; NR)	Assess the revised PLOC for use in PE	PLOC scale adapted for PE (19 items), perceived autonomy support (6 items), subjective vitality (unclear how many items)	Participants provided their responses on a 1-5 Likert type scale anchored by 1 (totally disagree) 4 (in between) and 7 (totally agree)
**PMCS** *USA* Xiang et al. [115]	*N* = 116 42% (9–10; NR)	Assess perceptions of the motivational climate of team in terms of matter and performance goals	Statement starts with stem “In roadrunners…” followed by 24 items related to perception of motivational climate. 11 mastery focussed and 13 performance focussed items. In original scale (used with older children) 9 mastery and 12 performance items related team…	Participants responded in agreement to statements on a 5-point Likert scales from YES (5) to NO (1) (YES, yes? no, NO), scores calculated by an average for each scale. In original scale 1 (strongly disagree) to 5 (strongly agree)
**RCS** *USA* Lakes & Hoyt [87]	*N* = 112 51% (NR, 4–11)	Assess children’s self-regulatory abilities in physically active context	16 items and three subscales: Cognitive Self-Regulation (6 items, including “control over emotions- uncontrolled emotions”)	Bipolar adjectives (e.g. “attentive–inattentive”) are used for each item, and raters were asked to rate the child using a 7-point scale
**Self-efficacy scale** *USA* Leary et al. [87]	*N* = 15 children 68% (NR, 8.2 ± 0.9)	Assess self-efficacy in overcoming PA barriers	Potentially 12 questions but not reported clearly	5-point Likert scale
**SPPC** *USA* Harter 1982 [87]	*N* = 2704 NR (8–12, NR)	Assess perceived competence in children	36 items, 5 domain specific sub-scale each with 6 items: scholastic competence, social acceptance, athletic competence, physical appearance, behavioural conduct. One global measure of self-worth	Structure alternative format
**TAGM** *Turkey* Agbuga [87]	*N* = 15 57% (8–12, NR)	Trichotomous achievement goal theory in elementary PE	15 items reflecting mastery, performance approach and performance avoidance achievement goals. Each item prefaced “in my PE classes…”	5-point Likert scale (not at all true to very true)
***Physical Domain***				
**ALPHA** *Spain* España-Romero et al. [87]	*N* = 58 NR (6–11; NR)	Fitness assessment	Pubertal status Weight and Height Waist circumference Skinfold thickness (triceps and subscapular) Hand grip strength Standing long jump 4x10m shuttle run test 20m shuttle run test	Individual scores for each test: if the student would not perform the task by selecting a reason: 1=shyness, 2=lack of motivation
**Athletic Skills Track (AST)** *Netherlands* Hoeboer et al. [127]	*N* = 463 NR (6–12; NR)	FMS	The tracks consisted of a series of fundamental motor tasks (n = 10)	Time taken to complete each track
**Bruininks–Oseretsky Test of Motor Proficiency (BOTMP-SF)** *Canada* Fransen et al. [130] [87]	*N* = 2485 47.7% (6–11; 8.5 ± NR)	Motor competence	Consists of 4 motor area composites: fine manual control, manual coordination, body coordination, strength and agility	Motor skills are quantified based on the results of goal-directed activities. A raw score for item outcome may be a drawing, a number of correct activities performed, a number of seconds to complete a task, and/or a complete/incomplete task. A scoring form is used to convert raw scores into point scores
**EUROFIT** *Norway* Cepero et al. [87, 131, 132]	*N* = 119 51% (8–12; 10.4 ± 1.2) Participant numbers reported inconsistently	Fitness assessment	PWC 170 test 6-min run test Arm pull (or hand grip) Standing broad jump (or vertical jump) Bent arm hang Sit-ups in 30 s Sit and reach Plate tapping Shuttle run (10 × 5 m) (or 50 m sprint) Flamingo balance	Highest score for each assessment recorded
**FITNESSGRAM** *USA* Patterson et al. [132–134, 160]	*N* = 84 57% (10–12; NR)	Fitness assessment	PACER, One-Mile Run, Walk Test, Body Fat Percentage (Skinfold and Bioelectrical Impedance Analyser (BIA), Body Mass Index, Curl-Up, Trunk Lift, 90° Push-Up, Modified Pull-Up, Flexed Arm Hang, Flexibility , Back-Saver Sit and Reach, Shoulder Stretch, flexibility and PA behaviour	Individual scores for each assessment then converted to FITnessGram® classifies fitness levels using discrete zones to allow for more personalised feedback
**Golf Swing and Putt skill Assessment** *Australia* Barnett et al. [135]	*N* = 43 NR (6–10; 7.8 ± 1.3)	FMS	Skill Materials Directions Golf Swing Performance Criteria	Scores for both skills were summed for each child resulting in a potential score range of 0-24
**MOBAK-3 test** *Germany* Hermann et al. [87, 140]	*N* = 317 55% (6–7;7.0 ± NR)	Motor skill	10 test items: Throwing/ throwing and catching, bouncing, dribbling, balancing, rolling, rope skipping and moving variably	Test items are dichotomously scaled (0 =failed, 1 = passed, both attempts passed = 2 points)
**Movement assessment battery for children - 2** *Spain* Wagner et al. [87]	*N* = 323 47% (7–10;9.0 ± NR)	Motor skill	The three broad motor skill categories that are assessed are Manual Dexterity, Aiming and Catching, and Balance.	Item performance may be a number of points, a number of performance correct or number of errors performed, and number of seconds to complete task
**MUGI** *Sweden* Ericsson [87]	*N* = 251 NR (6–8; NR)	Motor skill	9 gross motor tasks measuring two components of motor skills; Balance/bilateral coordination Hand eye coordination	Three levels are used for evaluation of motor skills 0, 1 and 2.
**OP** *Croatia* Zuvela [145]	*N* = 95 49% (NR; 8.1 ± 0.3)	Motor skill	Space covering skills Resistance overcoming skills Object control skills	The result of the test is the time needed to successfully accomplish four of the tasks
**PARAGON** *USA* Myers & Wells [87]	*N* = 65 59% (5–9; NR)	Gardening movements	Gardening motions (bending, carrying, lifting, stretching, watering)	For each time interval the observer chooses 1 of the 7 PA codes and 1 of the 9 garden tasks
**SEBT** *Spain* Calatayud et al. [87]	*N* = 24 50% (10–12; 11.0 ± NR)	Balance	N/A	The point at which the participant touched the line was marked by the examiner and measured manually using a measuring tape
**SS** *Australia* Rudd et al. [87]	*N* = 337 NR (6–10; 8.2 ± 1.2) Confirmatory factor analysis: *N* = 300 48% (NR; 8.2 ± 1.1)	Stability skills	Three postural control tasks were selected (the log roll, rock and back support)	Each task completed twice, with tasks broken down into performance components (rock-4, log roll-3, back support 5)
**TGMD-3** *USA* Ulrich et al. [87]	*N* = 1460 50% (5–10; 8.4 ± NR)	FMS	The TGMD-3 assesses 13 fundamental motor skills, subdivided into two subscales: Locomotor: run, gallop, hop, leap, horizontal jump, slide Ball Skills: two-handed strike, stationary dribble, catch, kick, overhand throw, underhand roll	Each skill is evaluated on three to five performance criteria, 2- trials summed per skill 0 = if a criterion was not performed 1 = if a criterion was performed
**Y Balance Test** *USA* Faigenbaum et al. [87]	*N* = 188 NR (6.9–12.1; NR)	Balance	N/A	A total composite score was based on the sum of performance in three directions on both legs
***Cognitive domain***				
**BONES PAS** *USA* Economos et al. [87]	*N* = 41 63% NR, 7.1 ± 0.8)	Assess participation in and knowledge of weight-bearing PA	Children given 10 different PA pictures, and 3 coloured placemats with “yes”, “no”, “I don’t know”; “yesterday”, “the day before yesterday”; “good for building bones”, “not good for building bones”, “don’t know”	Each correct response scored as 1 and all incorrect scores including “don’t know” responses were scored as 0
**PHKA** *Greece* Manios et al. [87]	*N* = 4171 NR (6–10, NR)	Assess knowledge of diet, food products, and PA before and after 3-year intervention	Multiple choice questionnaire	NR

*NR* not reported, *PE* Physical Education, *PA* Physical Activity, *USA* United States of America, *UK* United Kingdom, *CAPL-2* Canadian Assessment of Physical Literacy, *PFL* Passport for Life, *AGSYS* Achievement Goal scale for Youth Sports, *ATCPE* Attitudes Towards Curriculum Physical Education, *ATOP* Attitudes Towards Outdoor play scale, *BREQ* Adapted Behavioural Regulation in Exercise Questionnaire, *CAPA* Children’s Attraction to Physical Activity Questionnaire, *CATPA* Children’s Attitudes Towards Physical Activity, *CPAS* Commitment to Physical Activity Scale, *CY-PSPP* Children and Youth Physical Self-Perception Profile, *DPAPI* Motivational determinants of elementary school students’ participation in physical activity, *EnjoyPE* Enjoyment in Physical Education, *FHC-Q* Food Health and Choices Questionnaire, *FAPM* Feelings About Physical Movement, *FMS* Fundamental Movement Skills, *HOP’N* Healthy Opportunities for Physical Activity and Nutrition Evaluation, *LEAP* Lunchtime Enjoyment of Activity and Play Questionnaire, Momentary Assessment of Affect and Physical feeling states (MAAP);*MOSS* Motivational Orientation in Sport Scale, *NAS* Negative Attitudes Towards Physical Activity Scale, *PABM* Physical Activity Beliefs and Motives, *PACES*,Physical Activity Enjoyment Scale, *PAHFE* Physical activity and Healthy Food Efficacy, *PAS* Positive Attitudes Towards Physical Activity Scale, *PASE* Physical Activity Self-Efficacy Questionnaire, *PASES* Physical Activity Self-Efficacy Scale, *PLOC in PE*,The Revised Perceived Locus of causality in physical Education, *PMCS* Perceived Motivational Climate in Sport Questionnaire, *RCS* Response to Challenge Scale, *SPPC* Self-Perception Profile for Children, *TAGM* Trichotomous Achievement Goal Model, *TEOSQ* Task and Ego Orientation in Sport Questionnaire, *ALPHA* ALPHA Fitness Battery, *AST* Athletic Skills Track ½, *BOTMP-SF* Bruininks–Oseretsky Test of Motor Proficiency, *CAMSA* Canadian Agility and Movement Skills Assessment, EUROFIT, *FG* FITNESSGRAM, *FGCOMP* FG-COMPASS, *GSPA* Golf Swing and Putt skill Assessment, *MOBAK-3* Motorische Basiskompetenzen in der 3, *MABC2* Movement assessment battery for children-2, *MUGI* Motorisk Utveckling som Grund för Inlärning, *OP* Obstacle Polygon, *PARAGON* PA Research and Assessment tool for Garden Observation, *SMT* Slalom Movement Test, *SEBT* Star Excursion Balance Test, *SS* Stability skill test, *TGMD-3* Test of Gross Motor Development-3, *20MSR* The Leger 20m Shuttle Run test, *YBT* Y Balance Test, *BONES PAS* Beat Osteoporosis Now-Physical Activity Survey, *PHKA* Pupil Health Knowledge Assessment

Within the physical domain, assessments were typically administered within the gym hall or an onsite sports facility within the school setting (*n* = 15); only one tool (PARAGON) utilised an outdoor garden setting. Additionally, each physical tool utilised a form of product scoring (i.e. ALPHA, AST, BOT-2 SF, EUROFIT, FITNESSGRAM, MABC-2, MOBAK-3, MUGI, OP, SEBT, YBT), which focuses on the outcomes of the movements (e.g. distance jumped) or process scoring (i.e. GSPA, SS, TGMD-3), which focuses on the technical quality of the movement (e.g. arms extending upwards and outwards during jump). Assessments within the affective and cognitive domain were typically administered via a pen and paper or online questionnaire, with picture/photo support for some. All questionnaires used Likert scale rating systems or structured alternate response formats to score responses. One affective domain assessment, the RCS, consisted of the observation of a child’s completion of a physical activity obstacle course, where observers were asked to score the child’s self-regulation and response to challenge using a 7-point bipolar adjective scale [118]. The two assessments solely included within the cognitive domain were reported in intervention studies [163, 164].

#### Physical literacy Elements

Each tool within the review assessed an element of physical literacy (see Tables 4, 5 and 6). Of the explicit (self-titled) physical literacy tools, PFL assessed 21 out of the 43 elements of physical literacy in our checklist, followed by CAPL-2, which assessed a total of 18 elements, and PLAYfun, which assessed 7 elements. PFL measured the highest elements within the affective domain, assessing 8 out of the 10 identified elements (missing elements: *perceived competence* and *willingness to try new activities*), and within the cognitive domain, assessing 4 elements of the 11 listed (*importance of PA, benefits of PA, ability to describe movement, decision-making*). PFL was the only tool to assess decision-making. CAPL-2 included 11 of the 20 elements identified within the physical domain, the most comprehensive assessment in this regard. CAPL-2 also assessed 4 affective (*confidence, motivation, enjoyment and perceived competence*) and 3 cognitive elements (*importance of PA, effects of PA on the body, benefits of PA*). PLAYfun assessed five elements within the physical domain, one element within the affective domain (*confidence*), and one element within the cognitive domain (*ability to identify and describe movement*).

**Table 4:** An overview of the elements of physical literacy covered by assessments included within the affective domain

Assessment tool	Confidence	Motivation	Emotional regulation	Enjoyment	Persistence/resilience/commitment	Adaptability	Willingness to try new activities	Autonomy	Self-perception/self-esteem	Perceived physical competence
*Explicit physical literacy assessments*										
CAPL-2	•	•		•						•
PLAYfun	•									
PFL	•	•	•	•	•	•		•	•	
*Affective domain*										
AGYS		•			•					
ASK-KIDS					•				•	
ATCPE			•	•					•	•
ATOP Scale			•							
BREQ			•					•		•
CAPA				•					•	
CATPA			•							
CPAS				•		•	•			
CY-PSSP									•	•
DPAPI		•								
EnjoyPE				•						
FHC-Q	•	•								
FAPM			•							
HOP’N	•			•					•	
LEAP				•						
MAAP				•						
MOSS		•			•	•	•			
NAS				•					•	
PABM	•	•		•	•					
PACES				•						
PAHFE	•									
PAS				•					•	
PASE	•							•	•	•
PASES	•					•				•
PLOC in PE		•								
PMSC		•								
RCS			•							
Self-Efficacy Scale	•									
SPPC		•								•
TAGM		•								
TEOSQ		•								

*AGSYS* Achievement Goal scale for Youth Sports, *ATCPE* Attitudes Towards Curriculum Physical Education, *ATOP* Attitudes Towards Outdoor play scale, *BREQ* Adapted Behavioural Regulation in Exercise Questionnaire, *CAPA* Children’s Attraction to Physical Activity Questionnaire, *CATPA* Children’s Attitudes Towards Physical Activity, *CPAS* Commitment to Physical Activity Scale, *CY-PSPP* Children and Youth Physical Self-Perception Profile, *DPAPI* Motivational determinants of elementary school students’ participation in physical activity, *EnjoyPE* Enjoyment in Physical Education, *FHC-Q* Food Health and Choices Questionnaire, *FAPM* Feelings About Physical Movement, *HOP’N* Healthy Opportunities for Physical Activity and Nutrition Evaluation, *LEAP* Lunchtime Enjoyment of Activity and Play Questionnaire, *MAAP* Momentary Assessment of Affect and Physical feeling states, *MOSS* Motivational Orientation in Sport Scale, *NAS* Negative Attitudes Towards Physical Activity Scale, *PABM* Physical Activity Beliefs and Motives, *PACES* Physical Activity Enjoyment Scale, *PAHFE* Physical activity and Healthy Food Efficacy, *PAS* Positive Attitudes Towards Physical Activity Scale, *PASE* Physical Activity Self-Efficacy Questionnaire, *PASES* Physical Activity Self-Efficacy Scale, *PLOC in PE* The Revised Perceived Locus of causality in physical Education, *PMCS* Perceived Motivational Climate in Sport Questionnaire, *RCS* Response to Challenge Scale, *SPPC* Self-Perception Profile for Children, *TAGM* Trichotomous Achievement Goal Model, *TEOSQ* Task and Ego Orientation in Sport Questionnaire, *CAPL-2* Canadian Assessment of Physical Literacy, *PFL* Passport for Life

**Table 5:** An overview of the elements of physical literacy covered by assessments included within the physical domain

Assessment tool	Object control	Stability	Locomotor	Movement skills—land	Movement skills—water	Moving using equipment	Cardiovascular endurance	Muscular endurance	Coordination	Flexibility	Agility	Strength	Reaction Time	Speed	Power	Rhythmic	Aesthetic/expressive	Sequencing	Specific to an environment	Progression (simple-complex)
***Explicit physical literacy assessments***																				
CAPL-2	**•**	**•**	**•**	**•**			**•**	**•**	**•**		**•**	**•**		**•**		**•**				
PLAYfun	**•**	**•**	**•**	**•**					**•**											
PFL	**•**	**•**	**•**	**•**			**•**	**•**	**•**		**•**	**•**								
***Physical domain***																				
ALPHA				**•**			**•**	**•**	**•**		**•**	**•**		**•**						
AST-1/2	**•**	**•**	**•**	**•**					**•**											
BOT-SF	**•**	**•**	**•**	**•**					**•**		**•**	**•**		**•**						
EUROFIT		**•**		**•**			**•**	**•**		**•**		**•**		**•**						
FITNESSGRAM			**•**	**•**			**•**	**•**	**•**	**•**		**•**								
GSPA	**•**			**•**					**•**							**•**				
MABC-2	**•**	**•**		**•**					**•**											
MOBAK-3	**•**	**•**	**•**	**•**		**•**			**•**		**•**	**•**				**•**		**•**		
MUGI	**•**	**•**	**•**	**•**					**•**							**•**				
PARAGON	**•**	**•**		**•**						**•**									**•**	
SEBT		**•**		**•**																
Stability Skills		**•**		**•**																
TGMD-3	**•**		**•**	**•**																
Y-BT		**•**		**•**						**•**										

*ALPHA* ALPHA Fitness Battery, *AST* Athletic Skills Track ½, *BOTMP-SF* Bruininks–Oseretsky Test of Motor Proficiency, EUROFIT, *FG* FITNESSGRAM, *GSPA* Golf Swing and Putt skill Assessment, *MOBAK-3* Motorische Basiskompetenzen in der 3, *MABC2* Movement assessment battery for children-2, *MUGI* Motorisk Utveckling som Grund för Inlärning, *OP* Obstacle Polygon, *PARAGON* PA Research and Assessment tool for Garden Observation, *SEBT* Star Excursion Balance Test, *SS* Stability skill test protocol, *TGMD-3* Test of Gross Motor Development-3, *YBT* Y Balance Test, *CAPL-2* Canadian Assessment of Physical Literacy, *PFL* Passport for Life

**Table 6:** An overview of the elements of physical literacy covered by assessments included within the cognitive domain

Assessment tool	Benefits of PA	Importance of PA	Effects of PA on body	PA opportunities	Sedentary behaviour	Ability to identify and describe movement	Creativity and imagination	Decision-making	Ability to reflect	Tactics, rules and strategy	Safety considerations and risk
**Explicit physical literacy assessments**											
CAPL-2	•	•	•								
PLAYfun						•					
PFL	•	•				•		•			
**Cognitive domain**											
BONES PAS	•	•	•								
PHKA	•										

*BONES PAS* Beat Osteoporosis Now-Physical Activity Survey, *PHKA* Pupil Health Knowledge Assessment, *CAPL-2* Canadian Assessment of Physical Literacy, *PFL* Passport for Life

Within the physical domain, all of the included tools assessed an aspect of movement skills on land; no tool considered movement skills in water. Additionally, fundamental movement skills were well represented, with 53% of tools assessing locomotor skills (AST, BOT-SF, FITNESSGRAM, MABC-2, MOBAK-3, MUGI, OP, TGMD-3), 60% object control skills (AST, BOT-SF, GSPA, MABC-2, MOBAK-3, MUGI, OP, PARAGON, TGMD-3) and 80% of tools reportedly assessed stability skills (AST, BOT-SF, MABC-2, MOBAK-3, MUGI, OP, PARAGON, SEBT, SS, YBT). Few assessment tools explicitly assessed rhythm, speed, aesthetic/expressive movement, sequencing, progression and an application of movement specific to the environment. Within the affective domain, 11 tools related to the assessment of enjoyment (ATCPE, CAPA, CPAS, EnjoyPE, HOP’N, LEAP, MAAP, NAS, PABM, PACES, PAS), making it the most frequently assessed affective element. Nine tools assessed an aspect of motivation (AGYS, DPAPI, FHC-Q, MOSS, PABM, PLOC in PE, SPPC, TAGM, TEOSQ) and seven assessments related to the measurement of confidence (FHC-Q, HOP’N, PABM, PAHFE, PASE, PASES, Self-efficacy scale), while three assessment tools (FHC-Q, PABM) considered both confidence and motivation together within the same assessment. Within the cognitive domain, both BONES PAS and PHKA assessed the benefits of physical activity. No cognitive measures assessed elements related to knowledge and understanding of PA opportunities, sedentary behaviour, creativity/imagination or tactics, rules and strategy.

#### Measurement Properties

Table 7 shows the risk of bias scores (i.e. the methodological quality of the included studies for each measurement property). The data extracted from the studies in relation to validity and reliability can be found in Additional file 4 and Additional file 5, respectively. In general, evidence was limited with few studies reporting across the range of COSMIN measurement properties. Studies reporting the measurement properties of explicit physical literacy assessments tended to have higher methodological quality scores, with all three tools receiving ratings of “adequate” or “very good” for the measurement properties reported. Overall, CAPL-2 was assessed in the most robust methodological studies. CAPL-2 and PFL received quality scores of “very good” for content validity due to studies reporting methods which provided opportunities for experts and child participants to feedback on the assessment such as Delphi consultations and pilot testing. Construct validity was also well reported within research studies for the physical literacy tools, with all three assessments receiving a score of “very good” due to undertaking a confirmatory factor analysis within an adequate sample size and reporting an “acceptable fit” to the data provided. Although all explicit assessments included reliability information, only PLAYfun and PFL reported on internal consistency, while only CAPL-2 had good evidence for test-retest reliability. For PLAYfun, the specific physical subscales scores ranged from poor-to-good for internal consistency (*α* = 0.47–0.82), though only of the subscales was below a good level (< 0.7). For PFL, ICC values ranged from 0.61 to 0.87 across subscales, indicating moderate to good internal consistency. CAPL-2 provided intra-rater reliability results for the plank hold (ICC = 0.83), skill score (ICC = 0.52) and completion time (ICC = 0.99). Inter-rater reliability was good for PLAYfun (ICC, 0.87), and moderate for CAPL-2 in the plank hold (ICC = 0.62) and skill score (ICC = 0.69) though excellent for completion time (ICC = 0.99), though the methodological quality of studies in this regard was only adequate. PLAYfun was the only tool to report information for criterion validity (methodological rigour scored as “very good”), with a moderate to large correlation between PLAYfun and the CAMSA (*r* = 0.47–0.60). CAPL-2 received a score of “very good” for cross-cultural validity, with Dania et al. [76] and Li et al. [77] reporting confirmatory factor analysis procedures that confirm the four-factor structure as a good fit within Greek and Chinese populations, respectively.

**Table 7:** COSMIN risk of bias scores for the methodological quality of the included studies for each measurement property

Assessment tool [studies]	Content validity	Construct validity	Internal consistency	Criterion validity	Cross cultural validity	Test-retest reliability	Intra-rater reliability	Inter-rater reliability
**Explicit physical literacy assessments**								
CAPL-2 [70–77, 80]	VG	VG	–	VG	VG	VG	A	A
PFL [58]	VG	A	D	–	–	A	A	A
PLAYfun [79, 165]	A	VG	VG	VG	–	–	A	A
**Affective domain**								
AGSYS [81]	A	VG	VG	–	–	A	–	–
ASK-KIDS [82–84]	IN	D	VG	–	–	–	–	–
ATCPE [85]	A	–	VG	–	–	–	D	–
ATOP [86]	A	D	VG	–	–	–	–	–
BREQ [87]	IN	VG	VG	–	–	–	–	–
CAPA [87]	A	–	VG	–	VG	–	–	–
CATPA [87]	IN	–	VG	–	–	–	D	–
CPAS [87]	IN	A	–	–	–	–	–	–
CY-PSPP [87]	IN	VG	VG			A	A	A
DPAPI [87]	–	VG	D	–	–	–	–	–
EnjoyPE [87]	A	–	VG	–	–	–	–	–
FHC-Q [87]	A	D	VG	–	–	A	–	–
FAPM [87]	IN	–	VG	–	–	–	–	–
HOP’N [87]	–	–	VG	–	–	–	–	–
LEAP [87]	A	–	VG	–	–	VG	–	–
MAAP [87]	IN	–	VG	–	–	–	–	
MOSS [87]	–	A	VG					
NAS [87]	IN	VG	VG	–	–	–	–	–
PABM [87]	IN	VG	–	–	–	–	–	–
PACES [87]	IN	VG	VG	–	–	–	–	–
PAHFE [87]	A	VG	VG	–	–	IN	–	–
PAS [87]	IN	VG	VG	–	–	–	–	–
PASE [87]	A	A	VG	–	–	–	–	–
PASES [87]	IN	A	VG	–	VG	A	–	–
Physical Activity Self-efficacy, enjoyment, social support [87]	IN	VG	VG	–	–	A	–	–
PLOC in PE [87]	–	VG	VG	–	–	–		
PMCS [87]	A	–	VG	–	–	–		
RCS [87]	D	IN	–	–	–	D	–	D
Self-efficacy scale [87]	VG	–	VG	–	–	–	–	
SPPC [87]	A	VG	VG	–	–	–	–	–
TAGM [87]	IN	IN	VG	–	–	–	–	–
TEOSQ [87]	IN	–	VG	–	–	–	–	–
**Physical domain**								
ALPHA [87]	D	–	–	–	–	D	–	D
AST [87]	A	A	–	VG	–	IN	–	–
BOTMP-SF [87]	A	A	D	D	–	A	–	A
EUROFIT [87]	D	–	–	–	–	D	–	–
FITNESSGRAM [87]	A	–	D	VG	–	A	A	A
GSPA [87]	A	–	–	–	–	D	A	–
MABC-2 [87]	A	VG	VG	VG	A	–	A	A
MOBAK-3 [87]	A	VG	–	–	–	–	–	–
MUGI [87]	–	D	IN	–	–	IN	–	IN
Obstacle Polygon [87]	D	IN	–	D	–	–	IN	–
PARAGON [87]	–	–	–	–	–	D	–	D
SEBT [87]	–	–	–	–	–	A	–	IN
SS [87]	A	VG	–	IN	–	D	–	–
TGMD–3 [87]	A	VG	VG	VG	VG	A	A	A
YBT [87]	–	–	D	–	D	–	–	–
**Cognitive domain**								
BONES PAS [87]	IN	–	–	–	–	IN	–	–
PHKA [87]	–	–	–	–	–	IN	–	–

Canadian Assessment of Physical Literacy; *PFL* Passport for Life, *AGSYS* Achievement Goal scale for Youth Sports, *ATCPE* Attitudes Towards Curriculum Physical Education, *ATOP*,Attitudes Towards Outdoor play scale, *BREQ* Adapted Behavioural Regulation in Exercise Questionnaire, *CAPA* Children’s Attraction to Physical Activity Questionnaire, *CATPA* Children’s Attitudes Towards Physical Activity, *CPAS* Commitment to Physical Activity Scale, *CY-PSPP* Children and Youth Physical Self-Perception Profile, *DPAPI* Motivational determinants of elementary school students’ participation in physical activity, *EnjoyPE* Enjoyment in Physical Education, *FHC-Q* Food Health and Choices Questionnaire, *FAPM* Feelings About Physical Movement, *HOP’N* Healthy Opportunities for Physical Activity and Nutrition Evaluation, *LEAP* Lunchtime Enjoyment of Activity and Play Questionnaire, *MAAP* Momentary Assessment of Affect and Physical feeling states, *MOSS* Motivational Orientation in Sport Scale, *NAS* Negative Attitudes Towards Physical Activity Scale, *PABM* Physical Activity Beliefs and Motives, *PACES* Physical Activity Enjoyment Scale, *PAHFE* Physical activity and Healthy Food Efficacy, *PAS* Positive Attitudes Towards Physical Activity Scale, *PASE* Physical Activity Self-Efficacy Questionnaire, *PASES* Physical Activity Self-Efficacy Scale, *PLOC in PE* The Revised Perceived Locus of causality in physical Education, *PMCS* Perceived Motivational Climate in Sport Questionnaire, *RCS* Response to Challenge Scale, *SPPC* Self-Perception Profile for Children, *TAGM* Trichotomous Achievement Goal Model, *TEOSQ* Task and Ego Orientation in Sport Questionnaire, *ALPHA* ALPHA Fitness Battery, *AST* Athletic Skills Track ½, *BOTMP-SF* Bruininks–Oseretsky Test of Motor Proficiency, *CAMSA* Canadian Agility and Movement Skills Assessment, EUROFIT, *FG* FITNESSGRAM, *FGCOMP* FG-COMPASS, *GSPA* Golf Swing and Putt skill Assessment, *MOBAK-3* Motorische Basiskompetenzen in der 3, *MABC2* Movement assessment battery for children-2, *MUGI* Motorisk Utveckling som Grund för Inlärning, *OP* Obstacle Polygon, *PARAGON* PA Research and Assessment tool for Garden Observation, *SMT* Slalom Movement Test, *SEBT* Star Excursion Balance Test, *SS* Stability skill test, *TGMD-3* Test of Gross Motor Development-3, *20MSR* The Leger 20m Shuttle Run test, *YBT* Y Balance Test, *BONES PAS* Beat Osteoporosis Now-Physical Activity Survey, *PHKA* Pupil Health Knowledge Assessment

Within the affective domain, 87% of included studies provided detail surrounding content validity. This typically included reviews of the literature and contributions from an expert panel. A large number of the affective assessments were originally developed for adolescent or adult populations and were adapted for use with children. As a result, these studies received an “inadequate rating” for content validity. Only 36% of studies involved children in assessment development: ATCPE and CAPA used children to generate items while other studies involved children in pilot assessment or cognitive interviewing (AGYS, ATOP, FHC-Q, MAAP, PACES, PAHFE, PASES, RCS, Self-efficacy Scale, TAGM). The majority of affective related studies reported construct validity (66%), which was commonly determined through confirmatory factor analysis, although the use of other methods and lower sample size downgraded the methodological quality of some of these studies for other tools (CPAS, PASE, PASES, TAGM). The studies of very good methodological quality generally reported that the factor analysis supported the proposed model structure (AGYS, BREQ, CY-PSPP, DPAPI, NAS, PABM, PACES, PAHFE, PAS, PA self-efficacy enjoyment and social support scale, PLOC in PE, SPPC). Cross-cultural validity was reported for CAPA [90] and PASES [112] as both studies provided satisfactory evidence that no important differences were found between language versions in multiple group factor analysis. Only 31% of studies included within the affective domain reported information relating to reliability (AGSYS, ATCPE, CATPA, CY-PSPP, FHC-Q, LEAP, PAHFE, PASES, PA self-efficacy enjoyment social support, RCS). The majority of studies reported internal consistency (91%). With the exception of the DPAPI, all of the tools that did report internal consistency were considered of very good methodological quality as they presented Cronbach’s alpha coefficient for each subscale. The Cronbach’s alpha coefficients generally reported were > 0.7 and therefore deemed acceptable. Only one affective tool was assessed for test-retest reliability within a very good quality study (LEAP). Median kappa agreement scores varied significantly from 0.22 to 0.74 by construct, ranging from fair to substantial agreement [102]. The RCS scored “inadequate” for construct validity, and “doubtful” for inter-rater reliability methodological quality.

Within the physical domain, 13 tools (86%) reported information relating to content validity, however, no assessments received a score of “very good” for methodological quality. Despite the majority of tools utilising “widely recognised or well-justified methods” [61] (i.e. literature reviews, consulting experts, Delphi polls etc.), there was a lack of clarity regarding the implementation of these methods and how/if any findings were analysed. This included information concerning researcher involvement, data collection process, recording of consultations/meetings and who led the analysis of collected information. Nine tools had studies that reported construct validity, with studies of the MABC-2, MOKAB-3, SS and TGMD-3 displaying “very good” methodological rigour and reporting a good fit between each conceptual model and the provided data. In addition, AST, MABC-2 and the TGMD-3 reported “very good” criterion validity protocols. Specifically, moderate correlations were reported between AST and the KTK (*r* = 0.47 to 0.50) and between TGMD-2 and MABC-2 (*r* = 0.30). Internal consistency was reported for 6 assessment tools (BOT-SF, FITNESSGRAM, MACB 2, MUGI, TGMD-3 and YBT) with only the MABC-2 and TGMD-3 receiving scores of “very good” methodological quality due to studies reporting the relevant statistics for each unidimensional scale. MABC-2 showed good reliability across three subscales (*α* = 0.78), alongside the standard scores on each subtest independently (manual dexterity: *α* = 0.77; ball skills: *α* = 0.52; balance: *α* = 0.77). Similarly, the TGMD-3 reported excellent internal consistency: locomotor skills *α* = 0.92; ball skills *α* = 0.89; and object control *α* = 0.92. Finally, the TGMD-3 had very good evidence for cross-cultural validity, with two studies using confirmatory factor analysis to indicate a good factor structure within Spanish and Brazilian populations [151, 154].

Both tools within the cognitive domain, BONES PAS [163] and PHKA [164], were developed as part of a wider intervention. In relation to the content validity of tool development, BONES PAS researchers reported the use of focus groups and literature reviews, while PE specialists were also consulted by the research team to identify common weight-bearing activities that children engage in on a regular basis. The authors noted that the need to quantify knowledge and understanding of weight-bearing physical activity was balanced against the cognitive limitations of children (i.e. short attention span, inability to accurately estimate time). No other details on validity were reported. Both tools (BONES PAS, PHKA) included in the cognitive domain reported test-retest reliability. However, methodological flaws resulted in “inadequate” scoring. BONES PAS was administered by trained research assistants once to each child on the same day, but only 1–2 h apart. PHKA re-administered the questionnaire after a 2-week interval, however, ICC or weighted kappa was not reported. Neither tool within the cognitive domain reported details relating to other measurement properties and therefore these could not be appraised.

#### Feasibility

Table 8 provides the utility matrix ratings of each assessment (maximum score possible=28). All of the explicit physical literacy assessments could be completed using the space and resources available in a typical primary school environment. CAPL-2 (feasibility score=16), PLAYfun (14) and PFL (20) all provide a catalogue of resources online, which can be accessed and used by a class teacher (or any other engaged stakeholder) to prepare for, administer and score all portions of the assessment. PFL, designed for PE teachers, scored highly in qualification requirements, training and participant understanding. PLAYfun is, however, designed to be used by trained professionals (e.g. coach, physiotherapist, athletic therapist, exercise professional or recreation professional) and therefore was deemed less feasible for use by PE teachers in terms of qualifications required, though specific training for the aforementioned professionals is not required. Stearns et al. [79] reported that graduate assistants undertook 3 h of training for PLAYfun, suggesting good feasibility. PLAYfun also records child comprehension; as a result, it scored highly in relation to participant understanding. CAPL-2 scored best for training requirements and time out of the explicit physical literacy assessments. CAPL-2 is reported to be completed in approximately 30–40 min per individual (not including the pedometer assessment of daily PA behaviour across a week), with the knowledge questionnaire taking up to 20 min depending on the child. Teachers are encouraged to conduct the assessment components over separate days if this is more feasible for larger group class sizes. Teachers reported conducting PFL took between 2.5 and 6 classes [58], while four assessors completed PLAYfun assessments with 20 children or less in 3 h, evaluating each child individually in an isolated portion of the gymnasium (remaining students played supervised games and other assessments) [79].

**Table 8:** Feasibility appraisal of each assessment tool

Assessment tool	Time	Space	Equipment	Qualification	Training	Participant understanding	Incomplete assessments
***Explicit physical literacy assessments***							
CAPL-2	***	**	***	**	****	–	**
PFL	**	**	***	****	***	****	**
PLAYfun	**	**	***	*	**	****	–
***Affective domain***							
AGSYS	–	***	****	–	–	****	–
ASK-KIDS	****	****	–	–	–	–	–
ATCPE	****	****	***	–	–	****	–
ATOP	–	****	***	–	–	****	***
BREQ	–	****	****	–	–	–	–
CAPA	***	***	****	*	*	****	–
CATPA	–	****	****	*	–	–	–
CPAS	**	****	****	–	*	–	–
CY-PSPP	–	****	****	–	–	–	–
DPAPI	–	****	****	–	–	–	****
EnjoyPE	****	****	****	–	–	–	–
FHC-Q	**	***	***	–	–	****	–
FAPM	–	***	****	–	–	–	–
HOP’N	–	****	****	**	–	–	–
LEAP	*	****	****	–	–	****	***
MAAP	*	****	*	–	–	–	**
MOSS	***	***	****	–	–	–	–
NAS	–	****	****	*	–	–	–
PABM	–	****	****	*	–	–	–
PACES	**	****	****	*	–	****	****
PAHFE	***	****	****	***	–	****	–
PAS	–	****	****	*	–	–	–
PASE	–	****	***	–	–	****	**
PASES	**	****	****	*	–	****	–
Physical Activity Self-efficacy, enjoyment, social support	–	****	****	–	****	–	–
PLOC in PE	–	****	****	–	–	–	–
PMCS	**	****	****	*	–	–	–
RCS	–	**	**	*	***	–	–
Self-efficacy scale	**	****	****	–	–	****	–
SPPC	–	****	****	*	–	****	–
TAGM	**	****	****	*	–	****	–
TEOSQ	**	****	****	*	–	–	–
***Physical domain***							
ALPHA	**	**	****	**	***	–	–
AST 1-2	****	**	****	**	***	–	–
BOT-2-SF	***	***	*	*	*	–	–
CAMSA	****	**	****	**	***	–	****
EUROFIT	**	*	**	**	***	–	–
FG	*	*	****	**	***	–	**
GSPA	****	*	*	**	*	–	–
MABC-2	**	***	**	**	*	–	–
MOBAK3	***	**	***	**	***	–	–
MUGI	**	**	***	**	**	**	–
OP	****	*	***	**	**	–	–
PARAGON	**	**	*	***	**	–	–
SEBT	****	****	****	**	***	–	–
SS	***	***	***	***	***	–	–
TGMD-3	**	***	****	**	**	–	–
YBT	****	****	****	**	***	–	–
***Cognitive domain***							
BONES PAS	–	****	***	–	–	****	–
PHKA	–	****	***	**	–	–	*

****Excellent, ***good, **fair, *poor, – not reported

*AGSYS* Achievement Goal scale for Youth Sports; *ATCPE* Attitudes Towards Curriculum Physical Education, *ATOP* Attitudes Towards Outdoor play scale, *BREQ* Adapted Behavioural Regulation in Exercise Questionnaire, *CAPA* Children’s Attraction to Physical Activity Questionnaire, *CATPA* Children’s Attitudes Towards Physical Activity, *CPAS* Commitment to Physical Activity Scale, *CY-PSPP* Children and Youth Physical Self-Perception Profile, *DPAPI* Motivational determinants of elementary school students’ participation in physical activity, *EnjoyPE* Enjoyment in Physical Education, *FHC-Q* Food Health and Choices Questionnaire, *FAPM* Feelings About Physical Movement, *HOP’N* Healthy Opportunities for Physical Activity and Nutrition Evaluation, *LEAP* Lunchtime Enjoyment of Activity and Play Questionnaire, *MAAP* Momentary Assessment of Affect and Physical feeling states, *MOSS* Motivational Orientation in Sport Scale, *NAS* Negative Attitudes Towards Physical Activity Scale, *PABM* Physical Activity Beliefs and Motives, *PACES* Physical Activity Enjoyment Scale, *PAHFE* Physical activity and Healthy Food Efficacy, *PAS* Positive Attitudes Towards Physical Activity Scale, *PASE* Physical Activity Self-Efficacy Questionnaire, *PASES* Physical Activity Self-Efficacy Scale, *PLOC in PE* The Revised Perceived Locus of causality in physical Education, *PMCS* Perceived Motivational Climate in Sport Questionnaire, *RCS* Response to Challenge Scale, *SPPC* Self-Perception Profile for Children, *TAGM* Trichotomous Achievement Goal Model, *TEOSQ* Task and Ego Orientation in Sport Questionnaire, *ALPHA* ALPHA Fitness Battery, *AST* Athletic Skills Track ½, *BOTMP-SF* Bruininks–Oseretsky Test of Motor Proficiency, *CAMSA* Canadian Agility and Movement Skills Assessment, EUROFIT, *FG* FITNESSGRAM, *FGCOMP* FG-COMPASS, *GSPA* Golf Swing and Putt skill Assessment, *MOBAK-3* Motorische Basiskompetenzen in der 3, *MABC2* Movement assessment battery for children-2, *MUGI* Motorisk Utveckling som Grund för Inlärning, *OP* Obstacle Polygon, *PARAGON* PA Research and Assessment tool for Garden Observation, *SMT* Slalom Movement Test, *SEBT* Star Excursion Balance Test, *SS* Stability skill test, *TGMD*-3 Test of Gross Motor Development-3, *20MSR* The Leger 20m Shuttle Run test, *YBT* Y Balance Test, *BONES PAS* Beat Osteoporosis Now-Physical Activity Survey, *PHKA* Pupil Health Knowledge Assessment, *CAPL-2* Canadian Assessment of Physical Literacy, *PFL* Passport for Life

Within the affective domain, the highest scoring feasibility tools were PACES (19), PAHFE (18), LEAP (16) and CAPA (16). Within the cognitive domain, BONES PAS scored 11, and PHKA 10, with neither assessment reporting information on time required to complete or training required to administer the questionnaire. Feasibility relating to space and equipment scored highly across the affective and cognitive domain as many of these assessments are pen and paper questionnaires that could be completed in a small space with equipment typically available in a primary school. Studies included within these domains often failed to report further details in relation to feasibility. Only 31% of cognitive and affective assessments had information in relation to the time needed to complete an assessment (ASK-KIDS, ATCPE, CAPA, CPAS, EnjoyPE, FHC-Q, LEAP, MAAP, MOSS, PACES, PAHFE, PASES, PMCS, Self-efficacy scale, TAGM, TEOSQ), 29% of assessments detailed the qualifications of administrators (CAPA, CATPA, HOP’N, NAS, PABM, PACES, PAHFE, PAS, PASES, PMCS, RCS, SPPC, TAGM, TEOSQ, PHKA) and only 8% of assessments had information on the training required to administer these assessments (CAPA, CPAS, Physical Activity Self-efficacy enjoyment social support, RCS). BONES PAS was slightly higher scoring within the cognitive domain, primarily as the assessment scored highly for participant understanding, as children were involved in the development of the scale and statements. Manios et al. [164] reported little detail in relation to feasibility, simply stating the PHKA portion of their data collection “was completed in the presence of a member of the research team”.

Within the physical domain, feasibility scores ranged from 9 (BOTMP-SF) to 17 (YBT, SEBT), with SS (15) also scoring highly. The feasibility findings highlight that typically an appropriate time for a school PE lesson (approximately 50 min) was required to complete an assessment. Specifically, 4 assessments (AST, GSPA, SEBT, YBT) reported taking less than 15 min to complete, with a further 3 tools (BOT-SF, MOKAB-3 and SS) requiring between 15–30 min. Additionally, the equipment needed to conduct assessments was scored positively for the majority of tools, as most required equipment would likely be present in a typical primary school setting, e.g. balls, cones, and skipping ropes. Some tools (40%) did require additional or specialised equipment (OP, GSPA, BOT-SF) such as sport-specific equipment (i.e. junior-sized gold club [GSPA]), or equipment to measure specific elements such as manual dexterity (e.g. pegs and a pegboard [BOT-2 SF]). Furthermore, the majority of assessments required either a PE/Sports specialist/researcher to administer (80%), with only two tools (PARAGON and MUGI) being appraised as “Able to be administered by qualified teacher”.

## Discussion

The aim of this systematic review was to identify and appraise tools to assess physical literacy and related affective, physical and cognitive elements within children aged 7–11.9 years old for use in a primary school PE setting. From 88 studies, a total of 52 unique quantitative assessments were identified and subsequently examined for validity, reliability, feasibility and physical literacy elements being measured. In contrast to Edwards et al. [42], our search did not find any qualitative assessments of physical literacy within this age group. Only three explicit physical literacy assessments were represented in studies that met the inclusion criteria (CAPL, P4L, PLAYFun), though there were a number of assessments within affective (32 assessments) and physical (15 assessments) domains that could be used within a pragmatic physical literacy assessment approach. Far fewer assessments were found within the cognitive domain (two assessments). Our check for assessment of 41 different elements of physical literacy (10 affective, 20 physical and 11 cognitive), contained in various conceptualisations of the concept [1, 20, 26, 67–69], highlighted elements that were consistently measured across tools and those not yet measured through existing assessments. Our analysis revealed that while some tools have established validity and reliability, and are feasible, the quality of reporting in studies concerning many measurement properties are mixed, indicating that more robust methodological work is required to support tool development. Nevertheless, taken together, the results suggest that there are a number of measurement options available to researchers and PE teachers to assess physical literacy and/or its affective, physical and cognitive domains that are feasible for administration within upper primary PE (7–11.9 years old in the UK).

### Study Quality

To be included in this review, studies of quantitative assessments of physical literacy and related domains had to report data for at one least measurement property from the properties assessed using the COSMIN risk of bias checklist. Overall, the methodological quality of studies reporting this information was inconsistent. Studies tended to examine and report on one or two measurement properties (typically an aspect of reliability and/or validity), but rarely addressed all relevant measurement properties within the risk of bias checklist. Reliability was most frequently assessed across all domains, echoing the findings of recent reviews investigating motor skill assessments [167–169]. The majority of studies within the affective domain reported information related to internal consistency (i.e. the interrelatedness of items on a scale) and in the required level of detail (87% of studies receiving a score of “very good”). Similarly, within the cognitive and physical domains, 83% and 80% of assessments provided information relating to tool reliability, respectively. Physical domain assessments were more likely to report inter- and (to a lesser extent) intra-rater reliability due to the assessments being administered and scored by researchers or teachers, whereas cognitive and affective domain assessments typically employed questionnaire methods, and therefore, these reliability dimensions are not relevant. Though test-retest reliability was rarely reported, the wider reporting of a measurement property relating to other aspects of reliability (i.e. internal consistency, intra- and inter-rater reliability) may suggest that, to date, researchers in physical activity, exercise, sport and health fields have prioritised assessing and reporting the reliability of an assessment tool above other measurement properties.

Recent guidance from COSMIN outlines that tool development and content validity are the most important measurement properties to be considered for assessments [61, 62]. We found that 43 tools reported information relating to content validity, however, only 5 tools (TGMD-3, FitnessGram, Self-Efficacy Scale, CAPL-2 and PFL) received a study quality score of “very good”; notably, two of these assessments (CAPL-2 and PFL) were developed specifically as physical literacy tools. This is particularly concerning as if researchers do not provide sufficient evidence that assessments are valid for use within the targeted population, then arguably the assessments are not appropriate for use [61, 62]. COSMIN guidance states that in order to achieve a “very good” score for tool development/content validity, the relevance, comprehensiveness and comprehensibility of assessments should be considered in detail, i.e. “ensuring that included assessment items are relevant and understood by the target population” [61]. This can be achieved by tool developers including participants in the tool development process and encouraging the sharing of experiences and opinions regarding assessment. For tools that received an “inadequate” or “doubtful” score for tool development/content validity, the associated studies failed to provide adequate detail on concept elicitation, i.e. the methods used to identify relevant items and/or how these items were piloted and refined. It is unclear whether this information was not considered by study authors within the tool development process or whether it was just not reported. Our findings around the poor methodological quality of studies reflect those found within recent reviews of motor competence assessments [167, 168]. Taken together, the mixed standards of reporting of information relating to measurement properties indicate that researchers should be encouraged to utilise the COSMIN checklist to improve the methodological quality of assessment development and the reporting of the measurement properties of assessments.

### Explicit Physical Literacy Assessments

There have been significant efforts towards physical literacy in Canada for over a decade [12, 44]. Each of the three explicit physical literacy assessments identified was developed by Canadian organisations who have embraced the concept. These include the Healthy Active Living Research Group’s (HALO) Canadian Assessment of Physical Literacy (CAPL-2: see www.capl-eclp.ca/) [71, 72], Canadian Sport for Life’s Physical Literacy Assessment for Youth (PLAY, specifically PLAYfun, see https://play.physicalliteracy.ca/) [59], and Physical and Health Education Canada’s Passport for Life (PFL, see https://passportforlife.ca/) [58]. These assessments are suitable for ages 8–12 years, 7+ years and 8–18 years, respectively, and supported by a wide range of online resources and training materials, including information and feedback guides for children, parents and teachers. Their stated purposes differ somewhat with CAPL-2 being developed for monitoring and surveillance of physical literacy in children, PFL for formative assessment in PE, and PLAYfun for programme evaluation and research in sport, health and recreation.

We found that CAPL-2 (affective, *n* = 4; physical, *n* = 11; cognitive, *n* = 3) and PFL (affective, *n* = 8; physical, *n* = 9; cognitive, *n* = 4) assessed more physical literacy elements noted within our checklists than the PLAYfun (affective, *n* = 1; physical, *n* = 5; cognitive, *n* = 1) assessment. These tools are anchored within somewhat different evolutions of physical literacy definitions, which may explain the different elements assessed. In 2015, many organisations across sport, health and education sectors in Canada joined together to generate the Canadian Physical Literacy Consensus Statement [7], which endorsed the IPLA/Whitehead definition of physical literacy [7, 21]. As such, CAPL-2 assesses the elements stated within the IPLA definition using a points-based modular system with assessments of motivation and confidence (30 points), physical competence (30 points), knowledge and understanding (10 points), as well as physical activity behaviour (30 points), which can be aggregated to determine a physical literacy score out of 100. The remaining Canadian assessments (PFL, PLAYfun) more closely align with the previous definition put forward by Canadian Sport for Life and PHE Canada in accordance with Whitehead’s earlier work [18]: “Individuals who are physically literate move with competence and confidence in a wide variety of physical activities in multiple environments that benefit the healthy development of the whole person”. PFL has four distinct assessment domains that are intended to be viewed in isolation including movement skills, fitness, living skills (described as feeling and thinking skills), and active participation (diversity, interests and intentions). PLAYfun focuses on assessing movement competence in 18 tasks, respectively. The child’s confidence and comprehension of each movement task can also be simultaneously assessed but are not accounted for in the scoring, indicating a hierarchy of focus on physical competence. PLAY [59] includes a number of other assessment resources including PLAYparent, PLAYcoach, and PLAYself, with the latter being a self-report questionnaire for children that assesses affective and affective elements, but, at the time of this review, no studies were found that reported measurement properties for the wider PLAY tools.

Despite using variations of Whitehead’s conceptualisations of physical literacy, these Canadian explicit physical literacy assessments appear to have distinct assessment hierarchies (i.e. prioritising one domain over another), strong yet different classifications (referring to what is being assessed and what is not, and within fixed chronological age ranges) and diverse scoring criteria [170]. The prioritising of one domain over another within an explicit physical literacy assessment is problematic as it is inconsistent with holistic perspectives that view all domains as equal [48]. Furthermore, while both CAPL-2 and PFL assess across affective, physical and cognitive elements of physical literacy, these are modular assessments, and thus, domains are assessed in isolation, reflective of more pragmatic approaches to physical literacy assessment [42]. Each tool uses self-reported questionnaires to capture affective, cognitive or behavioural domains of physical literacy, thus allowing the participant to portray their own capabilities. Yet assessments within the physical domain are primarily framed as teacher-led and assessed through process and product criteria interpreted against age and sex-specific norms (CAPL-2), or detailed rubrics (PFL) and rating systems (PLAYfun) based on the quality of movement [170]. The latter provide a more individualised focus for the assessment and reduce comparisons with others, which some may consider more reflective of agreed conceptualisations of physical literacy [48]. PFL and PLAYfun tools show promise in capturing important aspects of physical literacy, but more validity, reliability and feasibility evidence are required. CAPL-2 demonstrated the strongest methodological quality of the three explicit physical literacy assessments, with good validity and reliability reported across several studies. Furthermore, CAPL-2 is the only one of the three tools that has provided evidence of cross-cultural validity, supporting its potential use with other countries and cultures [76, 77]. Accordingly, to date, we suggest that the CAPL-2 is currently the most robust explicit physical literacy assessment tool available to PE teachers and researchers to assess children aged 8 to 12. Of course, each explicit physical literacy assessment can be aimed at different purposes, so practitioners are encouraged to reflect on the most appropriate tool that fits their needs [54].

### Assessments of the Affective Domain

The affective domain of physical literacy includes elements such as confidence, motivation, emotional regulation and resilience [1, 20, 26, 67–69]. In total, we found 32 assessments within this domain (35 including CAPL-2, PFL and PLAYfun), with enjoyment being the most frequently assessed affective element (13 assessments), followed by motivation (11 assessments), confidence (10 assessments) and perceived competence (8 assessments). Enjoyment is not explicitly included in definitions of physical literacy [2], though Edwards et al. [1] did identify “*engage, enthuse, enjoy*” as a core category of physical literacy and “*engagement and enjoyment*” is listed as an element within the psychological domain of the Australian Physical Literacy Framework [16]. Previous research has linked enjoyment to intrinsic motivation and more autonomously regulated behaviour in relation to PE and PA [11, 171, 172], as well as meaningful experiences in PE [173]. The importance of enjoyment indicates that researchers and PE teachers may wish to consider the construct within a physical literacy assessment approach within PE. Further research and consensus are needed, however, on whether enjoyment should be a more prominent (i.e. core) element of physical literacy due to its relevance in fostering meaningful movement experiences—perhaps likened to the ongoing considerations concerning the inclusion of social and behavioural elements in relation to physical literacy [6, 17, 28].

Considering the explicit physical literacy assessment tools, PLAYfun records two affective elements (confidence and willingness to try new things), yet these do not contribute to the PLAYfun scoring (*NB*. PLAYself [59] does assess wider affective items, but no studies reporting measurement properties were located at the time of this review). CAPL-2 includes questionnaire items stated to assess confidence, intrinsic motivation, enjoyment, and perceived physical competence, though the confidence items more closely relate to perceived competence (e.g. “When it comes to playing active games, I think I’m pretty good”) and adequacy (e.g. “Some kids are good at active games, Other kids find active games hard to play”), than confidence or self-efficacy per se, which corresponds with capability beliefs about whether the movement or physical activity behaviour can be achieved [174, 175]. The PFL questionnaire items assessed eight elements of the affective domain and therefore was the most comprehensive; the only element it did not assess was the willingness to try new activities. As a result, and in consideration of the reported measurement quality, properties and feasibility, this could be an appropriate questionnaire-based method to assess the affective domain of physical literacy in this age group (7–11.9 years), though this questionnaire is lengthy (21 items) and would take longer for children to complete.

We identified 32 other tools that assessed affective related elements of physical literacy and could therefore be useful in a physical literacy measurement approach. Several of these tools reported good evidence for construct validity and internal consistency (AGSYS, BREQ, CY-PSPP, NAS, PASES, PAHFE, PAS, PASSEESS, PLOC in PE, SPPC), indicating that they were theoretically sound in their measured outcomes. Eight of these additional tools measured at least three affective elements in our checklist (ATCPE, BREQ, CPAS, HOP’N, MOSS, PABM, PASE, PASES). For example, the PABM (motivation, confidence and enjoyment, persistence), ATCPE (emotional regulation, enjoyment, self-esteem, perceived physical competence) and PASE (confidence, autonomy, self-esteem and perceived physical competence) each include items to assess four affective elements. There were 13 tools that only assessed one element: ATOP (emotional regulation), DPAPI (motivation), EnjoyPE (enjoyment), FAPM (emotional regulation), LEAP (enjoyment), MAAP (enjoyment), PAHFE (confidence), PLOC in PE (motivation), PMSC (motivation), RCS (emotional regulation), Self-efficacy scale (confidence), TAGM (motivation) and TEOSQ (motivation). While many affective measures were found, these individual elements are frequently assessed as multi-dimensional constructs and as such include a large number of questions/items per attribute. Thus, regardless of their feasibility, methodological quality and measurement properties, these tools only provide a narrow picture of the affective domain of physical literacy and would therefore need to be combined with other affective assessments if a more comprehensive assessment was sought by PE teachers or researchers.

The majority of the affective (and cognitive) assessments included within this review were questionnaire based. The systematic review by Edwards et al. [42] on physical literacy measurement identified a number of qualitative assessments including interviews, reflective diaries, and participant observation used amongst children under 12. These findings suggest that alternative methods are available, though these studies were not identified in the current review using our search terms and inclusion criteria. Although these qualitative assessment methods can be individualised, ipsative, holistic and thus aligning with idealist perspectives of physical literacy [48], these methods are perhaps not appropriate to effectively assess the affective/cognitive domains of physical literacy in children when used in isolation due to the (in)stability of children’s thoughts and feelings [42]. Thus, regular observations of children would be important to chart progress in relation to an individual’s attitudes, beliefs, emotions and understanding in relation to movement and physical activity. Yet the feasibility of time-poor primary school PE teachers undertaking these qualitative assessments with a class of approximately 30 children is unclear. Thus, more research is needed to develop rigorous qualitative methods that align with the stated definition adopted for physical literacy and its corresponding elements and are feasible for use in school contexts by primary school teachers.

### Assessments of the Physical Domain

Physical competence is a fundamental component of physical literacy and as such is represented in every contemporary definition of the concept available [2, 42]. Within the physical domain, there is some overlap between physical competence and common terminology used within well-established research fields, i.e. motor competence, motor control, motor proficiency, and health- and skill-related fitness [13–15]. This was further supported by the findings of this review as a high proportion of existing tools assessed fundamental movement skills (AST, BOT-2 SF, MABC-2, MOBAK-3, MUGI, OP, TGMD-3) and fitness components (ALPHA, EUROFIT, FITNESSGRAM). Similar to recent reviews on motor competence assessments [167, 168], we found that the TGMD-3 [149–155, 162] and MABC-2 [136–139] had the best methodological quality studies for measurement properties of the movement skill-specific assessments, while FITNESSGRAM [132–134, 160] had the best methodological quality studies for the broader health and skill-related fitness test batteries. All tools within the physical domain provided assessments for land-based movement skills, though we did not examine whether assessments were suitable for assessing the use of such skills within different terrains (e.g. rocky-terrain, forest, sand). None of the tools assessed water-based activities, despite swimming being the only compulsory physical activity within the UK, Australian and American primary PE curriculums [37, 176]. Similarly, through our search terms and inclusion criteria, we did not identify any assessments of cycling, which is an important foundational movement for physical activity across the lifespan [177], nor did we identify tools designed to explicitly assess the elements of aesthetic/expressive movement, sequencing, progression and application of movement specific to the environment. This could be a limitation of our search strand (e.g. we did not include dance as a search term, but did include “coordination” and “performance”) or a consequence of the lack of assessments of these elements in this age group and/or associated studies not reporting information on measurement properties to meet the inclusion criteria. Given that the capability to move within different environments, regardless of weather, season, or terrain, will likely influence a child’s safety and opportunities to be physically active, the appropriateness of land-based assessments to assess competence in moving across different terrains warrants further study. Similarly, the identification and appraisal or development of assessments of dance and foundational movement skills for lifelong physical activity such as cycling, and swimming should be a focus for future research.

Of the self-titled physical literacy assessments, CAPL-2 explicitly assessed 11 elements within the physical domain—the most comprehensive assessment in this regard, PFL 9 elements, while PLAYfun assessed 5 elements. PLAYfun only assessed skill-related aspects of physical competence and did not include any measures of strength or endurance, which have been found to be important markers of health and functional living across the life course [178–180]. The assessments within the physical domain utilised a form of product scoring (i.e. ALPHA, AST, BOT-2 SF, EUROFIT, FITNESSGRAM, MABC-2, MOBAK-3, MUGI, OP, SEBT, YBT), which focuses on the outcomes of the movements (e.g. distance jumped, time to completion) or process scoring (i.e. GSPA, SS, TGMD-3), which focuses on the technical quality of the movement (e.g. arms extending upwards and outwards during jump). Some researchers have argued that the use of product-based scoring does not consider the quality of the movement and therefore potentially provides an opportunity for children to draw comparisons between peers, which they consider problematic as physical literacy is a concept concerned with the unique individual [42, 48]. On the other hand, researchers advocating for nonlinear perspectives on movement competence argue that assessing the technical quality of movement is less important than the functional effectiveness of the movement, which can be achieved through a range of different movement solutions [181]. Moreover, product scoring does require less training and expertise than observing the quality of movement [182, 183], and so therefore may have a place in primary school assessment providing it is administered in an appropriate, non-competitive manner.

### Assessments of the Cognitive Domain

For individuals to value and take responsibility for maintaining an active lifestyle, knowledge and understanding of the benefits of involvement in physical activity and of the nature of different activities and their particular challenges is important [20, 184, 185]. The cognitive domain checklist therefore included 11 elements related to the knowledge and understanding of factors related to physical activity [1, 20, 26, 67–69]. We found two assessments that solely related to elements within the cognitive domain of physical literacy (BONES PAS, PHKA), though the methodological quality of these studies [163, 164] was inadequate and therefore we do not recommend these tools for use at this time. Some cognitive aspects are also captured in the explicit physical literacy assessments (CAPL-2, PFL and PLAYfun). BONES PAS, PHKA, CPAL-2 and PFL included an assessment for *knowledge and understanding of the benefits of PA*, an element which is associated with improved PA behaviours [185] and a defining element within Whitehead’s interpretation of the cognitive domain [21]. BONES PAS, CAPL-2 and PFL also assessed the *importance of PA*, while BONES PAS and CAPL-2 both assessed the *effects of PA on the body*. Considering these five tools together in relation to the cognitive domain, there remains a lack of assessments relating to the sub-elements of *sedentary behaviour*, *safety considerations*, *reflection*, *creativity and imagination in application of movement,* and *knowledge and understanding of tactics, rules and strategy*. The original CAPL assessment [67] did include items related to safety, activity preferences, and screen time guidelines, but they were removed from CAPL-2 following a Delphi survey with experts and because of their weak factor loadings onto higher order constructs [73]. Movement creativity is a perceptual ability that requires emotional regulation and critical thinking, with a high degree of knowledge and understanding required to achieve a task goal [186, 187]. Assessing movement creativity could be an important outcome for PE teachers within a physical literacy assessment approach as children that can create and modify movement actions within different physical activity environments can also identify opportunities to engage in physical activity [188]. Furthermore, knowledge of tactics, rules and strategy are likely to be important outcomes for the primary educational curriculum wherein children are introduced to competitive games and sports and asked to apply basic principles of attacking and defending [189]. Thus, working with PE educators to establish assessments in this regard would be useful to chart developmental progress in cognitive domains of physical literacy.

The cognitive domain is the least frequently assessed domain of physical literacy in children aged 7–11.9 years old, and the least represented domain in the explicit physical literacy assessments. This is problematic for holistic considerations of physical literacy. Identifying stage-appropriate knowledge and understanding in relation to physical activity, and the subsequent assessment of this competency, and its relationship to physical activity behaviour, is an area for ongoing development. The development of the Physical Literacy Knowledge Questionnaire for children aged 8–12 years old in CAPL-2 by Longmuir et al. [73] followed robust methodological work. This included content analysis of the educational curriculum, contributions from expert advisors and the piloting of open-ended questions with children, to generate the closed-ended format. Again, it may be beneficial for physical literacy researchers to examine educational curriculums and explore other fields such as physical activity or health literacy, to identify what is stage-appropriate knowledge in this age group, and how this is assessed. Health literacy, defined as the ability of an individual to find, understand, appraise, remember and apply information to promote and maintain good health and wellbeing [190–192], includes similar core outcomes to physical literacy. Therefore, the potential links between health and physical literacy warrant further study [193]. Taken together, the cognitive domain is understudied and perhaps not widely understood. Therefore, more research is needed to identify and clarify the key cognitive elements that are important to the concept of physical literacy and enrich assessments of this domain.

### Feasibility

Teachers have noted significant barriers to implementing assessment in PE [34, 35, 40. 46-48] [194]. Therefore, considering the feasibility of each physical literacy assessment tool in relation to a primary school context was an important aspect of this review. The results of this review suggest that many of the included assessments could be suitable for a primary school setting. The explicit physical literacy assessments (CAPL-2, PLAYfun, PFL) scored relatively high for feasibility, though PLAYfun required more qualified staff to administer the tool, suggesting that this tool may not be feasible for a generalist teacher. These explicit tools generally scored higher as a result of more comprehensive reporting of feasibility information within studies. This is likely because they have been designed with practitioners in mind, reflecting a growing demand for assessments within applied rather than research or clinical settings [66]. Both CAPL-2 and PFL assess affective, physical and cognitive elements of physical literacy but the assessment process can be lengthy in terms of time, with the assessment of large groups of children necessitating assessment activity to run across several classes. This indicates the feasibility challenges of using separate domain-level assessments of physical literacy to paint an overall “holistic” picture of a child’s physical literacy.

Klingberg et al. [66] conducted a systematic review of the feasibility of motor skill assessments for preschool children and their findings revealed weak reporting of feasibility-related information. Similarly, we found that the quality of reporting of some aspects of feasibility information was lacking for many assessments. For example, a large number of affective and cognitive domain assessments did not report information on the training and qualifications required to administer and score the assessment, nor the time it would take for children to complete the assessment (see Table 8). Furthermore, across domains, only around a third of tools reported information on participant understanding of the assessments, which is particularly important if an assessment is to be used as assessment for learning, as feedback is a crucial part of the assessment process [195]. Affective and cognitive assessments were mostly questionnaires and therefore scored excellent for space and equipment required. Some of the physical assessments scored poorly for space requirements due to needing over 20 m of space for some aerobic or locomotor tasks (e.g. 20-m shuttle run in EUROFIT), which would not be possible indoors in a primary school within a UK context. Studies associated with assessment tools within the physical domain better reported the training and qualification skills required to administer assessments, though most tools rated as “fair” as they generally needed to be conducted by a PE/ sports specialist, or a researcher with additional qualifications. Typically, physical domain assessments using product-based scoring which focuses on quantifying the outcome of the movement (e.g. EUROFIT, MOBAK) scored slightly higher for feasibility in terms of expertise required than assessments that assessed the technical quality of the movement (e.g. TGMD-3). Although not included within the matrix, the equipment costs of many of the assessments should not be a barrier to assessment and could easily be met within primary school budgets. Many of the assessments are freely available, while the cost of the resources for physical assessments, which require sports equipment, is typically under $1000 (e.g. full equipment kits for MABC2 $976, TGMD-3 $300, YBT $260, respectively).

Feasibility findings suggest that there is insufficient attention given to reporting the expertise, confidence and competence of individuals required to administer assessments, particularly in assessments within the affective and cognitive domains. Therefore, an effective assessment would need to consider who would be conducting it to determine any potential training needed, ultimately, this would be an influential factor in the overall cost of the assessment. Edwards et al. [42, 53] and Goss et al. [194] highlighted the need to support teachers with continuous professional development in order to ensure that pedagogical processes regarding assessment, teaching and learning were appropriate. Thus, assessments aimed towards educators should ensure that appropriate training and resources, designed at a level to be understood by generalist primary school teachers, should be offered. This could include written guidance for how to administer questionnaires, model videos of how to score physical competence assessments [52, 194], and the creation of communities of practice to support the ongoing development of physical literacy assessment. While it may require additional resources to effectively prepare classroom teachers to administer assessments, enabling the teacher to conduct and interpret the results of a physical literacy assessment is particularly important as a classroom teacher will relate to and understand their pupils on a deeper level than that of a researcher [46].

### Future Considerations in Physical Literacy Assessment

Goss et al. [194] recently examined stakeholder perceptions of physical literacy assessment in a qualitative study involving children, teachers, academics and practitioners. In the study, children themselves highlighted that assessment should be a fun and enjoyable experience. Participants across stakeholder groups indicated that being active, working with peers, providing optimal challenges, and positive teacher feedback would contribute to a fun assessment. Scholars have also argued that assessment in PE should be an enjoyable and motivating learning experience [195, 196], particularly given, as noted above, the importance of enjoyment for autonomous motivation and meaningful experiences in PE [171–173]. Therefore, whatever measure/assessment is used, researchers and practitioners should monitor children’s acceptability, satisfaction, and enjoyment of the assessment process. This is important as poor experiences of assessment could generate negative memories of PE, which could have implications for lifelong enjoyment and motivation for physical activity [197, 198]. This review has identified a range of assessments of learning within physical literacy and related domains, yet it is unclear how these assessments help to support children’s learning per se. Learning is a critical concept within physical literacy [1, 15, 20, 21, 26] and many teachers and educators would argue that assessment should be a learning experience [194–196]. Future research should therefore explore the learning potential of physical literacy assessments, for example in developing children’s knowledge and understanding of movement and physical activity concepts. Moreover, researchers could evidence how an assessment helps children to chart and reflect on their own physical literacy journey, setting goals and optimal, realistic challenges [48]. In relation, more evidence is needed concerning if and how results from physical literacy assessments are returned to learners, as well as if and how learners utilise this feedback. In order for an assessment to inspire learning and have educational impact, participants should feel empowered [195, 199]. To achieve this, physical literacy assessment results could be discussed by teachers/researchers with each individual child and their parents, with constructive and encouraging feedback offered in terms of areas where the child is progressing well on their physical literacy journey and areas for development [39, 194, 195, 200, 201]. Therefore, assessment developers and manuals should include guidance on how to facilitate a meaningful discussion concerning progress with individual learners and key stakeholders. Future researchers could examine the subsequent implementation and effectiveness of these feedback guidelines by the assessment users.

Our findings suggest that there is scope for more research developing and examining rigorous qualitative methods of physical literacy assessment for use in primary school contexts. Such methods might include interviews, verbal discussions, pupil diaries, portfolios, photographs, video, text, drawing tasks and storytelling [42, 48, 202]. Given teacher time constraints [51, 52], future studies could also explore the development of self-assessment and reflective strategies and the use of technology [194]. Self-assessment aligns with the person-centred philosophy of physical literacy [48] and has been found to promote self-regulated learning and self-efficacy [203]. Self-assessment could also provide an opportunity for children to evaluate and reflect on their progress and help to develop their self-awareness of meaningful experiences [202]; in turn, empowering children to take ownership of their relationship with physical activity [48, 202]. Few of the assessments identified within our review utilised technology. Nevertheless, the importance and use of technology in PE assessment were highlighted within a recent position statement from the International Association for Physical Education in Higher Education (AIESEP) [204]. Technology has been successful within an assessment for the learning process that enhanced knowledge and understanding [205] and has been shown to provide an engaging and learning experience for students of all abilities [206]. Furthermore, technology can be used to support students to document their learning experiences and physical literacy journey through pictures and videos, which can be uploaded to mobile and web-based platforms and shared for discussion with wider stakeholders, including teachers and parents [52]. Thus, further research examining how technology can be used to support physical literacy assessment in PE is warranted.

### Strengths and Limitations

The strengths of this systematic review include:

(i) The use of wider search terms encompassing physical literacy elements identified 52 physical literacy or related affective, physical and cognitive assessments that can be used to inform assessment approaches in PE.
(ii) An assessment of the methodological quality of included studies through the COSMIN risk of bias checklist enabled a robust, transparent and systematic appraisal of the validity and reliability standards of the identified quantitative assessments.
(iii) The reporting of the feasibility of assessments provided pragmatic information that can be used by teachers, coaches and researchers to decide whether a tool is appropriate for use in PE and educational contexts.

The limitations of this systematic review include:

(i) Only papers published in the English language were considered. Thus, the identified assessment tools were primarily derived from the US, the UK, Australia, Canada and Western Europe and relevant assessments developed within non-English language countries may have been missed.
(ii) To be included in the review, articles had to be published in a peer-reviewed journal and written in the English language. Therefore, tools developed by practitioners and used currently within schools may not have been captured.
(iii) Although we used “assessment” related search terms in our search strand, we did not capture any qualitative assessments of physical literacy. Had we used more specific qualitative methods as search terms (e.g. interviews, focus groups) then we might have captured more assessments better aligned with an idealist perception of assessment of physical literacy.
(iv) The developed search strand did not include sport-specific search terms such as, “*swimming*”, “*dance*” and “*gymnastics*”. Inclusion of these terms may have better captured water-based assessments and tools assessing elements such as rhythm, coordination and expressive/aesthetic movement.
(v) The physical literacy elements checklist reflects commonly identified elements and was developed by the research team through discussion in a closed meeting after an overview of international physical literacy literature was conducted [1, 20, 26, 67–69]. Some elements identified within international definitions and various conceptualisations of the concept were not included in our checklist and therefore not checked for, but this should not diminish their respective importance. In addition, assessments of elements were categorised within physical, affective and cognitive domains in accordance with different definitions and conceptualisations of physical literacy in order to position assessments into familiar categories for assessment users [1, 2, 6, 16, 20, 26]. Arguably, many physical literacy elements and therefore assessments could span across different domains. For example, confidence is commonly classified within the affective domain within physical literacy conceptualisations, but confidence could also be classified within the cognitive domain as it is influenced by social-cognitive means [207]. Consequently, our checklist should not be taken as the definitive list of key elements within the concept. Researchers should check and appraise the tools for the elements in accordance with their stated definition of physical literacy.
(vi) Each assessment tool was appraised for physical literacy elements in accordance with the explicit information provided within the associated studies and manuals. It is therefore possible that some tools may assess wider elements than those appraised within our results and this should be explored in future research.

## Conclusions

There is demand amongst primary school children and wider stakeholders in England for assessments to chart progress in physical literacy [194]. This systematic review has identified three explicit physical literacy assessments and a number of assessments within affective and physical domains that could be used within a pragmatic physical literacy assessment approach. The review provides information that can help researchers and PE teachers understand what elements of physical literacy are being assessed and what elements are being missed. Our findings highlight that the methodological quality and reporting of measurement properties in the assessment literature require improvement. Furthermore, while many assessments are considered feasible within a school context, further empirical research is needed to consider the feasibility of the scoring and administration of assessment tools by teachers as opposed to researchers. Nevertheless, this review provides information that can be used by researchers and PE teachers to inform the selection or development of tools for the assessment of physical literacy within the 7–11.9-year-old age range.

## Supplementary Information

**Additional file 1.**

**Additional file 2.**

**Additional file 3.**

**Additional file 4.**

**Additional file 5.**

## Data Availability

The datasets used and/or analysed during the current study are available from the corresponding author on reasonable request.

## Abbreviations

PA: Physical activity
COSMIN: COnsensus-based Standards for the selection of health status Measurement INstruments
PRISMA: Preferred Reporting Items for Systematic review and Meta-Analysis
IPLA: International Physical Literacy Association
PROM: Patient-Reported Outcome Measures
AGSYS: Achievement Goal scale for Youth Sports
ATCPE: Attitudes Towards Curriculum Physical Education
ATOP: Attitudes Towards Outdoor play scale
BREQ: Adapted Behavioural Regulation in Exercise Questionnaire
CAPA: Children’s Attraction to Physical Activity Questionnaire
CATPA: Children’s Attitudes Towards Physical Activity
CPAS: Commitment to Physical Activity Scale
CY-PSPP: Children and Youth Physical Self-Perception Profile
DPAPI: Motivational determinants of elementary school students’ participation in physical activity
EnjoyPE: Enjoyment in Physical Education
FHC-Q: Food, Health and Choices Questionnaire
FAPM: Feelings About Physical Movement
HOP’N: Healthy Opportunities for Physical Activity and Nutrition Evaluation
LEAP: Lunchtime Enjoyment of Activity and Play Questionnaire
MAAP: Momentary Assessment of Affect and Physical feeling states
MOSS: Motivational Orientation in Sport Scale
NAS: Negative Attitudes Towards Physical Activity Scale
PABM: Physical Activity Beliefs and Motives
PACES: Physical Activity Enjoyment Scale
PAHFE: Physical activity and Healthy Food Efficacy
PAS: Positive Attitudes Towards Physical Activity Scale
PASE: Physical Activity Self-Efficacy Questionnaire
PASES: Physical Activity Self-Efficacy Scale
PLOC in PE: The Revised Perceived Locus of causality in physical Education
PMCS: Perceived Motivational Climate in Sport Questionnaire
RCS: Response to Challenge Scale
SPPC: Self-Perception Profile for Children
TAGM: Trichotomous Achievement Goal Model
TEOSQ: Task and Ego Orientation in Sport Questionnaire
ALPHA: ALPHA Fitness Battery
AST: Athletic Skills Track ½
BOTMP-SF: Bruininks–Oseretsky Test of Motor Proficiency
CAMSA: Canadian Agility and Movement Skills Assessment
EUROFIT; FG: FITNESSGRAM
FGCOMP: FG-COMPASS
GSPA: Golf Swing and Putt skill Assessment
MOBAK-3: Motorische Basiskompetenzen in der 3
MABC2: Movement assessment battery for children-2
MUGI: Motorisk Utveckling som Grund för Inlärning
OP: Obstacle Polygon
PARAGON: PA Research and Assessment tool for Garden Observation
SMT: Slalom Movement Test
SEBT: Star Excursion Balance Test
SS: Stability skill test
TGMD-3: Test of Gross Motor Development-3
20MSR: The Leger 20m Shuttle Run test
YBT: Y Balance Test
BONES PAS: Beat Osteoporosis Now-Physical Activity Survey
PHKA: Pupil Health Knowledge Assessment
CAPL-2: Canadian Assessment of Physical Literacy
PFL: Passport for Life
