# Supplementary material for: Assessments Related to the Physical, Affective and Cognitive Domains of Physical Literacy Amongst Children Aged 7–11.9 Years: A Systematic Review
Source: Sports Med Open. 2021 May 27;7:37. doi: 10.1186/s40798-021-00324-8 (PMC8160065; doi:10.1186/s40798-021-00324-8)
Supplement: Supplementary file 1 — Additional file 1. [file 40798_2021_324_MOESM1_ESM.pdf]

ONLINE RESOURCE 1 – PICOS information

Assessments related to the physical, affective and cognitive domains of physical literacy among children aged 7-11.9 years: a systematic review

[redacted]

PICOS information

	Include	Exclude
**Population**	Typically developing children<br>Age 3-11	Is not used with children<br>Special Populations
**Intervention**	Studies will be included if they report an Assessment OR Measurement OR Test OR Tool OR Instrument OR Battery OR Method OR Psychometric OR Observation OR Indicator OR Evaluation OR Validity Or Reliability	
**Context**	Physical literacy, physical activity, play, sport, physical education, exercise, recreation<br>Field Based assessment	Lab based
**Outcomes**	Assessment of outcome(s) related to physical literacy.<br>Motivation OR Enjoyment OR Confidence OR Self Or "Perceived Competence" OR Affective OR Social OR Emotion OR Attitude OR Belief OR Physical OR Fitness OR Motor OR Movement OR Skills OR Technique OR Mastery OR Ability OR Coordination OR Performance OR "Perceptual Motor" OR Knowledge OR Understanding OR Value OR Cognitive OR Health OR Wellbeing	
**Study design/ publication characteristics**	Peer reviewed journal articles published in English	Studies published in a foreign language<br>Not published in a peer reviewed journal<br>Duplicate publication<br>They did not assess the psychometric properties of the relevant instrument<br>Full text articles were not available,<br>Studies that dealt with translated and culturally adapted versions of the measures<br>Book chapters, case studies, student dissertations, conference abstracts, review articles, meta-analyses and editorials , protocol papers, systematic reviews<br>Named and used in multiple studies
