# Supplementary material for: Assessments Related to the Physical, Affective and Cognitive Domains of Physical Literacy Amongst Children Aged 7–11.9 Years: A Systematic Review
Source: Sports Med Open. 2021 May 27;7:37. doi: 10.1186/s40798-021-00324-8 (PMC8160065; doi:10.1186/s40798-021-00324-8)
Supplement: Supplementary file 2 — Additional file 2. [file 40798_2021_324_MOESM2_ESM.pdf]

ONLINE RESOURCE 2 – Search Strand

Assessments related to the physical, affective and cognitive domains of physical literacy among children aged 7-11.9 years: a systematic review

[redacted]

Search strand

	Assess* OR Measure* OR Test* OR Tool* OR Instrument* OR Battery* OR Method* OR Psychometr* OR Observ* OR Indicator* OR Evaluat* OR Valid* Or Reliab*	Title or Abstract
AND	“Physical* Activ*” OR “Physical* Liter*” OR Play OR Sport OR “Physical* Educat*” OR Exerci* OR Recreation	All Text
AND	Child* OR Youth OR Adoles* OR P$ediatric* OR Schoolchild* OR Boy* OR Girl* OR Preschool* OR Juvenile* OR Teenager	All Text
AND	Motiv* OR Enjoy* OR Confidence OR Self* Or “Perc* Competence” OR Affective OR Social OR Emotion* OR Attitude* OR Belief* OR Physical* OR Fitness OR Motor OR Movement* OR Skills* OR Technique* OR Mastery OR Abilt* OR Coordination OR Performance OR “Perceptual Motor” OR Knowledge OR Understanding OR Value OR Cogniti* OR Health OR Well*	All Text
