# Supplementary material for: Assessments Related to the Physical, Affective and Cognitive Domains of Physical Literacy Amongst Children Aged 7–11.9 Years: A Systematic Review
Source: Sports Med Open. 2021 May 27;7:37. doi: 10.1186/s40798-021-00324-8 (PMC8160065; doi:10.1186/s40798-021-00324-8)
Supplement: Supplementary file 3 — Additional file 3. [file 40798_2021_324_MOESM3_ESM.pdf]

ONLINE RESOURCE 3 – COSMIN quality criteria for rating of measurement properties

Assessments related to the physical, affective and cognitive domains of physical literacy among children aged 7-11.9 years: a systematic review

[redacted]

Online Resource 3 Quality criteria for rating of measurement properties

Psychometric properties	Definition	Rating	Quality criteria
**Reliability**	The degree to which the measurement is free from measurement error (Prinsen et al., 2016)	+<br>-<br>?	ICC OR weighted Kappa *r* >0.70<br>ICC OR weighted Kappa *r* <0.70<br>ICC OR weighted kappa not reported
**Internal consistency**	The extent to which items in a (sub)scale are intercorrelated, thus measuring the same construct (Terwee et al., 2007)	+<br>-<br>?	(Sub)scale unidimensional AND Cronbach alpha >0.70<br>(Sub)scale not unidimensional OR Cronbach alpha <0.70<br>Dimensionality not known OR Cronbach alpha not determined
**Content validity**	The extent to which the domain of interest is comprehensively sampled by the items in the measurement instrument (Terwee et al., 2007).	+<br>-<br>?	The target population considers all items in the measurement instrument to be relevant AND considers the tool to be complete<br>The target population considers all items in the measurement instrument to be irrelevant OR considers the tool to be incomplete<br>No target population involvement
**Construct / structural validity**	The degree to which the scores of a measurement instrument are an adequate reflection of the dimensionality of the construct to be measured (Prinsen et al., 2016)	+<br>-<br>?	Factors should explain at least 50% of the variance<br>Factors explain <50% of the variance<br>Explained variance not mentioned
**Cross-cultural validity**	The degree to which the performance of the items on a translated or culturally adapted measurement instrument is an adequate reflection of the performance of the items of the original version of the measurement instrument (Prinsen et al., 2016)	+<br>-<br>?	No important differences found between group factors (such as age, gender, language) in multiple group factor analysis OR no important DIF for group factors (McFadden's R^2 <0.02)<br>Important differences between group factors OR DIF was found<br>No multiple group factor analysis OR DIF analysis performed

**Criterion/Concurrent validity**	The extent to which scores on a particular measurement instrument relate to an alternative, previously validated measure of the same construct (Robertson et al., 2017) or a gold standard (Terwee et al., 2007)	+	Convincing arguments that gold standard is ‘‘gold’’ OR alternative measure has been previously validated AND correlation with gold standard OR alternative measure >0.70
		-	Correlation with gold standard OR alternative measure <0.70 despite adequate design and method
		?	No convincing arguments that gold standard is ‘‘gold’’ OR alternative measure has been validated OR doubtful design or method

The criteria are based on Robertson et al. (2017), Prinsen et al. (2016),, Terwee et al. (2007) and Mokkink et al. (2010)

ICC = Intraclass correlation; ROC = receiver operating characteristic; DIF = differential item functioning; SDC = smallest detectable change; MIC = minimum important change; LOA = limits of agreement; RR = relative risk; ACU = area under the curve.

+ = positive rating; - = negative rating; ? = indeterminate rating.
