# Supplementary material for: Assessments Related to the Physical, Affective and Cognitive Domains of Physical Literacy Amongst Children Aged 7–11.9 Years: A Systematic Review
Source: Sports Med Open. 2021 May 27;7:37. doi: 10.1186/s40798-021-00324-8 (PMC8160065; doi:10.1186/s40798-021-00324-8)
Supplement: Supplementary file 4 — Additional file 4. [file 40798_2021_324_MOESM4_ESM.pdf]

ONLINE RESOURCE 4 – Validity data extracted from included studies

Assessments related to the physical, affective and cognitive domains of physical literacy among children aged 7-11.9 years: a systematic review

[redacted]

Online Resource 4 Validity Results of Included Assessments

	Assessment	Content Validity	Construct Validity	Criterion Validity
**All Domains**				
Longmuir 2017	CAPL-2 (CAMSA)	Delphi Expert consultations were combined with an environmental scan of physical education curricula and published research to develop the activities included in the initial assessment; An international expert advisory panel comprised of physical educators and physical activity scientists selected a dynamic series of movement skills as the preferred format. The final Delphi panel had 25 ± 15 years of research experience within their field (range: 5 to 65 years) and a combined total of 4,181 peer-reviewed publications (range: 15 to 1,500 publications per expert). All Delphi participants held an academic appointment (professor emeritus = 4, professor = 10, associate professor = 3, assistant professor = 2), many with cross-disciplinary areas of expertise	In a multivariable model ($p < 0.001$, η2 = 0.17, R2 = 0.16), older age ($p < 0.001$, η2 = 0.15) and male gender ($p < 0.001$, η2 = 0.02) were significantly associated with a higher total assessment score The same pattern of associations was observed for the skill ($p < 0.001$, η2 = 0.04, R2 = 0.04) and time ($p < 0.001$, η2 = 0.20, R2 = 0.20) scores.<br>Higher Total scores for:<br>older age ($p < 0.001$, η2 = 0.15) male gender ($p < 0.001$, η2 = 0.02)<br>Increasing age corresponded to higher skills scores<br>($p < 0.001$, η2 = 0.04,<br>R2 = 0.04) and quicker completion time ($p < 0.001$, η2 = 0.20, R2 = 0.20) scores	
Dania 2020	CAPL-2		Confirmatory factor analyses (CFAs) were performed sequentially in two stages. First, a CFA was performed to examine the factor structure for each CAPL-2 domain separately. At a second stage, the models from each individual domain's CFA were combined in a four-factor correlated measurement model. The four domain models that were tested individually were: PC (three indicators); DB (two indicators); K&U (five indicators); and M&C (four indicators). Since not all indicators could be specified as continuous variables, the asymptotically distribution free estimator was used.<br><br>Variance was constrained to one before setting the metric of latent factors. A combination of goodness-of-fit statistics was used to test data-model fit, including the model chi-square (χ2), the root mean square error of approximation (RMSEA) with the 90% confidence interval (CI) (RMSEA < 0.06 or less for good fit), the comparative fit index (CFI close to 0.95 or greater for good fit), and the Tucker–Lewis index (TLI) (TLI close to 0.95 or greater for good fit) (Brown, 2006). When the fit of a nested model was poor, modification indices were examined and were interpreted only in relation to relevant theory or prior research. CFA was conducted using AMOS 26.0 software.<br><br>Results indicated that the hypothesized CFA model for the K&U domain was a good fit for the data, χ2(5) = 2.88, p = 0.718, RMSEA = 0.00 [90% CI (0.000, 0.043), p = 0.974]; CFI = 1.000; TLI = 1.041. Furthermore, the CFA model run for the M&C domain generated fit statistics that were satisfactory at an acceptable range, χ2(48) = 96.482, $p < 0.01$, RMSEA = 0.04 [90% CI (0.030, 0.054), p = 0.856]; CFI = 0.844; TLI = 0.785. The CFA model run for the DB domain could not be estimated, since it had only two indicators (pedometer counts and self-reported PA) (Brown, 2006). However, due to the information gained by scores in this domain, a decision was made to further examine its factor loadings in the full measurement model. Finally, the CFA model that was run for the PC domain was estimated with PACER, CAMSA, and plank as indicators and was just identified.	
Gunnel 2018a		Children (n = 205, Mage = 9.50 years, SD = 1.14 years, 50.7% girls) who were enrolled in YMCA free summer camps in southwestern Ontario completed the CAPL-2 (see	Confirmatory factor analyses were calculated separately for each individual measurement scale. Coefficient H and omega total were calculated as estimates of score reliability for each subscale	

	[5] plus the revised surveys in Additional files 1 and 2). The revised surveys were the original CAPL measures of motivation (Survey 1), and the self-determination theory-based measures of motivation plus the revised CAPL Knowledge and Understanding questionnaire	(formulas provided in Additional file 5). Coefficient H is an assessment of maximal reliability based on factor loadings derived from the factor analysis, assuming optimal weighting (i.e., every item contributes different amounts to the total scale score) [29]. Omega total is an assessment of reliability based on factor loadings and error variances that assume unit-weighting (i.e., every item contributes equally to the total scale score) [30]. Both indicators of reliability are presented to inform readers because they are superior to alpha, assume congeneric models, and provide different information depending on the goal of the researcher. For example, coefficient H will provide an estimate of score reliability assuming a researcher is using optimal weighting (e.g., through factor analysis), whereas omega total will provide an estimate of reliability assuming the researcher is adding up raw items to create a total scale score (e.g., using manifest variable models such as regression).<br><br>In the second step, confirmatory factor analyses specifying measurement models, which comprised various combinations of motivation and confidence based on composite scores, were specified and evaluated. Composite scores were used in this step given the complexity of the overall CAPL models and the small sample size. Two models hypothesized a priori were tested. The first was the original CAPL-1 model excluding activity compared to others (i.e., composite scores of adequacy, predilection, benefits-to-barriers difference, and skill compared to others; Survey 1). The second was the self-determination theory-based measures (i.e., composite scores of intrinsic, identified, introjected, and external regulation as well as perceived competence satisfaction; Survey 2). Other exploratory models were informed by the results of the individual confirmatory factor analysis in Step 1, and comprised of a mix of questionnaires from Survey 1 and Survey 2.<br><br>In the third step, the final selected model from Step 2 was entered into a measurement model with all other CAPL-2 domains, to determine if the revised Motivation and Confidence domain demonstrated a good fit with the other CAPL-2 domains.<br><br>Results of the four-correlated factor model indicated that the model was an excellent fit to the data (MLR$\chi^2(60) = 66.30$, $p = 0.27$, $CFI = 0.969$, $RMSEA = 0.023$, 90% CI [0.00, 0.050]). The Knowledge and Understanding indictor asking “how to improve sport skill” did not significantly load onto knowledge and understanding ($\lambda = 0.19$, $p = 0.13$). All other factor loadings were significant ($\lambda = 0.30$–.92, $ps < 0.05$). Daily step count was not significantly correlated with any CAPL domain ($ps > 0.14$). Physical Competence was correlated with Knowledge and Understanding ($r = 0.43$, $p < 0.001$) and Motivation and Confidence ($r = 0.29$, $p = 0.002$). Motivation and Confidence was uncorrelated with knowledge and understanding ($r = 0.14$, $p = 0.25$).
Gunnel 2018b		Based on weak factor loadings ($\lambda s < 0.32$) and conceptual alignment, we removed body mass index, waist circumference, sit-and-reach flexibility, and grip strength as indicators of Physical Competence. Based on the factor loading ($\lambda < 0.35$) and conceptual alignment, we removed screen time as an indicator of Daily Behaviour. To reduce redundancy, we removed children’s activity compared to other children as an indicator of Motivation and Confidence. Based on low factor loadings ($\lambda s < 0.35$) and conceptual alignment, we removed knowledge of screen time guidelines, what it means to be healthy, how to improve fitness,

			activity preferences, and physical activity safety gear indicators from the Knowledge and Understanding domain. The final refined CAPL model was comprised of 14 indicators, and the four-factor correlated model fit the data well (r ranged from 0.08 to 0.76), albeit with an unexpected cross-loading from Daily Behaviour to knowledge of physical activity guidelines (mean- and variance-adjusted weighted least square [WLSMV] $\chi2(70) = 1221.29$, $p < 0.001$, Comparative Fit Index [CFI] = 0.947, root mean square error of approximation [RMSEA] = 0.041[0.039, 0.043]). Finally, our higher-order model with Physical Literacy as a factor with indicators of Physical Competence ($\lambda = 0.68$), Daily Behaviour ($\lambda = 0.91$), Motivation and Confidence ($\lambda = 0.80$), and Knowledge and Understanding ($\lambda = 0.21$) fit the data well.
Longmuir 2018b	CAPL-2	For each domain of the CAPL, recommended changes based on the factor analyses, qualitative feedback and theoretical considerations significantly reduced the number of protocols. Specific protocol combinations were then evaluated for model fit within the overarching concept of physical literacy. The CAPL-2 continues to reflect the four components of the Canadian consensus definition of physical literacy: Motivation and Confidence, Physical Competence, Knowledge and Understanding, and engagement in Physical Activity Behaviour.	
Longmuir 2018c	CAPL-2 (PLKQ)	Content areas for the PLKQ were systematically identified through a review of physical and health education curricula from all Canadian provinces and territories, supplemented by the recommendations of an international Delphi process [13]. Key learning objectives from each curriculum document for grades 4, 5, or 6 were identified (Table 1). A content analysis of the key learning objectives identified the following areas of knowledge as being common across all of the reviewed curricula: importance of physical activity, definition of cardiorespiratory fitness, guidelines for daily physical activity and sedentary time, definition of “healthy”, recognition of movement skills, understanding of fitness and its impact on physical activity, safety practices during physical activity, identification of healthy foods, and methods of skill and fitness improvement. Open-ended questions on the proposed topics to be assessed were then provided to children in grades 4, 5, and 6 as well as their teachers (Additional file 1). Feedback was obtained on the clarity and wording of the questions from both teachers and students. In addition, the children’s responses to the open-ended questions were used to identify the closed-ended response options that would be included in the initial PLKQ<br><br>Content for the initial PLKQ was verified through an international Delphi process [13]. Experts in children’s physical activity, movement, motivation, and fitness achieved consensus on initial PLKQ content through an iterative process. Results from administration of the initial PLKQ to children assessed in schools in eastern Ontario were analyzed by the child’s self-reported age and gender. It was hypothesized that knowledge would not vary by gender but would increase with age.<br><br>The Delphi expert panel ($n = 19$, 4 female [21%]), who had 25 ± 15 years of research experience within their field (range: 5 to 65 years) and a combined total of 4181 peer-reviewed	

		publications (range 15 to 1500), agreed that children's knowledge of daily physical activity and screen time guidelines, the meaning of cardiorespiratory fitness and muscular strength and endurance, and how to improve sport skills and fitness were important areas of knowledge that should be included in the PLKQ.	
**Lodewyk 2017**	PFL	These four representative components of physical literacy in PFL were identified during a consultative process lasting several years and align with PHE Canada's definition of physical literacy. The Lodewyk and Mandigo 459 development of PFL involved an extensive consultation process. In January 2011, PHE Canada invited 15 to 20 PE experts from across Canada to meet to discuss the potential of a physical literacy assessment tool to be used in schools across Canada and, if recommended, what the guiding principles might be. Based on the outcomes of that meeting, in the summer of 2011 PHE Canada organized a gathering of six Canadian professor-researchers with expertise in PE curricula and pedagogy, physical literacy, and assessment, along with several school-based PE teacher-educator leaders, to develop PFL based on the guiding principles and its definition of physical literacy. This led to the formulation of the names and basic composition of the four components of PFL (Active Participation; Movement, Fitness, and Living Skills). Cognition was not a component, because it was viewed as being integrated and evident in the four components. Five members of this group then wrote and developed each of the component assessments and also met occasionally to review, plan, and provide guidance on PFL. This writing team represented a balance of expertise in each PFL component from across Canada. Each writer also had practical teaching-coaching experiences with children and adolescents in physical activity settings, four were certified specialists in physical and health education, and three were professor-researchers with terminal degrees in a relevant field (e.g., PE). Several iterations of each assessment were made based on feedback from the writers. The results of the pilot test with Grade 4 and 5 students revealed that PFL data by measure were normally distributed (e.g., no ceiling or floor effect evident in skew or kurtosis) and had satisfactory concurrent validity (e.g., theoretically expected correlations among constructs and measures). For example, Pearson bivariate correlations among and between the three movement skill assessments and three fitness skill assessments were positive and statistically significant ($p < .001$) ranging from .28 to .45. Additionally, these skills correlated positively and significantly ($p < .01$) to students' self-reported participation levels in fitness activities at school ($r = .11–.20$). A comparison sample of 20 students from a fifth grade class was used to test the interrater and test–retest reliability and provide validity evidence of concurrent relations with PFL fitness and movement skill measures. Based on the feedback from this pilot test, modifications were made (most notably to the content of the living skills items) and expanded to include Grade 3 students with the Grades 4 to 6 assessment for full implementation in the fall of 2013–2014, along with a new PFL for Grades 7 to 9. Following the pilot of PFL with Grades 4 to 5 in 2012–2013 and revisions stemming from it, PFL was administered to	The descriptive statistics (i.e., means, standard deviations, skew, and kurtosis) revealed no abnormalities in any of the items or scales, particularly for large sample sizes (Tabachnick & Fidell, 2006), except for a consistently (by year and developmental level) negative skew and kurtosis (> 1.00) for the intentions to be physically active item 464 Initial Validation of Passport for Life in active participation. Consequently, this item was not included in subsequent analyses. Another source of validation evidence is the degree that scores from the assessment items align (interrelate) with the construct being assessed relative to the intended interpretation of the assessments (Standards; AERA, APA, & NCME, 2014). We used principal component exploratory factor analyses loading onto a single factor and suppressing factor loadings < .30 to explore the factor structure for each living skill scale (feeling, thinking, and interacting) by year and level (Grades 3 to 6, Grades 7 to 9). Because of the large sample sizes for the first assessment of each year relative to those lower than recommended for factor analysis (< 250) in the second assessment of each year (Tabachnick & Fidell, 2006), we used only the first assessment by year and level. Table 2 shows the results. Each item in each scale had strong factor loadings (.53–.81), which explained a satisfactory proportion of the variance (42.07–54.53%). Especially for scales with fewer than 10 items (Loewenthal, 1996), the internal consistency reliability coefficients (see Table 3) for each of the three scales by year and level were satisfactory (living skills, .75 to .86; active participation, .61 to .87; movement skills, .64 to .75; fitness skills, .62 to .77). Noting the likelihood of maturation and other effects (e.g., motivational and seasonal) between repeated assessment times in the fall and spring seasons within each academic year (2013–2014, 2014–2015) and developmental level (Grades 3 to 6, Grades 7 to 9), we computed and reported test–retest reliability coefficients for the assessment scales to potentially signal the stability of measurement scores. We aligned the criterion with Loewenthal's (1996) standards for scales with fewer than 10 items, setting it at >.43 for an interitem test–retest correlation coefficient and > .60 for an internal consistency reliability coefficient.

		Grades 3 to 6 and Grades 7 to 9 in 2013–2014 and 2014–2015.		
Cairney 2017	PLAYfun		To evaluate the published factor structure of the instrument, we used confirmatory factor analysis. Because PLAYfun is only one component of a comprehensive measure of physical literacy, we chose a correlated traits model to assess fit to the data. Our model is based on the PLAYfun manual, which groups tasks explicitly into five domains: 1) running, 2) locomotor, 3) object control—upper body, 4) object control—lower body, and 5) balance, stability, and body control. We tested this hypothesized factor structure, allowing all factors to be correlated with the others, with tasks treated as manifest indicators. We fit models using maximum likelihood estimation.<br><br>The fit of the initial model was fair (RMSEA, 0.065; 90% confidence interval, 0.052–0.077; CFI, 0.93; TLI, 0.91). Modification indices suggested several ways the model could be adjusted, but most would have represented post hoc adjustments without truly clear and obvious justifications. One change, however, was clearly reasonable: adding a path to allow error terms for tasks 15 and 16 to covary. These items load on the same factor and more importantly are categorically identical (body control and balance), with the direction of movement (forward and backward) being the only distinction. As it seemed clear that a particularly close relationship could be expected between these items, we added this path and refit the model. The adjusted model is shown in Figure 1. This modification improved fit indices somewhat (RMSEA, 0.055; 90% confidence interval, 0.03–0.075; CFI, 0.95; TLI, 0.94).	
Stearns 2020				Convergent validity statistics, including the relationships between the PLAYfun and PLAYbasic tools with the PAQ-C and the CAMSA obstacle course are reported in Table 5. Correlations with the CAMSA obstacle course were moderate-to-large for the PLAYfun (r = .47–.60) and the PLAYbasic (r = .40–.61), and were small-to- moderate for all of the subscales. Correlations with the PAQ-C were small-to-medium for the PLAYfun (r = .24–.44) and the PLAYbasic (r = .20–.42), and were moderate for all of the subscales. Correlations between PLAYbasic and PLAYfun were large (r = .83–.90). Therefore, as expected, the PLAY tools correlate with the CAMSA obstacle course and self-reported physical activity
**Affective Domain**				
Cumming et al 2008	AGSYS	Pilot focus group conducted. A preliminary trial during which 13 young athletes between the ages of 8 and 11 years were asked to read potential items and identify any they did not understand resulted in the rewriting of 2 items.	9-10 year olds df=53, IFI+.93, CFI= 0.93, GFI=.92, SRMR=0.068, RMSEA=.063, RMSEA 90%CI=.046-.080<br><br>11-12 years olds df=53, IFI+.95, CFI= 0.95, GFI=.94, SRMR=0.067, RMSEA=.059, RMSEA 90%CI=.048-.070	
Bornholt & Ingram 2001	ASK-KIDS	Items were developed from a context outside PA.		
Bornholt & Piccolo, 2005	ASK-KIDS		Children's self concepts were a good fit (ratio χ^2/df = 1.5, RMSEA = .07) and responses ranged widely.	
Jones, 1988	ATCPE	Extensive pool of items generated from pupils in schools. Wang's criteria for the selection of attitude scale items were applied and an initial pilot scale was established using 20		

		statements. The pilot 20-item scale was tested with a sample of 233 subjects aged nine to twelve (Test 1) and then used again with a different sample of 35 children on a test-retest basis (Test 2).	
Beyer et al., 2015	ATOP	1) item generation based on a comprehensive literature review and consensus among the project team, (2) interviews with environmental educators, (3) initial pilot testing, (4) scale refinement	Factor analyses revealed two underlying factors (eigen values e Factor 1: 2.83, Factor 2: 1.89). The factors were weakly negatively correlated (r = 0.2), and a multilevel factor analysis demonstrated that the two factors did not measure one underlying construct
Sebire et al., 2013	BREQ	Adapted from questionnaire used with another population. Items were screened individually for age appropriateness and simplifications to wording based on published measures of children's self-determined motivation in other contexts (e.g., academic subjects) [32] and references to exercise were replaced with PA. To reduce participant burden, 12 items (3 per motivation subscale) were specified. The items were screened by three academics with expertise in children's motivation, development and PA who provided feedback on theoretical alignment, construct coverage and item clarity. Feedback on both of the new scales was sought from two primary school teachers with regards to clarity of the items. Flesch-Kincaid Grade Level reading scores (based on average sentence length & number of syllables) indicated that the reading age was appropriate for the target age group.	PA behavioural regulation scale model 1 df=46, CFI= 0.981, SRMR=0.032, RMSEA=.037 (.020, .051)<br>PA psychological need satisfaction scale model 1 df=132, CFI= 0.929, SRMR=0.044, RMSEA=.055 (.047, .063)<br>PA psychological need satisfaction scale model 2 df=101, CFI= 0.940, SRMR=0.041, RMSEA=.057 (.049, .066)<br>PA psychological need satisfaction scale model 3 df=99, CFI= 0.951, SRMR=0.040, RMSEA=.052 (.040, .060)
Brustad 1996	CAPA	Various dimensions of physical activity that children might regard as attractive or unattractive were initially identified through open-ended group discussions with third- and fourth-grade children in tbeir physical education classes.	
Simon & Smoll 1974	CATPA	Questions adapted from pervious study in different context/age group. One subdomain removed from the existing questionnaire	
Schultz et al 1981			Factor analyses calculated goodness of fit statistic based from a final factor solution based upon the minimum eigenvalue criteria. Seven nontrivial factors are presented, yet the goodness of fit statistic was calculated on a final factor solution of 10 factors. Goodness of Fit df=693, X2=2123.09 Tucker *R*=.93
DeBate 2099	CPAS	Questions adapted from pervious study in different context/age group. But no details given on this adaptation process	EFA conducted but reporting unclear
Welk et al 1997	CY-PSPP	Questionnaire Adapted from assessment used in adults, was adapted for adolescents, then adapted for younger children. In this study, two versions were offered. The original version consisted of 36 questions4 questions for each of the five scales of the CY-PSPP, and 6 for a global measure of self-esteem (Rosenberg, 1965)-written in a 4 point structured alternative format. The child first determined which of two hypothetical children he or she was most like, and then decided whether it was 'really true or just sort of true for him or her.' The modified version featured the same number of questions and similar terminology, but used a standard 4-point Likert scale. The child read a description of a hypothetical child and answered whether this was really true for me, somewhat true for me, sort of true for me, or not true for me. Questions in the modified version were worded both positively or negatively to keep it similar to the original version.	The factor structure was much clearer for the original version than for the modified version. The four factors for the original version all had eigenvalues greater than 1 .O, and collectively explained 60% of the variance in the PSPP scores. The factor loadings on the original version were generally high, with only three items not loading on their intended factor. Some cross loadings were apparent, particularly between the Condition and Sport domains and the Sport and Body domains, but the extent was not greater than previously noted. The factors on the modified version collectively explained 50% of the variance, but there were many small loadings and numerous cross loadings among the intended domains. Factor 1 Original version Eigenvalue=9.5, Variance 39.5%. Factor 2 Original Version Eigenvalue=2.3, Variance 9.5%. Factor 3 Original Version Eigenvalue=1.4, Variance 5.8%. Factor 4 Original Version Eigenvalue=1.2, Variance 5.0%. Factor 1 Modified Version Eigenvalue=2.0, Variance 8.3%. Factor 2 Modified Version Eigenvalue=6.8, Variance 28.1%. Factor 3 Modified Version Eigenvalue=1.5, Variance 6.4%, Factor 4 Modified Version Eigenvalue=1.8, Variance 7.4%<br><br>To examine convergent validity, Pearson product-moment correlations were computed between the subscale scores of the two

			different versions were high (range: r = .74 to .78) for all of the scales indicating a fairly close association between the two versions. Mean correlations were higher for the girls, compared with the boys (r = .78 and r = .70, respectively). Overall, the two instruments appear to be measuring the same constructs.
Welk et al 2005			Measurement model;<br>X21375.68, df 579, p<0.001, NNFI 0.915, CFI, 0.922, RMSEA (90% CI) 0.043 (0.040-0.046)<br>Structural model;<br>X21513.32, df 589, p<0.001, NNFI 0.902, CFI, 0.908, RMSEA (90% CI) 0.0436(0.043-0.048)
**Chen 2011**	DPAPI		Evaluation of the overall model fit of the 10-factor measurement model indicated an excellent model fit result.<br>Although *X2*statistics was used to examine the overall fit of the model to the data, this study used the ratio of chisquare to degrees of freedom (*X2*/*df*) to evaluate the overall model fit. The low value of *X2/df* ranged from 2.0 to 5.0 is considered as an indicator of reasonable model fit (Kline, 2005). In this study, while the chi-square of the 10-factor measurement model was significantly different from the observed data (*X2= 497.32, df = 207, p<.01)*, the ratio of chi-square to degrees of freedom (*X2*/*df* = 2.4) indicated that the proposed model adequately fitted the observed sample data. Examination of the multiple goodness-of-fit indices for the 10-factor measurement model further indicated excellent fit to the sample data. Both NNFI and CFI with values of .95 and .97 indicated an excellent model fit to the data (Meyers et al., 2006). The value of RMSEA was 0.057 (90% CI = .05: .06) and the value of SRMR was .077, indicating good model fit to the data (Kline, 2005). The overall model fit of the second order 3-factor measurement model revealed an adequate fit to the observed data (*X2*= 833.456, *df* = 197; *X2/df* = 4.23). However, the fit indices for the second-order factor model yielded a marginal fit to the data (NNFI = .85, CFI = .88, RMSEA = .086, SRMR = .086).
Shewmake 2015	EnjoyPE	The 10 survey statements were randomly mixed and sent to a panel of five experts. The experts, averaging 18 years of experience in the field of PE, were asked to group like questions to ensure validity of the survey instrument. The panel of experts found the survey instrument to have sufficient validity. Four of the five PE experts grouped the three perceived exertion statements together (with the fifth expert excluding only one of those three statements), thus leaving the remaining seven statements to form the perceived enjoyment group	The fit indices for the final latent class analysis for psychosocial determinants were: RMSEA= 0.044 (confidence interval, 0.041–0.048), CFI = 0.911, and TLI = 0.906, which indicated a good model fit.
Gray et al 2016	FHC-Q	Interviews, observations and pilot testing reported, little detail offered	CFA for psychosocial determinants ranged from 0.36 to 0.87, all below 0.95 threshold
Bornholt 2005	FAPM	Items were developed from a context outside PA	
Rosenkranz 2011	HOP’N		
Hyndman 2013	LEAP	Literature review. Consideration of socioecological model. Consulted teacher and 5 PA experts with experience in the development of self-report measures	
	MAAP	Based on previous questionnaires used in a different context and populations and based on previous ecological momentary assessment studies. Pilot conducted with 8 children but little detail offered.	
Rose 2002	MOSS		For the whole sample five interpretable factors were derived that

			had eigenvalues greater than 1 and accounted for more than 4 percent of the variance. Only those items loading .3 or above were included.<br>Factor 1 Eigenvalue 7.1, 26.5% variance<br>Factor 2 Eigenvalue 2.7 10.0% variance<br>Factor 3 Eigenvalue 1.6, 6.1% variance<br>Factor 4 Eigenvalue 1.4, 5.2% variance<br>Factor 5 Eigenvalue 1.2, 4.3% variance
Nelson 2009	NAS	Adapted from instrument validated in adolescent population	This model demonstrated acceptable fit, χ^2 (76, N = 178) = 159.54, p < .001; RMSEA= .079 (CI90% .062=.096), CFI= .93; NNFI = .92, and the latent correlation between positive and negative attitudes was .50. The two-factor model was then compared to an alternative one-factor model using a nested model comparison technique. The latent correlation between positive attitudes and negative attitudes was constrained to -1.0 to test the model assuming that positive and negative attitudes represent a unitary (but perfectly negatively correlated) construct. The one-factor model fit poorly, χ^2 (77, N = 178) = 266.82, p < .001; RMSEA = .137 (CI90 = .122-.153); CFI = .79; NNFI = .75 and represented a significant degradation in fit compared to the two-factor model using the χ2 difference test
Dishman 2013	PABM	Adapted from instruments validated in different populations	In fifth grade, the hypothesized structural measurement model had acceptable fit (χ2 (621) = 1224.8, CFI = 0.929, RMSEA = 0.031 [0.029–0.034], SRMR = 0.042). Physical activity was related to self-efficacy (b = 0.190, SE = 0.053, p < .001), evaluation barriers (b = 0.178, SE = 0.053, p = .001), parental support (b = 0.125, SE = 0.052, p = .016), enjoyment (b = 0.152 SE = 0.042, p < .001), and competence (b = 0.118, SE = 0.062, p = .05). Multi-group models had acceptable fit between boys and girls (χ2 (1243) = 2107.5, CFI = 0.903, RMSEA = 0.037 [0.035–0.040], SRMR = 0.053) and between non-Hispanic black and white students (χ2 (1243) = 174.2, CFI = 0.918, RMSEA = 0.035. [0.031–0.038], SRMR = 0.055). Nested models indicated that the path coefficients between physical activity and the beliefs and motives were not different according to gender or race (χ2 , p > .10; CFI < 0.01). Results were not substantively changed after including BMIz in the models. Bivariate relations of the variables with physical activity and BMIz are provided in Supplementary Table S9. The hypothesized model also had acceptable fit in the sixth grade (χ2 (621) = 1248.4, CFI = 0.938, RMSEA = 0.034 [0.032–0.037], SRMR = 0.047). Physical activity was related to self-efficacy (b = 0.208, SE = 0.037, p < .001), evaluation barriers (b = 0.212, SE = 0.047, p < .001), outcome barriers (b = 0.126, SE = 0.036, p < .001), parental support (b = 0.166, SE = 0.052, p = .001), enjoyment (b = 0.108, SE = 0.035, p = .002), and competence (b = 0.107, SE = 0.037, p = .004). Multi-group models had acceptable fit between boys and girls (χ2 (1243) = 2191.9, CFI = 0.911, RMSEA = 0.042 [0.039–0.045], SRMR = 0.057) and between nonHispanic black and white students (χ2 (1243) = 2006.8, CFI = 0.910, RMSEA = 0.044 [0.041–0.048], SRMR = 0.059). Nested models indicated that the path coefficients between physical activity and the beliefs and motives were not different according to gender or race (χ2 , p > .10; CFI < 0.01). Results were not substantively changed after including BMIz in the models. The sixth grade panel model that adjusted for fifth grade relations in the longitudinal cohort had similar fit (χ2 (1,161) = 1744.6, CFI = 0.945, RMSEA = 0.025 [0.023–0.028], SRMR = 0.042)
Moore 2009	PACES	Adapted from instrument validated in adolescent population. Pilot testing	Model 1, which represents a single-factor model, provided a poor fit to the data (X2(104) = 1495.44, RMSEA =.149 [90% CI

			=.140 – .160], CFI = .89, NNFI = .87).<br>Model 2, which represents a two factor model, exhibited acceptable indices of fit (X2(103) = 228.25, RMSEA = .045 [90% CI = .037 – .053], CFI = .98, NNFI = .98). Model 3 (X2(83) = 149.85, RMSEA = .038 [90% CI = .028 – .048], CFI = .99, NNFI = .99).<br>Model4 (X2(68) = 150.45, RMSEA = .047 [90% CI = .037 – .057], CFI = .99, NNFI = .98).<br>Convergent Validity<br>Enjoyment of PA was significantly correlated with task goal orientation (r = .66, p < .01; r = .66, p < .01), as well as perceptions of athletic competence (r = .24, p < .01; r = .25, p < .01), physical appearance (r = .21, p < .01; r = .23, p < .01), and self-reported physical activity (r = .17, p < .01; r = .16, p < .01) both before and after controlling for sex and race, respectively
Perry 2008	PAHFE	Expert review, interviews, pilot testing, cognitive interviews	Pretest: four factors CFI 0.756, RMSEA 0.096, Three factors CFI 0.745, RMSEA, 0.090, Two factors CFI 0.671, RMSEA 0.097.<br>Posttest: four factors CFI 0.894, RMSEA 0.065, Three factors CFI 0.891, RMSEA, 0.066, Two factors CFI 0.849, RMSEA 0.069
Nelson 2009	PAS	Adapted from instrument validated in adolescent population	This model demonstrated acceptable fit, χ^2 (76, N = 178) = 159.54, p < .001; RMSEA= .079 (CI90% .062=.096), CFI= .93; NNFI = .92, and the latent correlation between positive and negative attitudes was .50. The two-factor model was then compared to an alternative one-factor model using a nested model comparison technique. The latent correlation between positive attitudes and negative attitudes was constrained to -1.0 to test the model assuming that positive and negative attitudes represent a unitary (but perfectly negatively correlated) construct. The one-factor model fit poorly, χ^2 (77, N = 178) = 266.82, p < .001; RMSEA = .137 (CI90 = .122-.153); CFI = .79; NNFI = .75 and represented a significant degradation in fit compared to the two-factor model using the $\chi 2$ difference test
Jago 2009	PASE	Started with previous physical activity and sedentary behaviour self efficacy scales. Following theory specified procedures, easy and difficult versions for each behaviour were then generated for each item. This process was started by one of the co-authors and then reviewed by several of the other authors as a multidisciplinary expert panel. Several iterations of item development were conducted until all were satisfied with the items.	Exploratory Factor Analysis conducted. The corrected item total correlations (CITC) indicate the extent to which the item can discriminate between participants with low and high physical activity self-efficacy. All of the CITC scores were above 0.41 (scores that are greater than 0.30 [36]are considered to be excellent). Factor analysis indicated that a one factor solution explained 46.4% of the variance in the items with the two factor solution explaining only 8.5%more of the variance. The factor analysis therefore showed that all of the items loaded onto one factor and that the instrument was assessing a cohesive construct. Therefore the assumption of sufficient unidimensionality was satisfied. The individual factor loadings for each item when the dominant one factor solution was used ranged from 0.57 to 0.73and the alpha for this scale was 0.90 suggesting that the items were assessing the same construct.
Saunders 1997	PASES	Based on questionnaires used with adults and adolescents. A series of pilot testing and refinement.	Principal component factor analysis was used<br>The Social Influences scale contained 8 items. A single factor emerged from this analysis. All items loaded at least 0.40 on the scale.<br>The Self-Efficacy scale contained 17 items. Three factors emerged from this analysis: support seeking, barriers, and positive alternatives. All items loaded at least 0.35<br>Beliefs scale contained 16 items. While 3 factors emerged from the scree plot analysis, the 2-factor solution was selected because the 3-factor solution had several items with multiple loadings, and the 2-factor solution was more readily interpretable. The 2 factors were labelled social outcomes and physical outcome
Liang 2014	Physical Activity Self-	Based on questionnaires used in different populations. Developed questionnaire was then translated and back	Initial CFA model fit criteria for a one-factor model with no correlations across the items in each scale. The CFI for each scale

	efficacy, enjoyment, social support	translation, cognitive interviews and refinement.	was greater than 0.90. SRMR also suggested acceptable model fit.. However, only self-efficacy scale showed acceptable RMSEA. For the enjoyment scale, error covariances were detected between items 3 and 5, items 2 and 7, and items 1 and 4. The model was thereby modified by setting the mentioned parameters free to vary from their previously fixed values of zero. The final model had acceptable model fit indices [Chi square = 29.74,df= 11, RMSEA = 0.08 (90%CI, 0.05–0.11), SRMR = 0.03].For the social support from family scale, error covariance was detected between items 1 and 2. Items 1 and 2 were similar, but not redundant. The final model with parameters set free to vary had accept-able model fit indices [Chi square = 79.35,df= 34, RMSEA = 0.07 (90%CI, 0.05–0.09), SRMR = 0.04].For the social support from friend scale, error covariances were also detected between items 1 and 2, and items 9 and 10. The model was there by modified by setting the mentioned parameters free to vary. The final model with such modifications had acceptable model fit indices [Chi square = 65.90,df= 33, RMSEA = 0.06 (90% CI, 0.04–0.08),SRMR = 0.04]
Vlachopoulos 2011	PLOC in PE		The CFA results indicated a marginal fit of the correlated 5-factor model of the PLOC-R to the data: SeB scaled c2 (406) ¼ 420.71, df ¼ 160, p < .001, SeB c2 /degrees of freedom ¼ 2.62, Robust CFI ¼ .903, Robust RMSEA ¼ .063 (90% CI ¼ .056e.071). The fully standardized item loadings ranged from .48 to .79. Given (a) the marginal fit of the model to the data, (b) the low correlation of the External Regulation #1 item ("Because I'll get into trouble if I don't") with the remaining external regulation subscale items in the internal consistency analyses and (c) the low squared multiple correlation of this item with the remaining items in the internal consistency analyses, the item was removed and the model was re-estimated based on 19 PLOC-R items. The 19-item PLOC-R model (Normalized estimate of Mardia's coefficient of multivariate kurtosis ¼ 64.01) displayed an improved fit to the data: SeB scaled c2 (406) ¼ 324.06, df ¼ 142, p < .001, SeB c2 /degrees of freedom ¼ 2.28, Robust CFI ¼ .927, Robust RMSEA ¼ .056 (90% CI ¼ .048e.064). The fully standardized item loadings ranged from .56 to .80.<br>Cross-validation of the model in Samples 2, 3 and 4 resulted in a reasonable model fit: Sample 2 (elementary school), Mardia's Normalized estimate ¼ 57.51, SeB scaled c2 (411) ¼ 277.22, df ¼ 142, p < .001, SeB c2 /degrees of freedom ¼ 1.95, Robust CFI ¼ .940, Robust RMSEA ¼ .048 (90% CI ¼ .040e.056). The fully standardized item loadings ranged from .52 to .80. Sample 3 (middle school), Mardia's Normalized estimate ¼ 53.12, SeB scaled c2 (471) ¼ 432.07, df ¼ 142, p < .001, SeB c2 /degrees of freedom ¼ 3.04, Robust CFI ¼ .929, Robust RMSEA ¼ .066 (90% CI ¼ .059e.073). The fully standardized item loadings ranged from .50 to .86. Sample 4 (high school), Mardia's Normalized estimate ¼ 58.55, SeB scaled c2 (441) ¼ 386.35, df ¼ 142, p < .001, SeB c2 /degrees of freedom ¼ 2.72, Robust CFI ¼ .936, Robust RMSEA ¼ .063 (90% CI ¼ .055e.070). T
Barnett 2015	PMCS	A pictorial instrument based on the TGMD-2 six locomotor (run, gallop, hop, leap, horizontal jump, and slide); and six object control skills (striking a stationary ball, stationary dribble, kick, catch, overhand throw, and underhand roll) was developed. Newly created subscales assessed locomotor and object control competence using the format and item structure from the physical competence subscale of the Pictorial Scale of Perceived Competence and Acceptance for Young Children.5 The format and structure of the items were retained, but nearly all items (except bouncing a ball) and all	

		illustrations were new. The name of each skill was the same as in the TGMD-2, except for: the slide, which was identified to the children as the ‘step and slide’,the horizontal jump, which was renamed ‘jumping forwards’, the strike which was termed ‘hitting a ball’ and the dribble which was termed ‘bouncing a ball’. This was to facilitate understanding of the movements depicted in the drawings for this young age group. An artist provided drawings that represented ‘poor’ and ‘good’ performances by both boys and girls for each of the 12 skills. So that children from diverse backgrounds could identify with the illustrations, they were drawn in a cartoon format. Correct skill execution was described to the artist in terms of the components as defined by the TGMD-2 (for example bending knees to lower body in the underhand roll).9 The process of development involved the drawings being emailed to each of the authors and an expert in the field (a researcher in the area of movement skills in children) with feedback sought on whether the picture was seen to represent correct (in the case of the ‘good’ picture) and incorrect (in the case of the ‘poor’ picture) execution of the skill. Feedback was collated and given to the artist, whereupon drawings were adjusted and sent again to the authors for approval prior to being tested with children. A generic issue that the expert in the field raised, was that the pictures need to more obviously show difference between good and poor skill performances and there was a concern the differences may be subtle for this age group. For example, it was thought that in the poor catch the child should drop the ball. It was also suggested that the ball should move further away from the child in the poor picture of the bounce to show the child does not have control over the ball. These issues were addressed.	
Lakes & Hoyt 2004	RCS	Literature search and pilot testing reported	Showed weak discriminant validity between the Cognitive and Affective subscales—items on the subscales appeared to load on the same factor, and the subscales had a high correlation (*r* = .90). Items on the Physical/Motor subscale formed a more distinct factor (*r*’s = .75 and .78 with the Cognitive and Affective subscales, respectively).
Lakes 2012			PRS (Person, Rater, Subscale) Analysis:<br>Person= 46% mean variance between occasion one and occasion two<br>Rater = 12% mean variance between occasion one and occasion two<br>Subscale= 3% mean variance between occasion one and occasion two<br>Person, Rater= 14% mean variance between occasion one and occasion two<br>Rater, Subscale = 1% mean variance between occasion one and occasion two<br>Person, Rater, Subscale= 13% mean variance between occasion one and occasion two
	Self-efficacy scale	Four experts in child PA as well as a reading expert were asked to review the survey questions to ensure that (1) items were age appropriate, (2) questions were appropriate for the construct intended, and (3) general readability. The reading expert was chosen for her 10+ years’ experience working with first and second graders and her advanced training as a Reading Specialist. The reading expert was asked to review the study materials for specific reading comprehension issues and identify any problems that might be experienced by children ages 7–9. PA experts were identified as being a part	

		of the original Adolescent or Preschool framework studies or identified as a child PA content expert through a review of the research literature reporting on child PA. One reviewer was unable to participate but referred the study materials to a colleague who was also an expert in child PA. The PA and reading experts received the study materials by email and were asked to respond within 2 weeks. Cognitive testing was used to assess reading comprehension and quality of data collected. The approach to cognitive testing involved two commonly used methods: cognitive interviewing (CI) and field testing (FT). During CI, the research team learned how respondents understood survey questions and allowed for in-depth questioning as to why a specific answer was chosen. FT allowed the researchers to learn how surveys were completed when the researcher was not present to answer respondent questions. This step was especially important since children behave/respond differently based on the adults present. CI and FT participants were recruited through community flyer postings, word of mouth and emails. CI took place during the summer of 2010 and included 10 parent–child dyads. Interviews took approximately 45 min and occurred in locations easily accessible to participating families (e.g. homes, parks, medical facilities). Parent and child respondents completed their respective data collection measures. A research team member using the Self-efficacy Scale and the Activity Log interviewed child participants. Two categories of information were reviewed from the cognitive interviews: comprehension (i.e. how children understood questions) and format (i.e. actual appearance of the data collection tools) issues related to the child-report measures. Children were asked to read the Self-efficacy Scale questions aloud and explain why they chose a specific answer. Children were also asked to place an “X” on any words that they were not familiar. While completing the Activity Log, children were also instructed to list any activities that were not included in the activity log. Parents were instructed to answer the written survey and make notes on any questions which they felt were not clear. Parents also completed a proxy report of the Activity Log detailing this child’s PA over the past 7 days. FT took place in the summer and fall of 2010 and included 22 parent–child dyads. Parents received a letter describing the study and the approximate time commitment to complete the study materials (45 min). In the letter, parents were instructed to review the survey directions with their child and to then allow the child to answer the Self-efficacy Scale and the Activity Log alone. In addition to the parent surveys used in the CI phase, parents also answered six questions about their child’s experience completing the survey.	
Harter 1987	SPPC	The skills represented by items on existing scales included scholastic performance, peer relationships, and a variety of physical skills. These competencies were consistent with our own observations of the mastery behaviors most salient for elementary school children, and thus they provided an initial framework. Interviews with children were then designed to determine which activities within these domains were particularly important to children in making judgments of competence. After initial item revisions, based on feedback from individual children, a 40-item version, 10 items per	One purpose for devising this instrument was to test certain hypotheses in my model of competence motivation. One such hypothesis is that perceived competence should be positively related to ones intrinsic motivational orientation to prefer challenge, to be curious, and to engage in independent mastery attempts (Harter 1978). Clear support for this prediction comes from the correlations between perceived cognitive competence and the three motivational subscales on our measure of intrinsic versus extrinsic orientation in the classroom (Harter 1981b). Perceived cognitive competence is strongly related to preference for challenge ($r = .57$)

		subscale, was group administered to a sample of 215 third through sixth graders.	and to independent mastery (r = .54), and it is moderately related to curiosity (r = .33). Higher-order factoring reveals that perceived cognitive competence, challenge, independent mastery, and curiosity form a distinct factor with very high loadings of .76, .87, .80, and .79, respectively.
Byrne 1988			EFA<br>Factor correlations were partially compatible with those reported by Harter (1982). Consistent with Harter's findings, correlations between the General Self-worth factor and the Cognitive and Social Competence factors were moderate, ranging from - -38 to - -48 (mean r = - .43) and from - .34 to - .47 (mean r - .40), respectively, across subsamples. Likewise, the Social and Physical Competence factors were moderately correlated, with values ranging from .38 to .42 (mean r = .41) across groups. In contrast to Harter's findings, however, correlations between the General Self-worth and Physical Competence factors were low, ranging from - .18 to - .31 (mean r = - .26). Similarly, although correlations between the Cognitive and Social Competence factors were low for Grade 5 students (mean r = .30), they were negligible (mean r = .02) for Grade 8 students.<br>CFA<br>It can be seen that, whereas the hypothesized four-factor model represented a reasonable fit to the data5 (and was considered baseline) for normal student~-~~(98) = 152.26, Grade 5; x2(98) = 132.13, Grade 8- this was not so for the gifted; two item singletons and one item pair were found to have substantial loadings on nontarget factors. Specifically, for gifted students in both grades, item PPC4 loaded on the Social Competence factor, and item PGS4 loaded on the Cognitive Competence factor; item-pair PGS2 loaded on the Social Competence factor for Grade 8 only That the originally hypothesized model (Model 1) did not fit the observed data equally well across ability led to two additional sets of analyses
Devlin 2003			Model 1 (χ2 diff=26; d.f.diff=1; P<0.05)<br>Model 2 (χ2 diff=5; d.f.diff=2; P>0.05)<br>Model 3 (χ2 diff=25; d.f.diff=12; P<0.05)<br>Model 4 (χ2 diff=30; d.f.diff=14; P<0.05)
Klint 1987			The discriminant function analysis for perceived physical competence was significant, Wilks' lambda = .84, F(2, 39) = 3.62, p < .04. The analysis revealed that only two motives entered the stepwise procedure and these included skill development and team atmosphere. Both motives significantly differentiated the groups, as indicated by their coefficient values (Pedhazur, 1982). Table 4 reveals that for skill development, high perceived competence gymnasts scored higher (M = 4.47) than did the low group (M = 4.03). Also, high perceived competence gymnasts rated the team aspects of gymnastics as more important for their involvement (M = 4.21) than did the low competence group (M = 3.52).
Agbuga 2009	TAGM	Originally piloted in undergraduate students, piloted for reading only with appropriate sample	After some modifications, the results indicated that all indices (χ2/df = 1.09, CFI = .99, NNFI = .98, and RMSEA = .02) represented an excellent fit between the three-factor model and the data, with factor loadings ranging from .40 to .84.
Xiang 2004	TEOSQ	Based on scales used in different population	
**Physical Domain**			
MANUAL: The ALPHA project. ALPHA Fitness Test Battery for Children and Adolescents. 2009	ALPHA	Fitness tests were selected for the battery as part of the APLHA project. A large scale review of the literature (Ruiz 2009; 2010) and the ALPHA consortium consisting of field experts.	

Hoeboer 2016	AST 1/2	Pilot study conducted (54 children), tools developed in collaboration with PE teachers	AST-1<br>Welch's F (4,21.011) = 22.968, p < 0.05<br>AST-2<br>Welch's F<br>(4,20.366) = 27.746, p < 0.05<br>Differences between KTK categories were all significant<br>(P < 0.05) for AST-1 and AST-2 except for the difference between Normal Motor Giftedness and High Motor Giftedness<br>AST-1 Mean Difference Normal Motor Giftedness and High Motor Giftedness:<br>9.252 (0.107–19.57)<br>AST-2 Mean Difference<br>Normal Motor Giftedness and High Motor Giftedness 1.392 (–9.62–12.40)<br>AST-1 Mean Difference<br>Good Motor Giftedness and High Motor Giftedness<br>–2.693 (–7.91–13.30)<br>AST-2 Mean Difference Good Motor Giftedness and High Motor Giftedness<br>– 5.193 (–16.50–6.12)	Overall, there was a low correlation between AST-1 and the KTK (r = –0.474, P < 0.01), and a moderate correlation between<br>AST-2 and the KTK (r = –0.502, P < 0.01). Overall, there was a low correlation between AST-1 and the KTK (r = –0.474, P < 0.01), and a moderate correlation between<br>AST-2 and the KTK (r = –0.502, P < 0.01) The correlations between AST-1 and KTK were higher when split<br>up for gender (girls: r = –0.501, P < 0.01; boys: r = –0.533,<br>P < 0.01). For AST-2 the correlation between AST-2 and the<br>KTK was lower for girls (r = –0.448, P < 0.01) and higher for<br>boys (r = –0.566, P < 0.01) than the overall correlation. In<br>general, correlations were also higher when examined per<br>age group (see Appendices 1 and 2). With the exception of<br>the low correlation coefficient of the AST-2 in 7-year-olds (r = –<br>0.290, P < 0.01), the other correlation coefficients were near or far above 0.50 for each age group.
Deitz, Kartin and Kopp 2009	BOT-2 SF	Product survey and focus groups to identify the least effective items from the BOTMP and to identify new items. Participants included occupational therapists, physical therapists and developmental adaptive physical education teachers. Professional reviews of the BOTMP also consulted in the three step process, based on a synthesis of the three steps, six goals were developed and used to guide the revision. For new items, national try out and standardisation was preceded by pilot stage		(adj r = .80); the correlation between the Fine Motor composite on the<br>BOTMP and Fine Manual Control on the BOT-2 was moderate (adj r =.60); the correlations between the BOTMP Gross Motor composite and<br>BOT-2 Body Coordination, Strength and Agility (with knee push-ups),<br>and Strength and Agility (with full push-ups) were moderate (adj rs =.59, .69, and .73, respectively)
Bruininks & Bruininks, 2006	BOT-2 SF		Strong support for the four motor area composites used to evaluate performance on the BOT-2" and that the four factor model resulted in significant improvement of fit over the two-factor model (gross/fine motor) used in the BOTMP	
Fransen 2014	BOT-2 SF		r = 0.44	
Hassan 2001	BOT-2 SF		Four factors accounting for 56.5% of variance<br>Factor loadings<br>0.30–0.90	
MANUAL: Council of Europe. SPORT TESTING PHYSICAL FITNESS EUROFIT. Experimental Battery, PROVISIONAL HANDBOOK. Mar 2011	EUROFIT	European Research Seminar series informed the selection of battery items. Research Seminars the Committee of Experts on Sports<br>Research approved the experimental complete Eurofit test battery for testing physical fitness.		
Manual: Plowman 2013	FITNESSGR AM	FITNESSGRAM® Scientific Advisory Board, multidisciplinary team who developed/ updated FITNESSGRAM, selected battery components. FITNESSGRAM® (FG) was implemented in phases with the		Beets and Pitetti (2006) and Mahar et al. (1997) provide excellent examples of determining the criterion referenced reliability of the FITNESSGRAM's PACER and 1-mile run items.

		first pilot conducted in 30 schools in the Tulsa, Oklahoma School District (1982-83) using the AAHPERD Youth Fitness Test (YFT) (American Alliance for Health, Physical Education, and Recreation [AAHPER], 1976; Lacy & Marshall, 1984; Razor, 1984). In the second year (1983-84), approximately 125 schools throughout Oklahoma participated and were able to select either the AAHPERD YFT or the AAHPERD Health Related Fitness Test (HRPFT) (American Alliance for Health, Physical Education, Recreation and Dance [AAHPERD], 1980). After these successes, FG was implemented on a national basis first as a pilot, one district per state in addition to OK (1984-85), and then unrestricted (1985-86).	Hartman and Looney (2003) provide similar procedures for the FITNESSGRAM's back-saver sit-and-reach test item. Saint-Romain and Mahar (2001) illustrate the criterion-referenced reliability of the push-up and modified pull-up.
Mahar and Rowe 1997			Pearson correlation between 1 mile run/walk test and both trials of PACER (-.59<r-.67<)
Barnett 2015	GSPA	Participants in the expert panel were sought via author contacts and comprised of two groups, i) 13 researchers (including Authors 1, 3, 4) and ii) six golfing experts/professionals identified and com-piled from a governing body for the sport in Australia 'Golf Australia' (Research and Development -Author 2, High Performance Director, Technical Director, Golf Development Director, Junior Development Manager and the Tournament Preparation Consultant). Inclusion criteria for researchers were to have published internationally and/or be currently conducting a PhD in the area of children's movement skill assessment. Delphi method was used to refine components. Rather than develop a new assessment approach potentially not compatible with established assessment models, we adopted the TGMD-2 format; a common process-oriented assessment of FMS competency in children	
Valentini 2014	MABC-2	A panel of experts formed by health-related professionals was established to test content validity of the MABC-2 for Brazilian children. Three experts (Ph.D. degree in Motor Development) were asked to use the following Likert scale to independently evaluate the level of agreement of each task regarding clarity and pertinence: (5) very clear/pertinent; (4) somewhat clear/pertinent; (3) neutral; (2) not really clear/pertinent; and (1) not at all clear/pertinent). Ten health-related professionals (physical therapists, kinesiologists, physical educators, and pediatricians) participated in the face validity procedure. Each evaluator used a Likert scale (5 points) to score all motor items regarding the appropriateness to measure motor impairment: (5) strongly agree; (4) agree; (3) neutral; (2) disagree; and (1) strongly disagree.	We also addressed criteria validity using two methodologies of predictive validity of the MABC-2. First, the discriminant analyses on the standard scores showed significant differences among children identified with DCD, at risk for DCD and TD children (F (2,841) = 722.07, $p < .0001$, $\eta 2 = .63$). The follow-up tests showed that the scores of TD children were significantly higher than the groups of children classified as at risk and with DCD (p-values < .0001). Moreover, the scores of children classified as at risk were significantly higher compared to the children with DCD (p-values < .0001). Table 5 provides successful prediction rates for membership in each variable (category) used in the discriminant analysis. Issue with reporting directly from MABC-2 subsets Pearson's correlations indicated a significant, positive and moderate relationship between TGMD-2 and MABC-2 standards scores ($r = .30$; $p < .02$). Correlations in each classification group, DCD ($r = .54$; $p = .08$), at risk for DCD ($r = .26$, $p = .20$) and TD

			($r = -.05$, $p = .40$) failed to reach significance, although the magnitude of correlation between MABC-2 and TGMD-2 scores for children with DCD increased. A related samples t-test revealed no significant difference between both instruments for the children in general (t (42) = 1.36, $p < .18$) or within each classification group (p values range from .16 to .31).
Kita 2016	MABC-2	We performed confirmatory factor analysis to examine the factorial validity of the AB2 of the MABC-2 using the SSs of the eight test items. This analysis tested a postulated model in which each of the three components (which contain a set number of constituent items, e.g., Manual Dexterity has 3 test items) were allowed to correlate with one another, but no correlated errors were permitted. We evaluated the appropriateness of our postulated model using maximum likelihood estimation techniques. Multiple fit indices were used to evaluate model fit: chi square values with p value, chi square statistic to Degree of Freedom ratio ($\chi 2/df$), the Goodness-of-Fit Index (GFI), the Adjusted Goodness-of-Fit Index (AGFI), the Comparative Fit Index (CFI), and the Root Mean Square Error of Approximation (RMSEA). We set the statistical criteria for a good fit between the model and data as follows: $p > 0.05$ for chi square values, $\chi 2/df < 5$ [9], GFI > 0.95, AGFI > 0.95, CFI > 0.95, and RMSEA < 0.05 [9], [10]. We examined each path in the model using Wald tests with the p-value set at 0.05. We applied the bootstrapping approach to the model to support the analysis, since the present study had a small number of participants. We generated 2000 bootstrapped samples and conducted analyses according to the Bollen–Stine procedure [11], in which the postulated model is corrected when the p value is not significant.<br>All fit indices indicated a good fit between the postulated model and the data ($\chi 2$ (17) = 12.685, $p = .757$; $\chi 2/df = 0.746$; GFI = .977; AGFI = .951; CFI = .999; RMSEA = .000). A bootstrapping analysis with the Bollen–Stine procedure using 2000 bootstrapped samples did not yield a significant result ($p = .817$). Thus, these results demonstrated high factorial validity of the AB2 in the Japanese samples.<br>The Wald test revealed significant factor loadings from three components into individual test items. All but one factor loading reached significant levels at $p < .001$ (path coefficients ranging from .49 to .59), and the loading from Manual Dexterity into Drawing Trail 2 was significant at $p < .05$ (path coefficient = .30) (Fig. 1). We also found significant correlations between Balance and the other two components (rs = .65 and 54, $p < .001$), while the correlation between Manual Dexterity and Aiming & Catching was not significant ($r = .15$, $p = .40$).<br>The average SSs obtained by the present sample were higher than those in the normative sample with respect to Manual Dexterity ($t(131) = 4.37$, $p = .000$) and Balance ($t(131) = 4.62$, $p = .000$), but not Aiming & Catching ($t(131) = 1.48$, $p = .14$). The SS of total score was also higher in the present sample compared with the normative sample ($t(131) = 3.38$, $p = .001$). These results suggest that our sample of Japanese children tended to have higher scores on the AB2 of the MABC-2 compared with the group of normative children (i.e., UK children).<br>Moreover, girls scored higher than boys in Manual Dexterity ($t(130) = 2.06$, $p = .041$) and Balance ($t(130) = 4.34$, $p = .000$) while there were no significant gender differences in Aiming & Catching ($t(130) = 1.57$, $p = .119$) (Fig. 2). Along with the high scores on the two above-mentioned components, girls also obtained	

		a higher SS of total score compared with boys ($t(130) = 2.41$, $p = .017$).
Wagner, 2011	MABC-2	To verify the factorial validity of M-ABC-2 (AB2), a confirmatory factor analysis (CFA) was conducted using AMOS 18 (Arbuckle, 2003). The Mardia-Test was used for the assessment of multivariate normal distribution. One factor loading each was fixed to one in order to scale the latent variables (Fig. 1). The non-normalized model parameters were estimated by maximum likelihood using the covariance matrix. Standardized partial regression weights and error variances (Table A.1), the intercorrelation matrix (Table A.2) as well as relevant descriptive characteristics of all subtests (Table A.3) are listed in Appendix A. To evaluate the model fit, selected incremental fit indices were referenced, including root-mean-square error of approximation (RMSEA), standardized root mean residual (SRMR) and comparative fit index (CFI). The selection of the indices is oriented on Beauducel and Wittmann (2005); their evaluation is based on the recommendations of Hu and Bentler (1999). Despite a good global fit, structural equation models can still be problematic within their sub-structures. Therefore, the model's local fit was assessed more comprehensively in a second step where convergent and discriminant measures were evaluated. The convergent measures assessed were indicator reliability (IR) and factor reliability (FR) as well as the average assessed variance (AAV). Fornell–Larcker Ratios (FLR) and χ^2-difference tests (CMIN) were used as discriminant measures. The selection and evaluation of the convergent and discriminant measures was based on previous recommendations (Homburg, Klarmann, & Pflesser, 2008). Modification indices were not included in the analysis because the CFA was intentionally used for model verification and not for exploratory model optimization. Finally, verification of an equivalent hierarchical model was not performed because the total impairment score does not represent a theoretically deduced motor construct.<br>The Bollen–Stine corrected χ^2-statistic revealed no significant differences between the theoretical and the empirical covariance matrix ($\chi^2 = 28.675$; $df = 17$; Bollen–Stine p value = .318). The incremental fit indices (RMSEA = 0.046 [0.011; 0.075], SRMR = 0.038; CFI = 0.960) supported this finding because they were below the upper limit of RMSEA (<0.08) and SRMR (<0.11) and above the lower limit of CFI (>0.95). In conclusion, the incremental fit indices provided evidence for the factorial validity of M-ABC-2 (AB2).<br><br>Sub-structure analyses<br>For convergent measures, each estimated factor loading differed significantly from zero ($t \geq 1.645$). However, factor reliability of latent variables AC and BL was well below the minimum requirement of $r \geq 0.60$. In addition, an average of less than 50% of the total variance of each indicator block was explained by the superordinated latent variables MD, AC and BL. Hence, the latent variables only showed limited explanatory power for the reflective indicators or, in other words, the sum of all indicators showed insufficient measurement accuracy for the superordinated latent variables. Indicators drawing trail 2 (MD3), two-hand-catch (AC1), walking heel-to-toe forwards (BL2), and especially hopping on mats 2 (BL3) are particularly unreliable because less than 40% of the variance of these indicators was explained by the superordinated latent variables.

Hermman 2015	MOBAK-3	In order to ensure the content validity of the test items, we invited seven experts (The experts recruit from University of Basel (Switzerland, Department of Sport, Exercise and Health), University of Potsdam (Germany, Faculty of Human Sciences) and University of Teacher Education (Northwestern Switzerland) on early-childhood (physical) education, sports motor skills, psychometrics, and sports pedagogy to participate in the discussion. This process of developing the test items may be understood as a design step for maintaining curricular and content validity.	For the first step, we divided the total sample (n = 317) randomly into two subsamples. The exploratory factor analyses were based on the data from the first subsample (n = 158), while the confirmatory factor analysis was based on the second subsample (n = 159). The exploratory factor analysis with the oblique GEOMIN rotation for one to four factors was calculated with the 10 performed test items in model 1a. The factor loading and residual variance were released for estimation and cross loadings were allowed. For the subsequent analysis, we only took items into account that possessed a sufficiently large (≥ .40) significant loading on only one factor. The same analysis with a shortened test battery of eight tests items was calculated in model 1b.<br>In the second step, we tested the resulting structure with eight test items against restrictive conditions of the confirmatory factor analysis on the basis of the data of the second subsample (model 2a). In this confirmatory factor analysis, cross loadings were not allowed. Factor loadings and residual variance were estimated freely for each test item. Likewise, we repeated this confirmatory factor analysis on the data of the total sample (model 2b). There is a further restriction in model 2c, where the factor loading of the test items for each factor is equated within the confirmatory factor analysis (essential tau-equivalent loading; Bühner, 2011, p. 400). With this, one can examine whether a balance of the factor loading and thus the formation of a factor sum value about a test item of one factor is permitted. Subsequently, the factor sum values of the two factors are calculated (with the non-estimated sample). We conducted a univariate analysis of variance to examine potential differences between boys and girls.<br>In the third step, the measurement invariance testing was examined, which indicates equality of measurement parameters. Missing measurement invariances would hint at a presence of a differential item functioning (DIF) and would thus restrict the validity of the results on gender differences (Dimitrov, 2006). In accordance with Geiser (2011) and Dimitrov, (2006), we realized a sequential procedure in order to examine the measurement invariance of the model concerning gender. This involved taking two steps with increasingly stringent nested models: configural invariance and factorial invariance.<br>The development of the configural model started with the specification and the examination of two independent confirmatory factor analyses for boys and girls, respectively. These gender-specific models 3a were called baseline models.<br>We examined the configural invariance in model 3b by combining this gender-specific model in a multiple group model. This allowed for a model test for boys and girls simultaneously and revealed the baseline comparison values for the subsequent model 3c with factorial invariance. All parameters were estimated freely. Only the factor structure was equated between boys and girls (Dimitrov, 2006; Geiser, 2011; Widaman & Reise, 1997). This was done in order to make sure that the factor structure (amount and type of latent factors and loadings) was the same for boys and girls.<br>Subsequently, we measured the factorial invariance in model 3c, which was based on the configural invariance and restricted the relation between the items and the latent factors. We equated additional factor loadings concerning the boys and girls in the model by constraining the non-standardized factor loading in model 3b above boys and girls invariantly (Dimitrov, 2006; Geiser, 2011; Widaman & Reise, 1997). The tests of gender-specific differences between the inter-correlation of the latent factors follow the Wald Test of Parameter Constraints.

			As model 3b and model 3c were nested, the models could be compared using a difference test. We performed this with the Mplus module chi-square difference testing for WLSMV, as the classical chi2-difference tests was not permitted due to the WLSMV estimations (Muthén & Muthén, 2012). Furthermore, we used the model test to examine whether the intercorrelation between the latent variable was significantly different for boys and girls.<br>Finally, in model 4, we conducted a confirmatory factor analysis with the covariate gender (MIMIC) based on model 2b in order to be able to estimate the differences in the latent factors between boys and girls. Additionally, we requested the modification indices (MI > 10) for the direct effect of the covariate gender in the manifest variables of the test items in order to test for DIF.<br>The evaluation of the goodness-of-fit of the models followed the fit indices suggested by the literature (Bühner, 2011; Hu & Bentler, 1999; Schermelleh-Engel, Moosbrugger, & Müller, 2003; Schreiber, Nora, Stage, Barlow, & King, 2006). The following cut-offs hinted at a very good model adjustment: comparative fit index (CFI) > .95, root mean square error of approximation (RMSEA) > .06, standardized root mean residual (SRMR) < .11 (only exploratory factor analysis), weighted root mean square residual (WRMR) < .90. As the chi2 value was not interpretable without further calculation due to the WLSMV estimators, this was not specified in the fit indices. Thus, only standardized coefficients were reported continuously due to the better interpretability of the results.<br>CFI = 0.80, RMSEA = 0.076 (.048-.104), SRMR = 0.13<br>Factor loadings = ± 0.006–0.786<br>Two-Factor (10 items)<br>CFI = 0.96, RMSEA = 0.038 (.000-.077), SRMR = 0.09<br>Factor loadings = ± 0.001–0.787<br>Two-Factor (8 items)<br>CFI = 0.94, RMSEA = 0.056 (.000-.104), SRMR = 0.08<br>Factor loadings<br>NR<br>Confirmatory Factor Analysis<br>Two-Factor (8 items)<br>CFI = 0.96, RMSEA = 0.036 (.000-.082), WRMR = 0.65<br>Factor loadings<br>NR<br>Two-Factor (8 items; Total Sample)<br>CFI = 0.98, RMSEA = 0.024 (.000-.057), WRMR = 0.65<br>Factor loadings = 0.46–0.73
Hermman 2017a	MOBAK-3	In the MOBAK-1 pilot study, 317 first graders (n = 143 boys, n = 174 girls; M = 7.0 years, standard deviation [SD] = 0.36) were tested in Zurich (Switzerland).	We calculated an exploratory structural equation model (CFI = 0.99; RMSEA = 0.024) and a confirmatory factor analysis (CFI = 0.95; RMSEA = 0.041) with the eight MOBAK-5 test items. Both analyses confirm that the MOBAK-5 test instrument has the same two-factor structure with the factors self-movement and object movement as that found in previous studies for the analogously constructed MOBAK-1 and MOBAK-3 test instruments.<br>Model 1: The exploratory structural equation model achieved a good model fit (χ2 = 16.40; df = 13; p = 0.228; CFI = 0.99; RMSEA = 0.024).<br>Model 2: The test of the two-factor structure by means of confirmatory factor analysis showed a satisfactory model fit (χ2 = 33.23; df = 19; p = 0.023; CFI =

		0.95; RMSEA = 0.041). The factor loadings ranged between 0.26 and 0.77. The correlation between the two factors was r = 0.53<br>Model 3a. In the next step, we added the covariates gender, age, and BMI to the confirmatory factor analysis<br>The model achieved a fit that was still satisfactory ($\chi2$ = 72.12; df = 37; p < 0.001; CFI = 0.90; RMSEA = 0.047).<br>Model 3b: we added the covariates frequency of team sports and frequency of individual sports to the confirmatory factor analysis<br>The model fit was good ($\chi2$ = 52.00; df = 31; p = 0.011; CFI = 0.94; RMSEA = 0.039).
Hermman 2016	MOBAK-3	The two-factorial structure with CFA (Model 1a) resulted in good model fit ($\chi2$ = 27.56; df = 19; p = .09; CFI = .97; TLI = .96; RMSEA = .037). The factor loadings in Model 1a were within the acceptable range, with values between .42 and .71. The inter-correlation of the two factors was r = .55. Hence, our CFA confirmed the theoretically assumed two-factorial structure of the MOBAK-3 test items.<br>In Model 1b, we calculated a CFA with essential tau-equivalent loadings using ML-estimation. This model also showed good model fit ($\chi2$ = 30.8; df = 25; p = .19, CFI = .97; TLI = 97; RMSEA = .027). The calculation of a sum value within each factor was statistically acceptable and achieved a maximum of eight points (four items with two points each).<br>We calculated ICCs for these two factor sum values. The ICCs were low for the factors Object Control (.036) and Locomotion (.013). It may therefore be concluded that the total variance is attributable almost entirely to the variance between the students. Only 3.6 % and 1.3 % of the total variance, respectively, can be explained by membership in a class. Altogether, there were highly significant differences between the five latent classes, with large effect sizes on the factor sum values for Object Control (F = 207.59, p < .001, $\eta2$ = .723) and Locomotion (F = 85.86, p < .001, $\eta2$ = .519).<br>Furthermore, the distribution of sex was significantly different between the classes (F = 52.65, p < .001, φ = .404). The types of sport the students participated in and the extent of their physical activity in organized sports also differed significantly between the classes (F = 207.59, p < .001, φ = .723 and F = 85.86, p < .001, $\eta2$ = .519, respectively). However, the classes did not differ with regard to age (F = .599, p = .66, $\eta2$ = .007; cf. Table 6).
Carcamo 2020	MOBAK-3	CFA for the MOBAK 1-2 test instrument showed a satisfactory model fit ($\chi2$ =34.29; df = 19; p = 0.017; CFI = 0.962; RMSEA = 0.038), the factor loadings range from 0.34 to 0.66 and the correlation between the two factors is r = 0.77, all of which are statistically significant. To confirm the two factor structure of the MOBAK 3-4 test instrument, the results of the CFA show the following fit indices: $\chi2$ = 36.70; df = 19; p = 0.009; CFI = 0.892; RMSEA = 0.043. The factor loadings are in a range between 0.38 and 0.62, and the correlation between the two factors is r = 0.67, all of which are statistically significant (Graph 2). The CFI approaches but does not achieve the conventional

			limit of .90, but the RMSEA absolute fit index is within the established boundaries, meaning that the model can be accepted for the MOBAK 3-4 test instruments. In relation to the confirmation of the two factor structure for the MOBAK 5-6 test instrument, the results of the CFA show satisfactory fit indices (χ2 = 55.48; df = 19; p = 0.001; CFI = 0.926; RMSEA = 0.051. The factor loadings range between 0.29 and 0.68 and the correlation between the two factors is r = 0.61, all of which are statistically significant. For the factorial validity of Model 2, which considers the gender, BMI, and age as covariates, the CFA results for the MOBAK 1-2 test instrument show satisfactory fit indices (χ2 = 49.05; df = 37; p = 0.089; CFI = 0.929; RMSEA = 0.034). Gender (binary coding: girls = 1, boys = 2) has a small relationship with object movement, where boys have better results than girls. BMI has a small negative relationship with object movement and a weak negative relationship with self-movement. Children with a low BMI had higher values. There is also a small relationship with age in both object movement and self-movement with older children scoring higher. With regards to the CFA of Model 2 with the MOBAK 3-4 test instrument, the results of the indices of fit are: χ2 =62.47; df = 37; p = 0.006; CFI = 0.88; RMSEA = 0.041. The CFI index is slightly below the recommended level of fit but the RMSEA index is within the standard boundary for the model to be acceptable. Gender has a moderate relationship with object movement and a small negative relationship with self-movement. Boys have higher values than girls for object movement, while girls have higher values for self-movement. BMI has a small negative relationship with object movement and a moderate negative relationship with self-movement. Children with a low BMI had higher values. The indices of fit from the CFA of Model 2 in the MOBAK 5-6 test instrument are as follows: χ2 = 90.09; df = 37; p < 0.001; CFI = 0.89; RMSEA = 0.048. The CFI index is very close to the recommended boundary but the RMSEA index is within the standard boundary for the model to be acceptable
Manual: Hermman 2015	MOBAK-3	The test items for the acquisition of basic motor qualifications were developed on the basis of normative pedagogical discussions. The main question was: What should a child at a certain age be able to perform in order to take part in the culture of sports and exercise? The final arrangement of the test battery is the result of a wide selection of potential test items compiled and discussed in several expert discussions. In order to ensure curricular validity, we developed the test items in close connection with the learning goals specified in the curriculum. The criteria for item construction were gender specific test fairness as well as feasibility and age appropriate item design. The eight test items are explained in detail in the appendix. These test items measure eight basic motor qualifications which can be assigned to the two basic motor competencies “object movement” and “self-movement”. The assignment in the competence structure model was carried out on the one hand on the empirical level and on the other hand on the basis of the motor development theory.	A two-factor analysis has been explored through factor analysis and has been confirmed with good model fitting for the MOBAK-3 test battery. The first area, “self-movement [or: locomotion],” includes the four test items “balancing,” “rolling,” “rope skipping,” and “moving variably.” The second area, “object movement [or: object control],” includes the four test items “bouncing,” “dribbling,” “throwing & catching,” and “throwing.” Furthermore, it has been statistically verified that the calculation of a factor sum value (= sum of all items of an area) is acceptable for the calculation of a total value for each MOBAK area
Ericsson 2008	MUGI		A factor analysis of the MUGI observation checklist was carried out based on motor skills observations of all pupils in the first and second school years in Sweden (n=245). The factor analysis was exploratory and the nine items were grouped into two components.

			Factor loadings 0.56–0.80. All variables have a factor weight of between 0.6 and 0.0. This means that all tasks included have relevance in capturing the two different aspects of gross motor capability	
Zuvela, 2011	POLYGON	The first phase included the construction of the 24 tasks for fundamental movement skills assessment. Tasks were chosen according to their use in the PE program. For that purpose a pilot testing was conducted among 10 children aged 8 years to verify the practical realization of the tasks, correct any possible shortcomings and, most important, make sure all the tasks were equally timed. After those corrections and verifications ninety-five pupils were tested for 24 new fundamental movement skills tasks. Every task was repeated three times to assess reliability. After selecting a battery of four tasks that best represent a certain movement skills area, the investigation entered into the second phase by constructing the new fundamental movement skills assessment tool - polygon (FMS-POLYGON). A definition of polygon connotes a successive execution of a certain number of tasks in a shortest time possible. A pilot testing was conducted among 20 children aged 8 years to assure the practical realization of the test and correct any possible limitations. After that, a representative sample of ninety-five pupils was tested for a new FMS-POLYGON. Every participant saw the demonstration of the FMS-POLYGON and then repeated it four times (with first time being the practice one, with no time recording) to assess the intra-rater reliability of the test. The third phase of the investigation included an assessment of the fundamental movement skills by a validated test - TGMD-2. As recommended (Ulrich, 2000) all participants were videotaped and their level of FMS assessed by a single examiner.	The four factorial analyses, with the principle component method and varimax rotation were used to define the most appropriate task for each of the FMS subgroups defined by Mrakovic. The most appropriate task for the newly constructed polygon test should be the one in which characteristic factor values (lambda) and percentage of the common variance derived the most of variance explained (variance percentage) for each subsample group. The factors were considered significant when explained variance (lambda) exceeded 1. In this way the task with the highest projection on latent dimension were chosen to be part of the newly constructed FMS test. According to the factorial analysis, in each movement area a single factor was extracted. Tossing and catching a volleyball against the wall had the highest projection in the object control skills area (0.84). Running across obstacles had the highest projection in the surmounting obstacles skills area (0.87). Carrying the medicine balls had the highest projection in the resistance overcoming skills area (0.86) and straight running had the highest projection in the space covering skills area (0.83). According to the results obtained, those tasks were inserted in the	ICC for the FMS-POLYGON showed a very high result (0.98) and confirmed the test's reliability (Table 2). Mean score for the FMS-POLYGON was 24.20 while the mean score for the TGMD-2 was 59.45. Correlation analysis between the newly constructed FMS-POLYGON and the TGMD-2 revealed the coefficient of -0.82 ($p < 0.05$) which indicates a high positive correlation because lower performance time in FMS-POLYGON test means better result, unlike the TGMD-2.
Myers 2015	PARAGON		Convergent validity information only provided on PA levels not gardening motions	
Rudd 2015	SS	The development of the postural control test protocols was guided by the Delphi approach<br>Four experts (three academic experts in human movement and skill acquisition and one physical education teacher) identified movement skills demanding postural control. Due to the relationship between superior postural control and gymnastics [21], the experts also reviewed 32 gymnastics skills (taken from the Gym Mix gymnastics for all national program) for potential inclusion in the postural control assessment tool. These skills were then ranked according to the demands they place on the two subdomains of the postural control system and the method by which this could be assessed. In the first iteration, nine skills were identified: cartwheel, handstand, arabesque (a body position in which one stands on one leg with the other leg extended behind the body, both legs should be held straight), forward roll, backward roll, rock (a training method for the forward roll), front support, back support (a static wedge shape with arms straight and legs straight and together) and log roll (a sideways roll with arm and legs straight and slightly raised off the ground). The second iteration assessed the feasibility of the skills as an assessment tool in a school setting, resulting in four skills being deemed unsuitable because of	Confirmatory factor analysis (CFA) in AMOS 22 was used to examine the factorial structure of the three stability skills and if they loaded onto a single construct, named stability skills. CFA was conducted with the maximum likelihood method of estimation. In order to specify a model containing latent variables for all factors, error variance was set at zero. Several goodness of fit measures were used to describe the models. In addition to the Chi square ($\chi 2$) statistic, which is influenced by sample size and as such can be unreliable [26], the following fit indices were considered: Chi square/DF (X2/DF); Comparative fit index (CFI) [27]; Root mean square error of approximation [28]; Standardised root mean residual (SRMR) [29]; and the PCLOSE [30].<br><br>The $\chi 2$ statistic is a measure of overall fit of the model to the data with a non-significant P-value ($P > .05$) indicating a good fit. Also, $\chi 2$ divided by the degrees of freedom ($\chi 2/df$) provides an indicator of fit with values of < 2 considered adequate fit. CFI values of .90 or above indicate an adequate fit. RMSEA values of .06 or lower and SRMR values of .08 or lower indicate a close fit when these statistics are taken together. Finally, the PCLOSE should be non-significant ($p > .05$) [31,27].<br><br>Confirmatory Factor Analysis	Predictive Validity<br>Individual stability skills and total mean scores and standard deviations are reported in Table 1 for each of the four cohorts separately. ANCOVA for summed stability skills controlling for BMI and grip-strength showed a significant main effect ($F (3.333) = 61.56$; $p = .001$; $\eta 2 = .36$). Post hoc comparisons revealed that all cohorts performed as expected with gymnasts (mixed SES) having superior stability skills than all other groups. Children from the high SES school scored better than the children from the mid and low SES schools and the children from the mid SES school scored better than the children from the low SES school.

		safety concerns (cartwheel, handstand, forward roll and backward roll) and one skill (arabesque) being similar to the YBT-LQ single leg balance. This left four skills: rock, log roll, front support and back support. The front and back support are very similar skills so it was decided only one needed to be included. We selected the back support task as it was reasoned that it would be more challenging due to it being a more unnatural position for the body to hold and therefore would require higher torso strength and postural stability.<br>As each of these skills measure different aspects of postural control, i.e. the rock has high orientation demands, the back support requires high whole body stability and the log roll requires both postural control and stability, it was believed that when combined they would provide a holistic picture of participants' postural control ability and as such be a good measure for the stability skills construct. Following this, nine experts (five academics, two physical education teachers and two state level gymnastics coaches) were invited to assess the skill components. To be included on the expert panel researchers had to have published papers internationally in the areas within or related to movement sciences; teachers had to have taught physical education or coached gymnastics to primary school aged school children; and gymnastic coaches needed to have advanced coach accreditation and be currently coaching. Using email, each panel member was provided with the assessment elements and procedure of the rock, log roll and back support and were asked to examine whether the identified components were the key elements for successful skill execution and to rank each of the two postural control demands (orientation and stability) of each skill on a Likert scale (1 = low; 5 = high). All panel members provided extensive feedback which centered around three themes: 1) the wording of the components was overly scientific for mainstream use; 2) separate components overlapped in the same skill; 3) two of the three skills were deemed to be eliciting low levels of postural orientation or stability demands. Based on this feedback a number of changes were made.	χ2(2) = 1.03; CFI = 1.00; SRMR = 0.02; RMSEA = 0.01<br>Back support: r = 0.60<br>Rock: r = 0.59<br>Logroll: r = 0.59<br>Three-factor model<br>χ2(85) = 145.7; CFI = 0.91; SRMR = 0.06; RMSEA = 0.05<br>Factor Loadings:<br>r = 0.76–0.88	
Examiners manual Ulrich 2019	TGMD-3	In developing TGMD-3 we merged recommendations made by test reviewers and users for improving the test with our own ideas of improving the test. As a result we have made 10 following improvement (more info in manual pg. 45 onwards). Content experts (n=3) with a minimum of (a) 18 semester hours of credit in motor development beyond a master's degree, (b) 3 years of experience teaching PE, (c)3 years of experiencing observing and evaluating children's gross motor development	Construct identification validity pg. 61 onwards (exploratory factor analysis and confirmatory factor analysis) RMSEA .056<br>Locomotor r= 0.56-0.90<br>Ball skills r=0.62-0.85	Correlation with criterion measures (MABC-2) pg. 56 onwards.
Webster and Ulrich 2017	TGMD-3	Item discrimination<br>Locomotor<br>r = 0.52<br>Ball skills<br>r = 0.54<br>Total<br>r = 0.53	Skill levels and age<br>Ball skills<br>r = 0.474<br>Locomotor<br>r = 0.391<br>Total<br>r = 0.448<br>Exploratory Factor Analysis<br>χ2 (65) = 520.25, p <.001<br>Factor loadings<br>0.797–0.897<br>Two-factor EFA explored, but findings did not support structure	

			Confirmatory factor analysis<br>One Factor<br>χ^2 (65) = 327.61, p <.001,<br>CFI =.95, TLI =.94, RMSEA =.10, SRMR =.03<br>Factor Loadings<br>0.76–0.92<br>Two Factor model also calculated, but not supported as strongly as one factor model	
Wagner, 2017	TGMD-3		Skills increased with age<br>Locomotor skills<br>β =.64, t(186) =<br>11.41, p <.001, R^2 =.41, F(1, 186) = 130.24, p <.001<br>Ball skills: β =.79, t(186) = 17.86, p <.001, R^2 =.63, F(1, 186) = 318.88, p <.001<br>Skills were better performed by boys than girls<br>F(1,147) = 8.09, df = 1, p <.01, η^2 =.041<br>Confirmatory factor analysis<br>Two-factor model<br>χ^2(53) = 71.31, Bollen–Stine p =.164, χ^2/df = 1.35, CFI =.98, RMSEA =.04, 90% CI [.01,.07], SRMR =.04<br>Divergent<br>TGMD-3 Locomotor and MABC-2 Balance<br>rs = 0.33<br>TGMD-3 Locomotor and MABC-2 Aiming and Catching<br>rs = 0.22<br>TGMD-3 Ball Skills and MABC-2 Balance<br>rs = 0.25<br>TGMD-3 Ball Skills and MABC-2 Aiming and Catching<br>rs = 0.30	Ball Skills and Throwing Distance<br>rs = 0.36<br>Locomotor Skills and Sprint Time<br>rs = 0.15<br>Predictive<br>Ball Skills and Throwing Distance (12 months later)<br>rs = 0.39<br>Locomotor Skills and Sprint Time (12 months later)<br>rs = 0.08
Temple 2017	TGMD-3		Third grade boys had higher ball skills than girls<br>(t = 9.45, df = 275, p <.001, d = 1.14)<br>Fourth grade boys had higher ball skills than girls<br>(t = 9.28, df = 275, p <.001, d = 1.12)<br>Locomotor Skills Mean Difference Between Boys and Girls-Grade 3<br>t = 2.68, df = 275, p =.008, d =.32<br>Grade 4<br>t = 1.67, df = 275, p =.096, d =.20<br>Boys had higher overall skill scores than girls in Grade 3<br>t = 5.24, df = 275, p <.001, d =.63 and Grade 4 t = 5.69, df = 275, p <.001, d =.69	
**Cognitive Domain**				
Economos et al., 2010	BONES PAS	Focus groups were held with 6-9 year old children, the literature was reviewed, and physical education specialists were consulted to identify common weight-bearing activities that children engage in on a regular basis. Research staff also directly observed children playing in after school settings on multiple occasions during the pilot phase of the BONES intervention. The need to quantify weight-bearing physical activity was balanced against the cognitive limitations of children (i.e. short attention span, inability to accurately estimate time). Given the age of the target population, the picture-sort technique was chosen as an appropriate method for this population		
Manios et al 1999	PHKA			
Lakes & Hoyt 2004	RCS	Literature search and pilot testing reported	Showed weak discriminant validity between the Cognitive and Affective subscales—items on the subscales appeared to load on the same factor, and the subscales had a high correlation (*r* = .90). Items on the Physical/Motor subscale formed a more distinct factor	

	(*r*’s = .75 and .78 with the Cognitive and Affective subscales, respectively).
Lakes 2012	PRS (Person, Rater, Subscale) Analysis:<br>Person= 46% mean variance between occasion one and occasion two<br>Rater = 12% mean variance between occasion one and occasion two<br>Subscale= 3% mean variance between occasion one and occasion two<br>Person, Rater= 14% mean variance between occasion one and occasion two<br>Rater, Subscale = 1% mean variance between occasion one and occasion two<br>Person, Rater, Subscale= 13% mean variance between occasion one and occasion two

Achievement Goal scale for Youth Sports (AGSYS); Attitudes Towards Curriculum Physical Education (ATCPE);Attitudes Towards Outdoor play scale (ATOP); Adapted Behavioural Regulation in Exercise Questionnaire (BREQ); Children’s Attraction to Physical Activity Questionnaire (CAPA); Children’s Attitudes Towards Physical Activity (CATPA); Commitment to Physical Activity Scale (CPAS); Children and Youth Physical Self-Perception Profile (CY-PSPP); Motivational determinants of elementary school students' participation in physical activity (DPAPI); Enjoyment in Physical Education (EnjoyPE); Food, Health and Choices Questionnaire (FHC-Q); Feelings About Physical Movement (FAPM); Healthy Opportunities for Physical Activity and Nutrition Evaluation (HOP’N); Lunchtime Enjoyment of Activity and Play Questionnaire (LEAP); Momentary Assessment of Affect and Physical feeling states (MAAP);Motivational Orientation in Sport Scale (MOSS); Negative Attitudes Towards Physical Activity Scale (NAS); Physical Activity Beliefs and Motives (PABM); Physical Activity Enjoyment Scale (PACES); Physical activity and Healthy Food Efficacy (PAHFE); Positive Attitudes Towards Physical Activity Scale (PAS); Physical Activity Self-Efficacy Questionnaire (PASE); Physical Activity Self-Efficacy Scale (PASES); The Revised Perceived Locus of causality in physical Education (PLOC in PE); Perceived Motivational Climate in Sport Questionnaire (PMCS); Response to Challenge Scale (RCS);Self-Perception Profile for Children (SPPC); Trichotomous Achievement Goal Model (TAGM); Task and Ego Orientation in Sport Questionnaire (TEOSQ); ALPHA Fitness Battery (ALPHA); Athletic Skills Track (AST) ½; Bruininks–Oseretsky Test of Motor Proficiency (BOTMP-SF); Canadian Agility and Movement Skills Assessment (CAMSA); EUROFIT; FITNESSGRAM (FG); FG-COMPASS (FGCOMP); Golf Swing and Putt skill Assessment (GSPA); Motorische Basiskompetenzen in der 3 (MOBAK-3); Movement assessment battery for children-2 (MABC2); Motorisk Utveckling som Grund för Inlärning (MUGI); Obstacle Polygon (OP); PA Research and Assessment tool for Garden Observation (PARAGON); Slalom Movement Test (SMT); Star Excursion Balance Test (SEBT); Stability skill test (SS)Test of Gross Motor Development-3 (TGMD-3); The Leger 20m Shuttle Run test (20MSR); Y Balance Test (YBT); Beat Osteoporosis Now-Physical Activity Survey (BONES-PAS); Pupil Health Knowledge Assessment (PHKA); Response to Challenge (RCS); Canadian Assessment of Physical Literacy (CAPL-2); Passport for Life (PFL)
