# Supplementary material for: Assessments Related to the Physical, Affective and Cognitive Domains of Physical Literacy Amongst Children Aged 7–11.9 Years: A Systematic Review
Source: Sports Med Open. 2021 May 27;7:37. doi: 10.1186/s40798-021-00324-8 (PMC8160065; doi:10.1186/s40798-021-00324-8)
Supplement: Supplementary file 5 — Additional file 5. [file 40798_2021_324_MOESM5_ESM.pdf]

ONLINE RESOURCE 5 – Reliability data extracted from included studies

Assessments related to the physical, affective and cognitive domains of physical literacy among children aged 7-11.9 years: a systematic review

[redacted]

Online Resource 5 Reliability results of included assessments

	Assessment	Internal consistency	Test-retest reliability	Intra-rater reliability	Inter-rater reliability
**Explicit PL Assessments**					
Longmuir 2017	CAPL-2 (CAMSA)		Evidence for test–retest reliability for completion time was excellent across short (n=59; ICC=0.84; 95%CI: 0.74 to0.91) and long (n=16; ICC=0.82; 95%CI: 0.53 to 0.93) test intervals. Evidence for test–retest reliability for the skill score was moderate (n=44; ICC=0.46; 95%CI: 0.20 to 0.66) over a short interval but substantial (n=16; ICC=0.74; 95%CI: 0.42to 0.90) over a long interval. Although reliability evidence was moderate for the movement skill score measured over a short interval, there was no significant difference between the test(median 17 of 28 points; range 11–20) and retest (median 17;range 8–20) scores (n=59; 95%CI of difference: −0.2 to 0.9;p=0.22)	Evidence for intra-rater objectivity for the skill score was moderate (ICC=0.52) and excel-lent for completion time (ICC=0.996)	Objectivity was assessed across 104 attempts by 53 children (34% female). Evidence for inter-rater objectivity was substantial (ICC=0.69) for the skill score and excellent for the completion time (ICC=0.997)
Longmuir 2018c	CAPL-2 (PLKQ)		Test-retest reliability data were collected from two samples of children who were asked to complete the PLKQ twice. One sample (n = 31) attended a summer camp in eastern Ontario, and completed the initial PLKQ on 2 separate days, at an interval of 2 days. A second sample of children (n = 35) completed the final PLKQ (Additonal file 3), a more streamlined version, at their school in southwestern Ontario, on 2 occasions over a 1-week interval. Test-retest reliability of the PLKQ responses was assessed using Pearson correlation coefficients.<br><br>The test-retest correlation coefficients for the PLKQ total score, as well as for individual items on the PLKQ, are provided in Table 9. Reliability of the PLKQ total score was strong (r = 0.62 and 0.69 over the 2- and 7-day intervals, respectively). Adjusting the correlation by age did not alter the reliability (r = 0.60 and 0.70 over the 2- and 7-day intervals, respectively). Over a 2-day interval, the reliability of most individual items was substantial to excellent. Responses to questions about the meaning of “cardiorespiratory fitness”, a story about sport training and fitness, and how to get in better shape had moderate reliability. Item reliability over a 7-day interval was similar for most questions. Reliability was lower over a 7-day interval, compared to the 2-day interval, for questions asking about the use of safety gear during physical activity and the meaning of “healthy”.<br><br>Reliability for the question that asked children about the recommended amount of daily sedentary time was moderate over both the 2- and 7-day intervals (r = 0.33 and 0.34, respectively). Even though the proportion of children answering correctly was similar (21 and 20%, respectively), a paired comparison of responses was significantly different (p = 0.045) between the first and second trials over the 7-day interval. Over a 7-day		

			interval, 72% and 77% of children correctly identified the recommended daily physical activity (1st and 2nd trial, respectively) and the correlation was strong (r = 0.61). Over a 2-day interval, reliability was low (0.20) and may have been influenced by the content of the initial PLKQ, which asked separately about moderate to vigorous activity at school and total throughout the day.		
Boyer 2013	CAPL-2 Plank hold		test-retest (ICC = 0.63; CI = 0.46, 0.75)	Intrarater (ICC = 0.83; CI = 0.73, 0.90)	Interrater (ICC = 0.62; CI = 0.50, 0.75)
Lodeewyk 2017	PFL	Internal consistency reliability coefficients: living skills, .75 to .86; active participation, .61 to .87; movement skills, .64 to .75; fitness skills, .62 to .77	Test-retest reliability using the data from the three raters 1 week later revealed strong consistency (r = .72 to .89)	Test-retest reliability using the data from the three raters 1 week later revealed strong consistency (r = .72 to .89)	The intraclass correlation coefficient among three simultaneous raters using PFL rubrics for fitness and movement skills revealed good scoring agreement (.65 to .82)
Cairney 2018	PLAYfun				The ICC coefficient for the total score among seven assessors in our pilot sample of 10 children was very good (ICC, 0.87).
Stearns 2020	PLAYfun	Internal consistency reliability for the items in the PLAYfun and PLAYbasic tools were examined using Cronbach's alpha for each time point (October 2014, March 2015). The specific subscales scores were poor-to-good (α = .47–.82; only 1/8 of the scores are slightly below borderline).		For individual raters, the internal consistency was good for the overall PLAYfun (α = .83–.87), borderline for the PLAYbasic (α = .56–.65; only 1/4 of the scores are slightly below borderline), and poor-to-good across the subscales (α = .35–.83).	Inter-rater reliability statistics are presented in Table 3. For average measures (for use when two scores will be averaged), good-to-excellent inter-rater reliability was observed for the larger PLAYfun tool.<br><br>For the average scores across raters, the overall PLAYfun and PLAYbasic were good (α = .87) and borderline (α = .61–.62), respectively.
**Affective Domain**					
Cumming et al 2008	AGSYS	α=0.78 for mastery<br>α=0.88 for ego orientation<br>The subscale reliability coefficients exceeded .75 in all age groups	Stability of measured scores over time was assessed in an independent sample of 11 male and 12 female competitive figure skaters ranging in age from 9 to 14 years. The athletes completed the AGSYS and were then retested 1 week later. Each of the subscales demonstrated adequate stability with test–retest coefficients of .92 for Mastery and .95 for Ego scores.		
Bornholt & Piccolo, 2005	ASK-KIDS	Movement α =0.88<br>Individuality α=0.68<br>Belonging α=0.77			
Jones 1988	ATCPE	3-point scale α=0.68<br>5-point scale α=0.76		Pearson's correlation coefficients .79, .39, .45, .78	
Beyer et al., 2015	ATOP	Benefits α =0.79<br>Fears α =0.79			
Sebire et al., 2013	BREQ	Intrinsic α=0.77<br>identified α=0.71<br>introjected α=0.59<br>external α=0.71<br>autonomy α=0.72<br>competence α=0.82			

		relatedness α=0.81		
Brustad 1993	CAPA	Each of the five factor subscales demonstrated acceptable internal reliability, with alpha levels ranging from ,62 to .78.		
Brustad 1996		The CAPA subscales that demonstrated satisfactory internal consistency were liking of exercise (alpha = .74), liking of physical exertion (alpha = .74), and liking of games and sports (alpha = .70). When two items were deleted from the peer-acceptance-in-physical activity scale,' internal consistency was greatly enhanced (alpha = .72). The subscale assessing attraction to physical activity for health reasons did not attain a sufficient level of reliability (alpha = .44), and this construct was excluded from further analysis.		
Simon & Smoll 1974	CATPA	Reliability coefficients:<br>social =0.80<br>health and fitness =0.80<br>pursuit of vertigo =0.84<br>aesthetic =0.89<br>catharsis =0.84<br>ascetic =0.84		Pearson Product-Moment correlations:<br>social=0.55<br>health and fitness=0.44<br>pursuit of vertigo=0.49,<br>aesthetic=0.62<br>catharsis=0.44<br>ascetic=0.51
Schultz, Smolll & Wood 1981		Cronbach’s alphas for the raw data were all reasonably large, ranging from .80 to .88. For the reweighted and item-deleted reweighted data sets the changas in alphas were minimal, differing by plus or minus 0.03 units or less for each subdomain.		Zero-order correlations across grades were examined and canonical correlation analysis was performed. The adjacent grade correlations were low for most subdomains. Vertigo and aesthetic subdomains showed the highest values of *r*s of approximately .45, catharsis the least stable *r*s from 0.6 for boys to .34 for girls.
DeBate 2009	CPAS	All scales α=0.820<br>attitudes towards PA α=0.821<br>value of PA α=0.702<br>motivation to be physically active α=0.429<br>attitudes towards PA α=0.820<br>value of PA α=0.725<br>motivation to be physically active α=0.526		
Welk et al 1997	CY-PSPP	Cronbach’s alphas calculated in both original and modified versions of scale:<br>sport competence (original .77, modified .60)<br>physical conditioning (.83 .70)<br>body attractiveness (.88 .78)<br>physical strength (.89 .79)<br>physical self-worth (.90 .82)<br>global measure of self-esteem (.85 .75)<br>mean (.85 .75)	Test-retest reliability examines the stability of the responses over time. Intraclass correlation coefficients were generally high for both versions of the CY-PSPP. Test-retest reliability was slightly better for the girls with both versions of the CY-PSPP. When both sexes were combined, the average R value was .73 for both versions. Exceptionally low test-retest scores were found for Physical Self-Worth (R = SO) and SE (R = .30) for the boys using the original version. If comparisons were made with only the four CY-PSPP subdomains (Sport, Cond, Body, Strong), then the test-retest reliability would have been higher for the original (R = .78) than for the modified version (R = .73).<br><br>Intraclass correlations (*r*)<br>sport competence (original .80, modified .77)<br>physical conditioning (.76, .66)	

			body attractiveness (.81, .74)<br>physical strength (.76, .76)<br>physical self-worth (.68, .73)<br>global measure of self-esteem (.59, .73)<br>mean (.73, .73)	
Welk et al 2005				Intercorrelations factor loadings in the measurement model revealed no substantial areas of concern. Median loadings for the total sample, boys subsample and girls subsample were 0.69 (range 0.41–0.82), 0.67 (range 0.32–0.85), and 0.70 (range 0.43–0.83). Most latent factor intercorrelations were reasonably moderate although some (i.e. among PSW, GSW, and Body) are notably large. In summary, these findings suggest an adequate fit for the CY-PSPP measurement model to these data and reasonable psychometric attributes.<br>Intercorrelations among manifest and latent.<br>Attractive body adequacy 2 factor 0.518, 3 factor 0.533, 4 factor 0.592, 5 factor 0.759, 6 factor 0.672,<br>Sport/athletic competence 1 factor 0.600, 2 factor 0.642, 3 factor 0.639, 4 factor 0.678, 5 factor 0.550<br>Strength competence 1 factor 0.606, 2 factor 0.763, 4 factor 0.531, 5 factor 0.581, 6 factor 0.478<br>Physical condition adequacy 1 factor 0.655, 2 factor 0.744, 3 factor 0.564, 4 factor 0.683, 5 factor 0.574<br>Physical self-worth 1 factor 0.888, 2 factor 0.801, 3 factor 0.664, 4 factor 0.768, 6 factor 0.753<br>Global self-esteem 1 factor 0.805, 2 factor 0.663, 3 factor 0.550, 4 factor 0.657, 5 factor 0.911
Chen 2011	DPAPI	Composite reliability.<br>Attitude .81<br>Intention .79<br>Autonomy .60<br>Competence .68<br>Relatedness .71<br>Intrinsic motivation .66<br>Identified regulation .70<br>Introjected regulation .66<br>External regulation .50<br>Amotivation .72		
Shewmake 2015	EnjoyPE	Enjoyment α=0.79, Perceived exertion α=0.60		
Gray et al 2016	FHC-Q	Self-determination α =0.87<br>outcome expectations α =0.82<br>self-efficacy α =0.90<br>habit strength α =0.79<br>goal intention α =0.74	For psychosocial variables, only outcome expectations audience response system (ARS) vs pen and pencil achieved 0.85 (Threshold >.70). all other variables, at either ARS vs pen and pencil or ARS vs ARS did not achieve this	

			ARS vs PP<br>Self-determination α =0.56<br>outcome expectations α =0.85<br>self-efficacy α =0.59<br>habit strength α =0.36<br>goal intention α =0.55<br>ARS vs ARS<br>Self-determination α =0.60<br>outcome expectations α =0.68<br>self-efficacy α =0.67<br>habit strength α =0.65<br>goal intention α =0.51
Bornholt 2005	FAPM	feeling ok α=0.69,<br>guilt α=0.79<br>worry α=0.73<br>anger α=0.80<br>disgust α=0.84	
Rosenkranz 2011	HOP’N	PA barriers self-efficacy α=0.64<br>PA enjoyment α=0.72<br>Perceived opportunity for PA α=0.49<br>Perceived parental support α=0.83	Test retest reliability median kappa agreement<br>Intrapersonal males .57 (.25-.73)<br>Intrapersonal females .61 (.26-.72)<br>Interpersonal males .48 (.43-.53)<br>Interpersonal females .29 (.22-.36)<br>Physical environment/policy males .61 (.47-.72)<br>Physical environment/policy females .58 (.38-.74)
Hyndman 2013	LEAP	Intrapersonal α=0.84 (retest α=0.88)<br>Interpersonal α=0.48 (retest α=0.45)<br>Physical environment/policy α=0.74 (retest α=0.74)	
Dunton 2014	MAAP	Positive affect α=0.87<br>Negative affect α=0.74	
Rose 2002	MOSS	American:<br>challenge α=0.81<br>curiosity α=0.61<br>mastery α=0.64<br>judgement α=0.64<br>criteria α=0.76<br>Australian:<br>challenge α=0.815<br>curiosity α=0.65<br>mastery α=0.76<br>judgement α=0.671<br>criteria α=0.75<br>British:<br>challenge α=0.81<br>curiosity α=0.62<br>mastery α=0.71<br>judgement α=0.63<br>criteria α=0.74	
Nelson 2009	NAS	α=0.82	
Dishman 2013	PABM		
Moore 2009	PACES	entire sample (α = .87, n = 511)<br>European American males (α = .87, n = 77)<br>females (α = .88, n = 80),<br>African American males (α = .86, n = 144),<br>females (α = .87, n = 165)	

Perry 2008	PAHFE	Decision-Making for Physical Activity Efficacy subscale yielded coefficient α=0.78 (pretest) and α=0.86 (posttest).<br>The PAHFE Decision-Making for Healthy Food Choices Efficacy subscale produced values of α=0.74 (pretest) and α=0.87 (posttest)	Test conditions not similar, some completed in home, some completed by school nurse. Spearman brown adjusted coefficent alphas Goal Setting PA pretest 0.92, posttest 0.96, Goal Setting healthy food pretest 0.94, posttest 0.96, Decision-Making for PA pretest 0.88, posttest 0.93, Decision-making for healthy food pretest 0.86, posttest 0.93,	
Nelson 2009	PAS	α=0.74		
Jago 2009	PASE	full scale PASE α=0.90 reduced scale α=0.81<br>sedentary tv α=0.83 reduced scale α=0.74<br>computer/video games α=0.85<br>r educed scale α=0.78<br>telephone α=0.84 reduced scale α=0.75		
Saunders 1997	PASES	Support seeking α=0.71<br>barriers α=0.72<br>positive alternative α=0.54<br>physical outcomes α= 0.46<br>social outcomes α=0.51	The test–retest correlation coefficients were 0.51 and 0.69, respectively.<br>Pearson correlation coefficient calculated:<br>social influences 0.78<br>self-efficacy; support seeking 0.76, barriers 0.82, positive alternatives 0.61<br>beliefs; physical outcomes 0.51, social outcomes 0.69, activity outcomes .51, social outcomes 0.58,	
Liang 2014	Physical Activity Self-efficacy, enjoyment, social support	Self-efficacy α=0.78<br>enjoyment α=0.90<br>social support from family α=0.86<br>social support from friends α=0.90	ICC: Physical Activity Self-efficacy=0.88, enjoyment=0.82, social support from family=0.86, social support from friends=0.91	
Vlachopoulos 2011	PLOC in PE	amotivation α=0.80<br>external regulation α=0.75<br>introjected regulation α=0.69<br>identified regulation α=0.83<br>intrinsic motivation α=0.81<br>perceived autonomy support α=0.81<br>vitality α=0.89		
Barnett 2015	PMCS	All α =0.60<br>Object Control α =0.72<br>Locomotor α =0.68	(Sample 1 completed test re-test reliability but this was outside the age range of the present systematic review)	
Lakes 2004	RCS			Lakes 2013 cites ICC = .92, .93, and .91 for the cognitive, affective, and motor scales, respectively from Lakes and Hoyt 2004
Lakes 2013				All ICC correlations (observer, teacher, parent) < 0.70
Lakes & Hoyt 2009			Test-retest reliabilities using conventional reliability measurements were .64, .84, and .80 for the Cognitive, Affective, and Physical scales, respectively; the test-retest reliability *g* coefficients (which consider multiple sources of error simultaneously and are always lower than measures based on a single source of error) were .37, .71, and .58 for the Cognitive, Affective, and Physical subscales, respectively. Although the test-retest reliability coefficients, measured using standard methods and compared to general standards for adequate test-retest reliability, were sufficient (.84 and	5 raters reported *g* coefficients for each of the three subscales of the RCS: .86 (Cognitive), .92 (Affective), and .88 (Physical/Motor)

			.80) for the Affective and Physical scales, the coefficient for the Cognitive scale (.64) was lower	
Leary 2011	Self-efficacy scale	The Self-efficacy Scale α =0.76		
Harter 1987	SPPC	cognitive α=0.76<br>social α=0.78<br>physical α=0.83<br>general α=0.73	Test re-test sample of 810 pupils retested after 9 months. These correlations, corrected for attenuation, were .78, .80, .87, and .70 for the Colorado sample, and .78, .75, .80, and .69 for the New York sample, for the four subscales	The correlations of the general selfworth subscale with each of the three competence subscales consistently tend to be among the highest, in the range between .40 and .58. The social and physical subscales are highly related across samples, within the range of .46 and .58. The relationship between the cognitive subscale and both the social and physical subscales tends to be lower, with some sample variation. Correlations among factors are of a similar magnitude, for example, general selfworth correlates with the three competence subscales .42-.53, social and physical correlate .56, and social and cognitive correlate .43
Byrne 1988				
Shevlin 2003		Time 1<br>Scholastic Competence α=0.736<br>Social Acceptance α=0.578<br>Athletic Competence α=0.532<br>Physical Appearance α=0.591<br>Behavioural Conduct α=0.653<br>Time 2<br>Scholastic Competence α=0.733<br>Social Acceptance α=0.629<br>Athletic Competence α=0.650<br>Physical Appearance α=0.723<br>Behavioural Conduct α=0.581<br>Time 3<br>Scholastic Competence α=0.656<br>Social Acceptance α=0.678<br>Athletic Competence α=0.645<br>Physical Appearance α=0.715<br>Behavioural Conduct α=0.644<br>Time 4<br>Scholastic Competence α=0.771<br>Social Acceptance α=0.639<br>Athletic Competence α=0.655<br>Physical Appearance α=0.601<br>Behavioural Conduct α=0.620		
Klint 1987		Success/status α=0.80<br>Affiliation-Friends α=0.70<br>Energy Release α=0.53<br>Affiliation-Team α=0.85<br>Excitement α=0.77<br>Fitness α=0.86		

Agbuga 2009	TAGM	Mastery Goals α=.74<br>Performance Approach α=.85<br>Performance Avoidance α=.71,			
Xiang 2004	TEOSQ	Master Goal Scale α = .88<br>Performance Goal Scale α = .84<br>Perception Performance of Mastery Focussed Climate α = .72<br>Perception Performance of Mastery Focussed Climate α = .79			
Moore 2009		Task subscale used, α = .90 in this sample			
**Physical Domain**					
España-Romero 2010	ALPHA		Significant inter-trials differences were found in stature for children (P < 0.001), in waist circumference for both children and adolescents (P = 0.001), and in the standing long jump test only for children (P < 0.05)/ Test and retest (hereafter called T1 and T2) were compared between boys and girls and between children and adolescents by means of two-way repeated measures analysis of variance (ANOVA), with sex and age group as fixed factors. Since no sex-specific effect on reliability of the studied physical fitness tests was found (all P>0.2), the analyses were performed for girls and boys together. However, age group-specific effect on reliability was found in three of the study tests (all P<0.05), therefore the analyses were performed for the whole sample as well as for children and adolescents separately.		Stature: P < 0.001<br>Waist circumference: P = 0.001<br>Standing long jump test: P < 0.05
Hoeboer 2016	AST 1/2		All measurements were conducted in a separate section of the gym by a team of four research assistants (fourth year PE students of the The Hague University of Applied Sciences) during regular PE lessons. They had been trained in conducting the tests according to the protocols in four meetings. The protocols provided guidelines about how to deal with adverse events, such as falling. The intraclass correlation coefficient between the first and second trial was 0.875 (95% CI [0.852–0.895).<br>Athletic Skills Track-1<br>ICC = 0.875 (0.852, 0.895)<br>Athletic Skills Track-2<br>ICC = 0.891 (0.870, 0.908)		
Deitz, Kartin and Kop, 2009	BOT-2 SF	Using the normative sample, internal consistency was examined using the stratified alpha method for each composite and the split-half method for each subtest. These were high (.93) for the Total Motor Composite for all age groups. For the Short Form, these were generally acceptable (.80) for all age groups, except for 4- and 8-year olds. For the subtests for individual age groups, internal consistency estimates of reliability were borderline to high. Correlations ranged from .60 to .92.	Test-retest reliability was examined for three age groupings of children (4 through 7-year olds [n = 43]; 8 through 12 year olds [n = 44]; 13 through 21-year olds [n = 47]) by administering the BOT-2 to the study participants on two occasions, separated by an interval ranging from 7 to 42 days. Pearson product moment correlation coefficients were .80 for the three age groups for the Total Motor Composite, the Short Form (with knee push-ups), and the Short Form(with full push-ups). Reliability coefficients for the three age groups for and three of the composites (Fine Manual Control,		Pearson product moment correlation coefficients were good for the Short Form and for all. Complete Form subtests and composites. With one exception (the Fine Motor Precision subtest, adj r = .86), they were all > .90

			Manual Coordination, and Body Coordination) and for their related subtests were highly variable, with 7 of the 9 correlations < .80 for the composites and 16 of the 18 correlations < .80 for the subtests. Also, especially for the Manual Coordination and Body Coordination Motor Area composites, a small practice effect was noted. The reliability coefficients for the three age groups for the remaining composites (Strength and Agility with knee push-ups; and Strength and Agility with full push-ups) and their related subtests were all >.80	
Bruininks RH. 1978. Manual	BOT-SF	inter-correlations between the full scale of 0.90 and 0.91		
Hassan, 2001	BOT-2 SF	Ball throw<br>r = 0.56<br>Jump up and clap<br>r = 0.58<br>Catch<br>r = 0.61<br>Heel-toe balance<br>r = 0.50<br>Standing balance<br>r = 0.55<br>Running<br>r = 0.66<br>Broad jump<br>r = 0.80		
Cepero 2011	EUROFIT			The test-retest intra observer reliability coefficient observed for physical fitness tests demonstrated a mean intraclass coefficient (ICC) of 0.87 (0.76–0.98)

Patterson 2001	FG	Teacher-Reported Cu. R = 89.<br>Single trial R = .80 for boys and.75 for girls for teacher-reported CD<br>Child reported CD was R= .70 for boys and .69 for girls.<br>One-way repeated measures ANOVA indicated no significant differences in CU performance across the 2 days for either boys, F (1, 35) =.004, p= .948, or girls, F (I, 47) = .01, p= .92.<br>Child-Reported Cu.<br>Test-retest reliability was R = .82 (95% CI = .66-.91) for boys and R= .81 (95% CI = .67-.90) for girls.<br>Single trial estimates of reliability were R = .70 (95% CI = .49-.84) for boys, while for girl's reliability was R= .69 (95% CI = .50-.81).	
Morrow, Martin and Jackson 2010	FITNESSGRAM	Reliability of the various types of repeated test administration<br>(% Agree, Mod. kappa, Phi, x(squared), (p <), n(a)<br>Teacher/ teacher<br>15-m PACER: .81 .62 .62 .001 32 20-m<br>PACER: .82 .64 .66 .001 256<br>BMI .97 .94 .92 .001 456<br>Curl-up .78 .56 .51 .001 467<br>Flexed arms hang .82 .64 .64 .001 67<br>One mile run .92 .84 .84 .001 12<br>Push-up .74 .48 .40 .001 396<br>Shoulder stretch .92 .84 .79 .001 458<br>sit-and-reach .89 .78 .67 .001 114<br>Expert/expert<br>Trunks lift .86 .72 .58 .001 450<br>20-m PACER .95 .90 .90 .001 139<br>BMI .96 .92 .90 .001 198<br>Curl-up .87 .74 .64 .001 199<br>Push-up .77 .54 .51 .001 202<br>Shoulder stretch .96 .92 .90 .001 194<br>sit-and-reach .86 .72 .67 .001 91<br>Trunks lift .78 .56 .57 .001 197	Teacher reliabilities are adequate or acceptable for all fitness items, based on percent agreements, modified kappa, phi coefficients, and significant chi squares<br><br>Teacher Vs Expert groups:<br>% agreements were generally good, modified kappas generally indicated moderate to substantial agreement and phi and chi square results were significant. Teacher groups: represent reliability) based on percentage of agreement were generally highest with body mass index (BMI; rxx' $\geq$ .90), slightly less reliable for cardiorespiratory fitness (rxx' $\geq$ .80), and lower for musculoskeletal fitness (rxx' $\geq$ .70). Importantly, the reliabilities were approximately the same for expert testers. Musculoskeletal reliabilities were lower than other fitness dimensions.

Manual: Plowman 2013	FITNESSG GRAM	The reliability of the three field tests of aerobic capacity is, for the most part, high.<br>Values from the Anderson, et al. study with younger children (ages6-10) were lower (R = .70), but this is not unexpected for this young age group.<br><br>While many studies have evaluated full length push-ups without a cadence, several have investigated the reliability of the 90° push-up in elementary school children (Saint Romain & Mahar, 2001; Tomson, 1992; Zorn, 1992). These values (.64 to .99) are acceptable, although the total sample size is small.<br><br>No validity or reliability data are available for the shoulder stretch.	Patterson et al. reported test-retest reliability of R = .89 and R = .86 for 10-12 year old boys and girls, respectively. Reliability for a single trial was reduced to R = .80 for boys and R = .75 for girls<br>when the values were obtained from teacher-counted scores. Reliability of child-reported scores<br>were R = .82 and R = .81 (test-retest) and R = .70 and R = .69 (single trial) for boys and girls, respectively. Child-reported scores were significantly higher than teacher-reported scores.	Abdominal Strength/Endurance:<br>Anderson (1997): R = .70 knees flexed, feet free 20 rpm curl-up<br><br>Patterson (2001) :<br>R = .89 FG curl-up test-retest<br>R = .86<br>R = .80<br>R = .75 single trial, teacher scored<br><br>Upper Arm and Shoulder Girdle Strength/Endurance:<br><br>Cotton (1990): Modified pull up<br>R = .88 R = .79<br>R = .89 R = .81<br>R = .75 R = .59<br>R = .88 R = .78<br>R = .90 R = .82<br>R = .92 R = .86<br><br>McManis (2000):<br>90 degree push up<br>R = .71*<br>R = .64*	Abdominal Strength/Endurance:<br>Cureton (1975): r = .60 legs straight, feet held, N to<br>max of 100<br><br>Safrit (1987): r = .62 knees flexed, feet; r = .64<br><br>Maggnussun (1957): r = .82<br><br>Tomson (1992):<br>r = .75 knees flexed, feet<br>r = .88 free, arms crossed, 1 min<br>r = .68 knees flexed, feet<br><br>Upper Arm and Shoulder Girdle Strength/Endurance:
Barnett 2015	GSPA		Test retest reliability on 19 children indicated moderate stability (ICC = 0.60, 95% CI 0.23, 0.82).<br><br>The mean total golf score was significantly higher on the five-day retest (M difference 1.42, SD 2.46, p = 0.021)	The ICC for intra-rater agree-ment indicated close to excellent agreement (ICC = 0.79, 95% CI 0.59, 0.90)<br><br>The mean total golf score did not significantly differ between intra-rater assessments (M live assessment = 13.32, SD = 3.36, M video assessment = 13.14, SD = 2.69, p = 0.668).	
Valentini 2014	MABC-2	The Cronbach's alpha coefficient (N = 844 children) analyses showed good reliability in the 3 subscales (α = .78), as well as for the standard scores on each subtest independently (MD: α = .77; BS: α = .52; B: α = .77). These findings confirm the internal consistency of the MABC-2.	A similar trend was observed for the 3 subtest standard scores (MD: r = .67, p < .0001; BS: r = .52, p < .0001; B: r = .53, p < .0001). The ICC values were also high for the standard score (.85), and moderate for the subtests (MD: r = .59; BS: r = .61; B: r = .69).	The intra-rater reliability (ICC values from .68 to .85) indicated strong and congruent results among the evaluators<br>Ball Skills<br>ICC = 0.71<br>Balance<br>ICC = 0.72	The inter-rater (ICC values from .86 to .99)<br>Ball Skills<br>ICC = 0.86–0.92<br>Balance<br>ICC = 0.88–0.99
Kita 2014	MABC-2	Cronbach’s alpha coefficient for the eight test items was 0.602, indicating acceptable internal consistency<br>Catching with Two Hands<br>r = 0.54, α = 0.56<br>Throwing Beanbag onto Mat<br>r = 0.50, α = 0.58<br>One-Board Balance r = 0.57, α = 0.53<br>Walking Heel-to-Toe Forwards<br>r = 0.53, α = 0.55<br>Hopping on Mats r = 0.48, α = 0.54			

Holm 2013	MABC-2			The raw scores from the two tests at the first test day are given in Table 3, the mean TTS increased from Test 1 to Test 2. Table 3 also shows the results from the reliability analysis. The ICC values ranged from 0.35 to 0.67. The items “treading lace” and “one-board balance” (both preferred and non-preferred legs) also showed the highest measurement errors concerning inter-tester reliability and highest values both for the SEM and the SDC. The SDC values indicated that a change of greater than 18.5 or 4.5 for the TTS and TSS, respectively, would be required to be 90% certain that a change would not be the result of measurement error, but of a real change<br>Catch<br>ICC = 0.48 (0.15, 0.72)<br>SEM = 1.5<br>SDC = 3.5<br>Throw<br>ICC = 0.59 (0.29, 0.79)<br>SEM = 1.0<br>SDC = 2.3<br>Aim and Catch<br>ICC = 0.49 (0.17, 0.72)<br>SEM =.24<br>SDC = 5.7<br>Right leg Balance<br>ICC = 0.56 (0.26, 0.77)<br>SEM = 4.0<br>SDC = 9.6<br>Left leg Balance<br>ICC = 0.70 (0.45, 0.85)<br>SEM = 5.3<br>SDC = 12.7<br>Heel to toe walking<br>ICC = 0.75 (0.53, 0.87)<br>SEM = 0.9<br>SDC = 1.9<br>Left leg Hop<br>ICC = 0.24 (−0.15, 0.56)<br>SEM = 0.6<br>SDC = 1.5<br>Balance<br>ICC = 0.49 (0.15, 0.72)<br>SEM = 0.27<br>SDC = 6.4	The raw scores from the two tests included in the intra-tester analysis are given in Table 2. The ICC values ranged from 0.23 to 0.76. The items “treading lace” and “one-board balance” (both preferred and non-preferred legs) showed the highest measurement errors. The SDC values indicated that a change of greater than 9.7 or 2.6 for the TTS and TSS, respectively, would be required to be 90% certain that a change would not be the result of intra-tester variability or measurement error, but of a real change<br>Catch<br>ICC = 0.66 (0.40, 0.82)<br>SEM = 1.3<br>SDC = 3.1<br>Throw<br>ICC = 0.62 (0.33, 0.80)<br>SEM = 1.1<br>SDC = 2.5<br>Aim and Catch<br>ICC = 0.77 (0.56, 0.89)<br>SEM = 2.0<br>SDC = 4.7<br>Right leg Balance<br>ICC = 0.39 (0.05, 0.65)<br>SEM = 5.8<br>SDC = 13.7<br>Left leg Balance<br>ICC = 0.50 (0.19, 0.73)<br>SEM = 7.3<br>SDC = 17.4<br>Heel to toe walking<br>ICC = 0.42 (0.06, 0.67)<br>SEM = 1.6<br>SDC = 3.1<br>Balance<br>ICC = 0.29 (−0.07, 0.58)<br>SEM = 4.5<br>SDC = 10.6
Ericsson 2008	MUGI	In estimates of reliability with Cronbach’s alpha, the following values were obtained:<br>Balance/ bilateral coordination= 0.76<br>Eye-hand Coordination = 0.65<br>Total = 0.80	Despite the difficulties mentioned in repeating tests, the correlation for the three observers showed good concordance in evaluations between the two measurement times, an average of 0.78 with Spearmans rank correlation		Between different observers’ evaluations was 0.75, using Spearman’s rank correlation<br>N= 22 pupils with the same observers on two different occasions one week apart
Zuvela, 2011	POLYGON			The ICC for the object control skills area ranged between 0.88 - 0.96. In surmounting obstacles skills area, the	

				ICC ranged between 0.92 - 0.97. The ICC in resistance overcoming skills area ranged between 0.83 - 0.94 while in the space covering skills area the same coefficients ranged between 0.89 - 0.95.<br>Rolling the ball by hand to the wall ICC = 0.88<br>Dribbling the football around cones ICC = 0.89<br>Rolling the handball around cones ICC = 0.89<br>Tossing and catching the tennis ball against a wall<br>ICC = 0.96<br>Tossing and catching the volleyball against a wall<br>ICC = 0.92<br>Running across obstacles<br>ICC = 0.96<br>Skipping across obstacles<br>ICC = 0.97<br>Crawling through obstacles<br>ICC = 0.93<br>Single-leg hops<br>ICC = 0.93<br>Jumping over and through obstacles<br>ICC = 0.92<br>Rolling a tube backwards<br>ICC = 0.85<br>Carrying the medicine balls<br>ICC = 0.90<br>Carrying the BOSU ball around a cone ICC=0.94<br>Rolling the ball around cones<br>ICC = 0.92<br>Pulling the bag<br>ICC = 0.83<br>Lifting the medicine ball onto a table<br>ICC = 0.94<br>Chest crawling<br>ICC = 0.93<br>Straight running<br>ICC = 0.95<br>Rolling sideways<br>ICC = 0.89<br>Changing course running<br>ICC = 0.92<br>Beam walking<br>ICC = 0.94	
Myers 2015	PARAGON		The test-retest reliability was based on 7 observers overall averages across all 5 categories (r=0.94) specifically calculated for motions (r=0.93)		To establish inter-rater reliability, 6 field reliability checks were conducted in which two observers simultaneously observed and recorded activities of the same focal child, for 24 epochs. Percentage agreement and Ebel's intra-class correlation was calculated: percentage agreement specifically

					for motions was 80% and Ebel for PARAGON was 0.97
Bachev, 2014	SLALOM	Pearson’s correlation coefficient: 0.8			
Calatayud, 2014	SEBT		Paired t tests revealed no significant differences between the scores of the 2 testing sessions for all directions reached in both limbs (P. 0.05).<br>Mean, standard deviation, and P values of the paired t-test for normalized maximum distances reached in both limbs are reported in:<br>The ICC values for normalized scores showed moderate to good reliability (0.51 to 0.93), and the SEM values for normalized scores ranged from 3.03 to 12.32.<br>Anterior left stance:<br>ICC = 0.53, SEM = 8.36, MD = 23.19<br>Anterior right stance:<br>ICC = 0.87, SEM = 3.03, MD = 8.39<br>Posteromedial left stance<br>ICC = 0.51, SEM = 12.32, MD = 34.15<br>Posteromedial right stance<br>ICC = 0.93, SEM = 3.85, MD = 10.66<br>Posterlateral left stance<br>ICC = 0.91, SEM = 3.56, MD = 9.88<br>Posterolateral right stance<br>ICC = 0.92, SEM = 4.20, MD = 11.63		
Rudd 2015	SS		Test re-test reliability over a seven day period also demonstrated excellent consistency for each of the three skills: rock (ICC = 0.95; 95% CI: 0.83–0.98), log roll (ICC = 0.87; 95% CI: 0.59–0.95) and back support (ICC = 0 .88; 95% CI: 0.65–0.96).		The Intra Class Correlations (ICC) for inter-rater reliability were all good: locomotor skills (ICC = 0.90; 95% CI: 0.73–0.98), object control skills (ICC = 0.82; 95% CI: 0.58–0.96), and rock: (ICC = 0.87; 95% CI: 0.73–0.94), log roll (ICC = 0.81; 95% CI: 0.52–0.93) and back support (ICC = 0.87; 95% CI: 0.72–0.95).
Faigenbaum 2015	YBT	The overall ICC was moderate-to-good for the anterior (right=0.82; left=0.82), posteromedial (right=0.77; left=0.75), and posterolateral (right 0.80; left=0.77) reach directions. The combined ICC was also moderate-to-good for children in grades 1 (0.71), 2 (0.74), 3 (0.84), 4 (0.82), and 5 (0.79).			Interrater reliability within session (ICC > 0.995) and between sessions (0.907 ≤ ICC ≤ 0.974) were both excellent.
Examiners manual Ulrich 2019	TGMD-3	Manual pg.39 onwards<br>7 years (locomotor α = 0.90, ball skills α = 0.88, gross motor α = 0.93)<br>8 years (locomotor α = 0.87, ball skills α = 0.87, gross motor α = 0.97)<br>9 years (locomotor α = 0.87, ball skills α = 0.85, gross motor α = 0.81)<br>10 years (7 years (locomotor α = 0.89, ball skills α = 0.86, gross motor α = 0.92)	Test retest<br>Locomotor skills<br>ICC = 0.85<br>Balls skills<br>ICC = 0.87<br>Gross motor<br>ICC = 0.90	Intrarater reliability was examined by comparing scores form the first rating to the second rating for each rater individually. The resulting coefficients for the subtests and total scores were above 0.90.	Interrater reliability was examined by calculating intraclass coefficients for each subtest and total score across five raters.
Bisi, 2017	TGMD-3				Raw Score<br>Mean Difference = 2.0<br>Standard Score<br>Mean Difference = 0.8

Wagner, 2017	TGMD-3	Locomotor skills<br>α = 0.76<br>Ball skills<br>α = 0.89	Locomotor skills<br>ICC = 0.94 (0.91, 0.96)<br>Balls skills<br>ICC = 0.98 (0.97, 0.99)	Locomotor skills<br>ICC = 0.97 (0.94, 0.99)<br>Ball skills<br>ICC = 0.99 (0.98, 1.00)	Locomotor skills<br>ICC = 0.88 (0.76, 0.95)<br>Ball skills<br>ICC = 0.97 (0.94, 0.99)
Maeng et al., 2017	TGMD-3	Total<br>α = 0.96<br>Locomotor<br>α = 0.92<br>Object Control<br>α = 0.95	Total<br>ICC = 0.97<br>Locomotor<br>ICC = 0.97<br>Ball Skills<br>ICC = 0.95		98%
**Cognitive Domain**					
Economos et al 2010	BONES<br>PAS		Two trained research assistants administered the PAS independently, once to each child on the same day, at least 1-2 hours apart (referred to as T1 and T2. For the questions in the knowledge component, the κ-statistic percent agreement ranged from 60.5% (hopping) to 97.4% (running). Knowledge in 'bone smart' PA:<br>jumping k=86.8%<br>running k=97.4%<br>bike riding k=89.7%<br>hopping, k=60.5%<br>playing on playground k=73.7%<br>skipping k=68.4%<br>computer/video games k=68.4%<br>drawing/colouring k=75.7%<br>watching TV k=89.5%		
Manios et al., 1999	PHKA		ICC or weighted Kappa not reported<br>The test–retest reliability of the questionnaire was assessed administering the questionnaire to a subsample of 35 first grade and 35 fourth-grade pupils with a 2-week interval. With the use of the paired-test, no significant difference was found between the first assessment and the re-the re-examination results (P=0.23 for fourth graders)		

Achievement Goal scale for Youth Sports (AGSYS); Attitudes Towards Curriculum Physical Education (ATCPE);Attitudes Towards Outdoor play scale (ATOP); Adapted Behavioural Regulation in Exercise Questionnaire (BREQ); Children's Attraction to Physical Activity Questionnaire (CAPA); Children's Attitudes Towards Physical Activity (CATPA); Commitment to Physical Activity Scale (CPAS); Children and Youth Physical Self-Perception Profile (CY-PSPP); Motivational determinants of elementary school students' participation in physical activity (DPAPI); Enjoyment in Physical Education (EnjoyPE); Food, Health and Choices Questionnaire (FHC-Q); Feelings About Physical Movement (FAPM); Healthy Opportunities for Physical Activity and Nutrition Evaluation (HOP'N); Lunchtime Enjoyment of Activity and Play Questionnaire (LEAP); Momentary Assessment of Affect and Physical feeling states (MAAP);Motivational Orientation in Sport Scale (MOSS); Negative Attitudes Towards Physical Activity Scale (NAS); Physical Activity Beliefs and Motives (PABM); Physical Activity Enjoyment Scale (PACES); Physical activity and Healthy Food Efficacy (PAHFE); Positive Attitudes Towards Physical Activity Scale (PAS); Physical Activity Self-Efficacy Questionnaire (PASE); Physical Activity Self-Efficacy Scale (PASES); The Revised Perceived Locus of causality in physical Education (PLOC in PE); Perceived Motivational Climate in Sport Questionnaire (PMCS); Response to Challenge Scale (RCS);Self-Perception Profile for Children (SPPC); Trichotomous Achievement Goal Model (TAGM); Task and Ego Orientation in Sport Questionnaire (TEOSQ); ALPHA Fitness Battery (ALPHA); Athletic Skills Track (AST) ½; Bruininks–Oseretsky Test of Motor Proficiency (BOTMP-SF); Canadian Agility and Movement Skills Assessment (CAMSA); EUROFIT; FITNESSGRAM (FG); FG-COMPASS (FGCOMP); Golf Swing and Putt skill Assessment (GSPA); Motorische Basiskompetenzen in der 3 (MOBAK-3); Movement assessment battery for children-2 (MABC2); Motorisk Utveckling som Grund för Inlärning (MUGI); Obstacle Polygon (OP); PA Research and Assessment tool for Garden Observation (PARAGON); Slalom Movement Test (SMT); Star Excursion Balance Test (SEBT); Stability skill test (SS)Test of Gross Motor Development-3 (TGMD-3); The Leger 20m Shuttle Run test (20MSR); Y Balance Test (YBT); Beat Osteoporosis Now-Physical Activity Survey (BONES-PAS); Pupil Health Knowledge Assessment (PHKA); Response to Challenge (RCS); Canadian Assessment of Physical Literacy (CAPL-2); Passport for Life (PFL)
